# Expenditure and Nutritional Impact of Banning the Promotion of Foods High in Fat, Sugar and Salt in Scotland

**DOI:** 10.3389/fnut.2022.874018

**Published:** 2022-06-29

**Authors:** Cesar Revoredo-Giha, Paul McNamee, Patricia Norwood, Faical Akaichi, Wisdom Dogbe

**Affiliations:** ^1^Department of Rural Economy, Environment and Society, Scotland's Rural College, Edinburgh, United Kingdom; ^2^Health Economics Research Unit, Institute of Applied Health Sciences, University of Aberdeen, Aberdeen, United Kingdom; ^3^Rowett Institute of Health and Nutrition, University of Aberdeen, Aberdeen, United Kingdom

**Keywords:** food promotions, demand analysis, Scotland, HFSS foods, public policy

## Abstract

The purpose of the paper is to provide an ex-ante evaluation of banning price promotions for discretionary foods (e. g., such as confectionary, crisps, biscuits, sweet and savory snacks, cakes) in Scotland. The methodology consisted of the estimation of demand systems by socioeconomic groups (i.e., lifestage and income groups) for 19 food groups using a highly product disaggregated dataset. These results were used to simulate scenarios consisting of eliminating price promotions on the discretionary food products for the entire sample and by group and analyzing nutritional results. The results indicated a net impact of reducing energy by 651 kcal per capita per week (C.I. −695, −608)^1^. Similar results were found for macro nutrients. There were some significant differences across different income and lifestage groups, with kcal energy reductions being significantly greater amongst household with lower income, and in households where respondents were aged 45 years or over. The analysis concluded that restrictions on the promotion of foods considered to be high in saturated fat, sugar, or salt (HFSS) are seen as one measure to improve the overall nutritional quality of foods consumed. Results indicate that restricting promotions has the potential to reduce the number of calories, sugar, saturated fats and sodium for most food groups.

## Introduction

An enduring issue for all health care systems is that increasing demand for health care continues to outstrip the amount of resources available. There are many reasons for this excess demand, but one key factor relates to the health behavior of the population in terms of engagement with a healthy lifestyle (1, 2). Poor diet and low levels of physical activity increase the likelihood that an individual will become overweight or obese, and the subsequent impact on health care systems and the economy is a growing concern (3, 4). Changing behavior is challenging however, as highlighted in the Foresight Report (1), where it was noted that “obesity is linked to broad social developments and shifts in values, such as changes in food production, motorized transport and work/home lifestyle patterns… the technological revolution of the 20th century has left in its wake an ‘obesogenic environment”'.

A key policy implication that arises from recognition of the strong relationship between environmental factors and obesity is that universal policies that apply to whole population groups, such as taxation, subsidies, regulation, reformulation and labeling are now being more heavily promoted, developed and tested in many countries (5–7). Recent examples include the introduction of a new tax on sugar-sweetened beverages in the UK from 2018 (8, 9). An additional policy being considered in some countries is restrictions (i.e., banning) on the promotion or advertising of foods considered to be high in saturated fat and/or sugar and/or salt (10–12). For example, within Scotland, as part of a Diet and Healthy Weight Delivery Plan (13), policy-makers are considering the introduction of new restrictions on the advertising of price promotions such as multi buys and other promotions of value (e.g., temporary price reductions, buy one get one free, Y for £X such as 2 of the products for £5) for discretionary foods such as confectionary, crisps, biscuits, sweet and savory snacks, cakes, pastries and puddings, some non-alcoholic drinks, and ice-creams (14). However, to the best of our knowledge, currently there is no published evidence available of the effectiveness of such banning on household expenditure or on the impact on overall calorie and nutrient intake. The aims of this paper, therefore, are to estimate the reduction in the purchase of discretionary foods (and associated reductions in calories, total fat, saturated fat, sugar and salt) that could arise as a consequence of banning the promotion and marketing of discretionary foods, after taking into account changes in the purchase of other non-discretionary foods. In addition, the impact on different households, categorized by income and demographic structure, is also assessed.

## Methods

### Data

Data from the Kantar Worldpanel (KWP) for Scotland from 2013 to 2018 were used. This dataset provides information about purchases at the level of products by households and whether they were made under a promotion (e.g., x GBP pounds less). It also includes product nutrient data (i.e., back or side of packaging nutrition). The available nutrients in the dataset were calories, proteins, carbohydrates, sugar, fats, saturates, fiber and sodium.

The KWP data are the largest available source of individual household data for Great Britain. KWP is a continuous consumer panel which is geographically and demographically representative of Great Britain. KWP recruits a representative sample of households with respect to household size, number of children, social class, geographical region and age group and collects socio-demographic data such as household income, occupational class and education. KWP panel members scan and report all purchases of food and drink brought into the home, as well as providing till receipts which verify the purchases made and provide price information. The KWP dataset covers all outlets where purchases of food and drink to bring home can be made. This includes supermarkets, corner shops and online purchases. It does not include purchases that are consumed away from home, although these only account for about 10% of total food and drink energy (15, 16).

Nineteen food categories were included in the analysis and were classified into categories of discretionary or non-discretionary foods. Discretionary foods are defined as foods and drinks not necessary to provide the nutrients the human body needs, but that may add variety to a person's diet (14); they include take home confectionery, biscuits, take home savories, cakes, pastries, and sugar morning goods, total puddings and desserts, take home sugary drinks, edible ices and ice creams. Non-discretionary foods are all other remaining foods and include dairy products, meat and fish, fats and eggs, fruit, vegetables, grains, prepared ready to eat foods, sugar and preserves, condiments and sauces, low calorie soft drinks and juices, alcoholic beverages and a numeraire category including all the other products (i.e., non-food).

The total number of observations for the analysis was 9,737 households. We calculated annual household food expenditure data, and to eliminate the difference in the number of weeks that households were observed, the data were expressed as per capita weekly averages. Only households that were observed a minimum of 40 weeks in a year were included in the analysis. To examine socioeconomic differences in household expenditure, households were classified into five household gross income bands (under £30,000, £30,000–£39,999, £40,000–£49,999, £50,000–£59,999, £60,000 or over) and five life stage groups: pre-family, young family, middle family, older family and 45+ without children^2^ The sample characteristics are presented in Table 1.

**Table 1:** Sample characteristics.

	**Age**	**Sex**	**Number of children**	**Number of adults**	**BMI**	**Total** **weekly** **expenditure** **per capita (£)**
**Entire sample (9,737 observations)**						
Mean	50.95	0.28	0.49	2.07	22.66	27.50
Standard deviation	13.47	0.45	0.86	1.88	11.63	14.31
**Income groups**						
**£0–£29,999 (5,685 observations)**						
Mean	53.80	0.28	0.38	1.95	23.20	28.45
Standard deviation	13.66	0.45	0.77	2.37	12.01	15.03
**£30,000–£39,999 (1,701 observation)**						
Mean	48.17	0.30	0.55	2.24	22.06	26.28
Standard deviation	12.65	0.46	0.86	0.78	10.74	13.09
**£40,000–£49,999 (1,168 observations)**						
Mean	47.18	0.26	0.65	2.15	21.58	26.40
Standard deviation	12.60	0.44	0.95	0.67	11.39	12.72
**£50,000–£59,999 (619 observations)**						
Mean	43.85	0.21	0.83	2.33	22.25	25.54
Standard deviation	9.76	0.41	1.09	0.83	11.70	14.04
**£60,000–over (564 observations)**						
Mean	46.19	0.31	0.69	2.34	21.77	25.92
Standard deviation	11.14	0.46	0.95	0.77	10.43	13.02
**Lifestage groups**						
**Pre-family (1,327 observations)**						
Mean	35.01	0.34	0.00	1.86	21.05	28.35
Standard deviation	6.25	0.48	0.00	0.83	12.06	15.40
**Young family (1,148 observations)**						
Mean	35.94	0.16	1.83	2.06	19.90	17.96
Standard deviation	6.47	0.36	0.83	0.54	11.74	7.34
**Middle family (789 observations)**						
Mean	41.12	0.20	1.81	2.03	20.35	19.84
Standard deviation	6.55	0.40	0.78	0.61	12.66	7.55
**Older family (873 observations)**						
Mean	45.61	0.21	1.26	2.36	21.52	19.80
Standard deviation	6.59	0.40	0.49	0.82	12.54	7.54
**45+** **no children (5,600 observations)**						
Mean	60.02	0.31	0.00	2.09	24.11	31.53
Standard deviation	8.83	0.46	0.00	2.40	10.97	14.82

*Own elaboration based on Kantar Worldpanel data. Sex was tabulated as 0 if the person answering the information is female and 1 if is male*.

### Statistical Analysis

A simulation approach was used to identify the potential impact of removal of current promotions of discretionary foods. The estimates are derived from a method published in Drèze et al. (17), who used a modified version of Deaton and Muellbauer's Almost Ideal Demand System (AIDS) consumer demand model (18).

The AIDS model involves estimation of the expenditure shares (proportions with respect to the total grocery shopping expenditure across the 19 food categories, plus a miscellaneous category for all non-food purchases) of consumers' budget spent on each food and non-food category. These are estimated as a function of prices, total expenditure on groceries in real terms and an indicator of whether products within categories are on promotion. Thus, we calculate a value for the expenditure, price, and promotion of each food category for each household. For each category, the share equation consists of the following effects:


$$\begin{array}{l} \left(\begin{array}{c} \text{Category}\\ \text{share} \end{array}\right)=\left(\begin{array}{c} \text{Price}\\ \text{effect} \end{array}\right)+\left(\begin{array}{c} \text{Income}\\ \text{effect} \end{array}\right)+\left(\begin{array}{c} \text{Promotion}\\ \text{advertising}\\ \text{effect} \end{array}\right) \end{array}$$

The simulation involved the subtraction of the promotions advertising effect and then re-computing the category shares, keeping the income and prices constant. Total expenditure per category was then calculated by multiplying the new shares by the total expenditure on groceries. Dividing the expenditure by the average price of the category provided the resulting quantities. To compute the energy and nutrients, the quantities were multiplied by the energy and nutrient coefficients.

Following (19), expenditure, price and promotion values were computed as follows:

Category Expenditure ${\text{Y}}_{\text{gt}}^{\left(\text{h}\right)}$


(1) $${\text{Y}}_{\text{gt}}^{\left(\text{h}\right)}\text{=}\sum_{\text{s=1}}^{\text{s}}{\text{p}}_{\text{st}}.{\text{q}}_{\text{st}}^{\left(\text{h}\right)}$$

Category Price ${\text{P}}_{\text{gt}}^{\left(\text{h}\right)}$


(2) $${\text{P}}_{\text{gt}}^{\left(\text{h}\right)}\text{=}\sum_{\text{s=1}}^{\text{s}}{\text{p}}_{\text{st}}.{\text{w}}_{\text{s}}^{\left(\text{h}\right)}$$

Category Promotion ${\text{Pm}}_{\text{gt}}^{\left(\text{h}\right)}$


(3) $${\text{Pm}}_{\text{gt}}^{\left(\text{h}\right)}\text{=}\sum_{\text{s=1}}^{\text{s}}{\text{pm}}_{\text{st}}.{\text{w}}_{\text{s}}^{\left(\text{h}\right)}$$

Where:

${\text{Pm}}_{\text{gt}}^{\left(\text{h}\right)}$ = 1 if product s was on promotion at time t; 0 otherwise.

p_st_ = price of product s during time t.

${\text{q}}_{\text{st}}^{\left(\text{h}\right)}$ = quantity of product s bought by household h at time t.

s = number of individual products in category g.

t = time period from 1...T.

The weights associated with products, ${\text{w}}_{\text{s}}^{\left(\text{h}\right)},\;$will be calculated as follow:


(4) $${\text{w}}_{\text{s}}^{\left(\text{h}\right)}\text{=}\frac{\sum_{\text{t=1}}^{\text{T}}{\text{p}}_{\text{st}}{\text{q}}_{\text{st}}^{\left(\text{h}\right)}}{\sum_{\text{t=1}}^{\text{T}}\;\sum_{\text{k=1}}^{\text{S}}{\text{p}}_{\text{kt}}{\text{q}}_{\text{kt}}^{\left(\text{h}\right)}}$$

The model comprised the estimation of the shares of consumers' budget spent on category g in time t (W_gt_) given by eq. (5):


(5) $$\begin{array}{l} {\text{w}}_{\text{gt}}^{\left(\text{h}\right)}\text{=}\alpha_{\text{g}}\text{+}\sum_{\text{j=1}}^{\text{n}}\beta_{\text{gj}}{\text{lnP}}_{\text{jt}}^{\left(\text{h}\right)}\text{+}\theta_{\text{g}}ln\left(\frac{{\text{X}}_{\text{t}}^{\left(\text{h}\right)}}{{\bar{\text{P}}}_{\text{t}}^{\left(\text{h}\right)}}\right)\text{+}\sum_{\text{j=1}}^{\text{n}}\delta_{\text{gj}}{\text{Pm}}_{\text{jt}}^{\left(\text{h}\right)}\\ \text{ +}\varepsilon_{\text{gt}}^{\left(\text{h}\right)} \end{array}$$

where ${\text{w}}_{\text{gt}}^{\left(\text{h}\right)}$ is the expenditure share allocated to category g by household h, ${\text{P}}_{\text{jt}}^{\left(\text{h}\right)}$ are the prices encountered by household h for each of the n categories (j = 1..n), ${\text{X}}_{\text{t}}^{\left(\text{h}\right)}$ is the expenditure of household h and ${\bar{\text{P}}}_{t}^{\left(h\right)}$ is a price index.

The use of average prices (as in the case of unit values, i.e., the ratio between expenditure and quantity) because it contains a price component and a quality choice component [e.g., Deaton, (20)], as the same food category is generally available at different quality and price levels. When aggregated prices are used as regressors in demand equations, they generate an endogeneity problem producing biased parameters. To solve the issue, this paper follows the approach by Capacci and Mazzocchi (21). They started from the following equation for each price category:


(6) $${\text{lnP}}_{\text{j}}^{\left(\text{h}\right)}\text{=}\omega_{\text{j0}}\text{+}\sum_{\text{l=1}}^{\text{L}}\omega_{\text{jl}}{\text{z}}_{\text{lh}}\text{+}\mu_{\text{j}}\ln{\text{q}}_{\text{jh}}{\text{+lnp}}_{\text{j}}{\text{+u}}_{\text{jh}}$$

Where z_lh_ are household characteristics variables, q_jh_ are quantities and p_j_ are the prices; ω_*j*0_, ω_*jl*_ and μ_j_ are parameters and u_jh_ is a normal error term. Equation (6) decomposes the aggregation in: the quality, the price and the quantity effects. Assuming that a cluster of households which belongs to the same geographical area and time period face the same prices, it is possible to estimate the parameters using a within cluster equation (where k indicates the cluster) due to the fact that prices and the category-specific coefficient ω_*j*0_ are canceled out. Thus, prices were estimated as in eq.:


(7) $$\ln{\hat{\text{P}}}_{\text{jk}}^{\left(\text{h}\right)}=\ln P_{\text{j}}^{\left(\text{h}\right)}-\sum_{\text{l=1}}^{\text{L}}{\hat{\omega }}_{\text{jl}}{\text{z}}_{\text{lk}}-{\hat{\mu }}_{\text{j}}\ln{\text{q}}_{\text{jk}}$$

The price index ${\bar{\text{P}}}_{\text{t}}^{\left(\text{h}\right)}$ was approximated by the Stone price index (i.e. $\text{ln}\sum_{\text{g=1}}^{\text{n}}{\text{w}}_{\text{gt}}^{\left(\text{h}\right)}\ln{\text{P}}_{\text{gt}})$, making the budget share equation to be linear in the parameters. The system (22) was estimated by iterative seemingly unrelated regressions and imposing constraints related to adding up, homogeneity and symmetry (8)^3^:


(8) $$\sum_{\text{g=1}}^{\text{n}}\alpha_{\text{g}}\text{=1};\sum_{\text{g=1}}^{\text{n}}\beta_{\text{gj}}\text{=0};\sum_{\text{g=1}}^{\text{n}}\theta_{\text{g}}\text{=0};\sum_{\text{g=1}}^{\text{n}}\delta_{\text{gj}}\text{=0}$$

For any lifestage group, the nutritional changes for nutrient i, for food category g, due to the measures were evaluated at the means of the variables using the following formula (9):


(9) $$\begin{array}{l} {\text{N}}_{\text{ig}}=\left[\frac{\left(-\overset{D}{\underset{j=1}{\sum }}\delta_{gj}\cdot {\bar{Pm}}_{j}\right)\cdot \bar{X}}{{\bar{P}}_{g}}\right]\cdot {ℵ}_{ig} \end{array}$$

Where D is the number of discretionary categories, ${\bar{\text{Pm}}}_{j}$ is the average promotion for food category j, $\bar{\text{X}}$ is the average expenditure, ${\bar{\text{P}}}_{g}$ is the average price of category g and ℵ_ig_ is nutrient i coefficient (e.g., saturates per 100 grams) of category g. Note that promotions for products that are not discretionary are supposed to still be in place.

## Results

Table 2 shows the evolution of the share of sales of discretionary foods at full price (i.e., no promotion) and under each type of promotion from 2013 to 2018. There are two important trends. First, an increasing proportion of foods are sold at full price (the only exceptions are edible ices and ice creams). Second, temporary price reductions are more common in comparison to other types of promotions; across all years they are the most common type of promotion for all discretionary categories.

**Table 2:** Share of sales at full price and promotion by discretionary foods.

	**Years**					
	**2013**	**2014**	**2015**	**2016**	**2017**	**2018**
**Take home confectionery**						
Full price	51.6	52.2	52.3	54.1	58.1	59.0
Temporary price reduction	36.9	37.1	38.0	36.2	33.2	32.9
Multibuy	0.9	1.0	0.4	0.6	0.3	0.3
Y for £X	9.8	9.1	8.8	8.6	8.1	7.7
Other promotions	0.7	0.5	0.5	0.5	0.3	0.2
**Biscuits**						
Full price	56.5	54.2	51.7	55.2	59.7	60.5
Temporary price reduction	35.7	35.2	36.3	37.1	35.3	34.0
Multibuy	1.4	0.5	0.4	0.1	0.2	0.9
Y for £X	5.4	9.2	10.8	6.7	4.0	4.2
Other promotions	1.0	1.0	0.8	0.9	0.8	0.5
**Take home savories**						
Full price	48.8	47.4	48.9	48.3	52.8	54.2
Temporary price reduction	29.1	31.0	35.5	38.6	35.9	35.2
Multibuy	3.7	3.6	0.9	0.4	0.2	0.0
Y for £X	17.4	17.1	14.3	11.9	10.3	9.9
Other promotions	1.0	0.9	0.4	0.8	0.9	0.6
**Ambient cakes and pastries**						
Full price	71.6	71.6	71.0	73.2	75.2	77.1
Temporary price reduction	15.5	15.4	18.5	19.0	18.0	16.4
Multibuy	0.4	0.6	0.2	0.3	0.3	0.3
Y for £X	12.0	11.9	10.1	7.3	6.4	6.0
Other promotions	0.5	0.5	0.2	0.2	0.1	0.1
**Total puddings and desserts**						
Full price	57.5	56.4	56.4	57.7	61.0	62.8
Temporary price reduction	21.2	22.7	24.3	25.3	22.4	24.6
Multibuy	2.3	1.1	0.6	0.9	1.8	1.4
Y for £X	15.2	15.8	14.2	11.0	9.3	8.5
Other promotions	3.7	4.0	4.5	5.1	5.5	2.6
**Regular soft drinks**						
Full price	46.8	46.5	45.6	47.6	53.3	57.6
Temporary price reduction	26.0	25.6	27.2	30.2	31.4	30.0
Multibuy	4.4	2.7	1.9	0.2	0.1	0.0
Y for £X	21.4	23.6	24.1	20.6	13.8	11.0
Other promotions	1.3	1.5	1.1	1.4	1.4	1.3
**Edible ices and ice cream**						
Full price	52.3	54.7	52.3	53.6	53.5	52.6
Temporary price reduction	33.2	29.9	33.0	36.0	35.4	33.8
Multibuy	0.1	0.4	0.1	0.1	0.0	0.1
Y for £X	13.6	14.2	12.7	8.4	9.5	12.3
Other promotions	0.8	0.8	1.9	1.9	1.5	1.2

*Own elaboration based on Kantar Worldpanel data. Additional information can be seen in the Supplementary Tables*.

Supplementary Tables 13–19 present the information about the importance (share) of the sales under the different promotions on the total sales of discretionary foods for the period 2013 to 2018. They also measure the contribution of promotions to the sales of each category. All the categories, except for “Edible ices and ice cream”, show that an increasing proportion is being sold under no promotion (i.e., at full price). In the case of Edible ices and ice cream, the proportion sold under full price remained stable during the period.

Considering the different types of promotions, temporary price reductions appear to be the most important type, but there are some differences by category. For 2018, the share of temporary price reduction ranges from 16.4 per cent (Supplementary Table S16, Ambient cakes and pastries) to 35.2 per cent (Supplementary Table S15, Take home savories), although in most of the categories the proportion is above 30 per cent. Y for £X promotions are next in importance but appear much lower in share compared with temporary price reductions.

The contribution to growth analysis breakdowns the growth on each category by the contribution of full price and the different promotions. There is no consistent pattern across categories and over time (i.e., a type of promotion that always contributes positively to sales).

Table 3 presents the net change (i.e., the sum of changes in discretionary and non-discretionary foods) in energy, sugar, fat, saturates and sodium estimated to arise following a restriction of the promotion of discretionary foods. The results indicate a reduction in energy of about 651 kcal per capita per week (C.I. −695, −607), i.e., about 87.6 kcal per capita per day or 4.4 per cent of a daily diet of 2,000 kcal.

**Table 3:** Net results of the simulation on discretionary foods (Changes are in per capita per week terms).

**Group**	**Discretionary foods**	**Other** **foods** **and** **drinks**	**Net impact**			
			**Total**	**Mean** **standard** **error**	**Total confidence interval lower**	**Total** **confidence** **interval** **upper**
**All the sample (9,737 observations)**						
Δ in share	−0.041	0.038	−0.003	0.000	−0.003	−0.003
Δ in expenditure (£)	−1.135	1.056	−0.079	0.006	−0.090	−0.068
Δ in quantity (Kg)	−0.449	0.214	−0.235	0.008	−0.250	−0.220
Δ in energy (kcal)	−961.380	309.942	−651.438	22.297	−695.146	−607.731
Δ in protein(g)	−13.591	7.610	−5.981	0.441	−6.844	−5.117
Δ in carbohydrate(g)	−136.078	17.100	−118.978	3.304	−125.454	−112.502
Δ in sugar(g)	−84.500	10.657	−73.843	2.278	−78.308	−69.379
Δ in fat(g)	−40.091	18.422	−21.669	1.158	−23.938	−19.400
Δ in saturates(g)	−19.421	7.566	−11.855	0.591	−13.013	−10.697
Δ in fiber(g)	−5.374	2.609	−2.765	0.147	−3.054	−2.476
Δ in sodium(g)	−0.488	0.255	−0.233	0.015	−0.262	−0.203

*Based on Table A1 in the Annex*.

As shown in Table 3, the discretionary categories show similar results in terms of direction of change relating to energy and nutrients. The reduction in nutrients is partially compensated by the increase in quantities in non-discretionary food and drinks (i.e., other food and drinks) but not enough to offset the reductions achieved from the discretionary products. This indicates that the impact overall is a reduction of the purchase/consumption of discretionary foods considered to be high in fat, sugar and salt.

Tables 4, 5 show the results in terms of net changes in energy and nutrients by different socioeconomic classifications (income and lifestage).

**Table 4:** Net results of the simulation on discretionary foods-by income classification (Changes are in per capita per week terms).

**Group**	**Discretionary foods**	**Other** **foods** **and** **drinks**	**Net impact**			
			**Total**	**Mean** **standard** **error**	**Total confidence interval lower**	**Total** **confidence** **interval** **upper**
**£0–£29,999**						
Δ in energy (kcal)	−1,112.891	369.531	−743.360	32.112	−816.757	−669.963
Δ in sugar(g)	−102.274	11.638	−90.636	3.290	−98.194	−83.077
Δ in fat(g)	−46.004	22.641	−23.363	1.615	−27.012	−19.714
Δ in saturates(g)	−23.059	9.199	−13.860	0.837	−15.743	−11.976
Δ in sodium(g)	−0.529	0.321	−0.208	0.022	−0.258	−0.159
**£30,000–£39,999**						
Δ in energy (kcal)	−1,007.462	250.459	−757.003	36.766	−891.251	−622.756
Δ in sugar(g)	−90.208	6.699	−83.509	3.617	−97.334	−69.685
Δ in fat(g)	−41.922	15.791	−26.132	2.150	−32.805	−19.458
Δ in saturates(g)	−20.467	6.197	−14.271	1.068	−17.716	−10.825
Δ in sodium(g)	−0.494	0.198	−0.296	0.030	−0.387	−0.206
**£40,000–£49,999**						
Δ in energy (kcal)	−739.890	248.300	−491.589	63.115	−653.651	−329.528
Δ in sugar(g)	−63.582	5.809	−57.773	6.939	−74.462	−41.085
Δ in fat(g)	−31.999	18.076	−13.923	3.153	−21.979	−5.867
Δ in saturates(g)	−14.838	8.327	−6.511	1.681	−10.670	−2.352
Δ in sodium(g)	−0.385	0.231	−0.154	0.040	−0.264	−0.045
**£50,000–£59,999**						
Δ in energy (kcal)	−1,175.616	465.699	−709.917	96.253	−932.738	−487.096
Δ in sugar(g)	−103.006	17.362	−85.644	9.353	−108.590	−62.699
Δ in fat(g)	−47.212	21.171	−26.041	4.573	−37.117	−14.964
Δ in saturates(g)	−22.334	9.599	−12.735	2.338	−18.454	−7.017
Δ in sodium(g)	−0.631	0.605	−0.026	0.064	−0.177	0.124
**£60,000–over**						
Δ in energy (kcal)	−779.164	388.119	−358.963	−391.045	−624.523	−157.568
Δ in sugar(g)	−57.796	18.805	−39.696	−38.991	−63.034	−14.948
Δ in fat(g)	−33.235	24.860	−5.894	−8.375	−19.981	3.232
Δ in saturates(g)	−14.168	10.603	−2.979	−3.565	−9.557	2.427
Δ in sodium(g)	−0.482	0.237	−0.154	−0.245	−0.402	−0.087

*Based on Table A2 in the Annex*.

**Table 5:** Net results of the simulation on discretionary foods-by lifestage classification (Changes are in per capita per week terms).

**Group**	**Discretionary foods**	**Other** **foods** **and** **drinks**	**Net impact**			
			**Total**	**Mean** **standard** **error**	**Total confidence interval lower**	**Total** **confidence** **interval** **upper**
**Pre-family**						
Δ in energy (kcal)	−1,083.563	401.590	−681.973	65.745	−810.947	−553.000
Δ in sugar(g)	−97.028	14.980	−82.047	7.036	−95.850	−68.244
Δ in fat(g)	−45.336	20.314	−25.022	3.648	−32.177	−17.866
Δ in saturates(g)	−21.867	8.118	−13.748	1.834	−17.346	−10.151
Δ in sodium(g)	−0.520	0.487	−0.032	0.039	−0.109	0.044
**Young family**						
Δ in energy (kcal)	−884.890	373.499	−511.391	43.263	−649.965	−372.817
Δ in sugar(g)	−78.356	12.750	−65.606	5.004	−80.436	−50.775
Δ in fat(g)	−37.610	21.788	−15.822	2.081	−23.510	−8.134
Δ in saturates(g)	−17.804	9.195	−8.609	1.134	−12.475	−4.744
Δ in sodium(g)	−0.448	0.324	−0.124	0.019	−0.206	−0.042
**Middle family**						
Δ in energy (kcal)	−673.787	265.562	−408.226	44.861	−574.500	−241.952
Δ in sugar(g)	−60.982	5.927	−55.055	5.099	−72.850	−37.260
Δ in fat(g)	−28.068	15.824	−12.245	2.415	−21.470	−3.020
Δ in saturates(g)	−13.681	6.188	−7.493	1.274	−12.132	−2.855
Δ in sodium(g)	−0.338	0.396	0.058	0.026	−0.041	0.157
**Older family**						
Δ in energy (kcal)	−633.786	165.036	−468.750	44.869	−627.819	−309.681
Δ in sugar(g)	−59.518	3.293	−56.225	4.639	−73.249	−39.201
Δ in fat(g)	−25.152	8.437	−16.715	2.212	−25.540	−7.890
Δ in saturates(g)	−12.799	2.322	−10.476	1.167	−14.913	−6.039
Δ in sodium(g)	−0.305	0.189	−0.116	0.030	−0.210	−0.021
**45+** **no children**						
Δ in energy (kcal)	−974.065	249.670	−724.394	32.578	−787.077	−661.712
Δ in sugar(g)	−84.829	9.602	−75.227	3.133	−81.936	−68.519
Δ in fat(g)	−40.291	14.903	−25.388	1.700	−28.866	−21.910
Δ in saturates(g)	−19.986	6.508	−13.478	0.855	−15.226	−11.729
Δ in sodium(g)	−0.497	0.142	−0.355	0.025	−0.393	−0.318

*Based on Table A3 in the Annex*.

The simulations show that all the groups display a reduction in energy and nutrients. For income group, Table 4 shows that the range of net reduction in energy is from approximately 359 kcal (C.I. −624, −158) (highest income group) to about 757 kcal (C.I. −891, −623) (second lowest income group). The decrease in sugar ranges from about 39.7 g (C.I. −63, −14) (highest income group) to around 90.6 g (C.I. −98, −83) (lowest income group). The highest income group and the second lowest income groups provide the limits for fats [about 5.9 g (C.I. −20, −3.2) to 26.1 g (C.I. −33, −19)] and saturated fats [about 3 g (C.I. −10, 2.4) to 14.3 g. (C.I. −18, −11)]. In the case of sodium there are few differences by income group. There is a statistically significant difference in energy reduction between the two lowest income groups and the highest income group in terms of energy intake. Also, statistically significant differences are observed in reductions in sugar, fat and saturates between the two lowest income groups and the highest income group.

For lifestage group, Table 5 shows that the range of net reduction in energy is from around 408.2 kcal (C.I. −575, −242) (middle family) to approximately 724.3 kcal (C.I. −787, −661) (45+ no children). The decrease in sugar ranges from about 55.1 g (C.I. −73, −37) (middle family) to around 75.2 g (C.I. −82, −69) (45+ no children). The limits for fats [approximately 12.2 g (C.I. −21, −3) to 25.4 g (C.I. −29, −22)] are found amongst the middle family and 45+ no children groups, whilst for saturated fats [about 7.5 g (C.I. −12, −3) to 13.7 g (C.I. −17, −10)] are for middle family and pre-family. For sodium there are few differences as in the case by income group. There is a statistically significant difference in energy reduction between the 45+ lifestage group and the young, middle and older family groups. In terms of nutrients, there are statistically significant differences in reductions in fat, saturates and sodium between the 45+ lifestage group and the young and middle family groups.

Table 6 provides the simulation results of the substitution from discretionary food toward non-discretionary food and drink (the details are in Table A4). This is measured in terms of energy and nutrients from the purchases of other food and drink categories. The change in promotions generate a positive substitution effect increasing the share of non-discretionary products and compensating partially the reductions in energy, sugar and fat coming from the reduction of discretionary products. Based on the details presented in Table A4, almost all the categories show increases in energy and nutrients except in the case of ready meals, which shows a slight decrease. The highest increases in terms of energy are produced by fats and eggs (about 120.9 kcal.), which also shows the highest increases in fats (about 13.0 g.) and saturates (about 5.2 g.). The highest increases in total sugar come from fruit (about 4.6 g.) and vegetables (about 3.3 g.).

**Table 6:** Net results of the simulation on other non-discretionary food and drinks (Changes are in per capita per week terms).

		**Net impact**		
	**Total**	**Mean** **standard** **error**	**Total confidence interval lower**	**Total** **confidence** **interval** **upper**
**All the sample (9,737 observations)**				
Δ in share	0.038	0.000	0.038	0.039
Δ in expenditure (£)	1.056	0.013	1.031	1.081
Δ in quantity (Kg)	0.214	0.005	0.204	0.224
Δ in energy (kcal)	309.942	8.474	293.332	326.552
Δ in protein(g)	7.610	0.294	7.034	8.187
Δ in carbohydrate(g)	17.100	0.881	15.374	18.827
Δ in sugar(g)	10.657	0.428	9.819	11.496
Δ in fat(g)	18.422	0.688	17.072	19.771
Δ in saturates(g)	7.566	0.289	7.000	8.133
Δ in fiber(g)	2.609	0.100	2.412	2.806
Δ in sodium(g)	0.255	0.011	0.234	0.277

*Based on Table A4 in the Annex*.

## Discussion

The analyses suggested that a policy to restrict all price promotions of discretionary foods have the possibility to result in a net change of about −651 kcal (C.I. −695, −608) per capita per week (i.e., about −86.7 kcal per capita per day or approximately 4.4 per cent of a daily diet of 2,000 kcal) taking account of substitution of different items within food category and between food categories). This indicates that the measure due to the reduction in calories, sugar, saturated fats and salt has the potential to reduce obesity related deaths such as cardiovascular deaths.

All the nutritional categories showed similar results (calories, sugar, fat, salt), which indicates that the impact of restricting/banning promotions could be positive in terms of reductions of the purchase/consumption of foods high in fat, sugar and salt. It should be noted that the reduction in nutrients observed on the discretionary foods was only partially compensated by the increase in quantities in non-discretionary food and drinks (i.e., other food and drinks).

With regard to energy and nutrients from the purchases of other food and drink categories, almost all the categories show increases in energy and nutrients except in the case of ready meals, which shows a slight decrease. The highest increases in terms of energy are produced by fats and eggs (approximately 120.9 kcal.), which also shows the highest increases in fats (about 13 g.) and saturates (about 5.2 g.). The highest increases in total sugar come from fruit (around 4.6 g.) and vegetables (around 3.3 g.).

Given that no country or jurisdiction has restricted or banned the promotion of discretionary foods, it is difficult to compare our results with findings from previous studies [an exception is (9), although they only analyse promotions on soft drinks]. However, there is a large literature on the positive effect of price promotions on the purchases of discretionary foods, and there is consistent evidence that promotions are effective in increasing sales (i.e., the effect of applying promotions, not eliminating them). For example, Nakamura et al. (21) found that after controlling for reference price, price discount rate, and brand-specific effects, the increase in sales associated with price promotions was larger in less-healthy than healthier food categories. They argued that since less-healthy products (e.g., confectionary products) were often less perishable than healthier products (e.g., fruits and vegetables), they were more stockpiled as a result of price promotions. Similar findings were shown by Watt et al. (23), who recommended that policies removing or restricting the use of price promotions across the food sector needs to be evaluated for consumption and health effects. A more recent systematic review (24, 25) assessed the prevalence of healthy and unhealthy food and beverage price promotions and their influence on shopper purchasing behavior and found that price promotions were more common for unhealthy foods and beverages and that a greater proportion of price-promoted purchases were for unhealthy compared with healthy products. They thus suggest that policies aimed at reducing the prevalence and/or influence of price promotions on unhealthy foods and beverages might shift consumer purchasing away from unhealthy products.

## Final Remarks

The results reported in this paper were derived from data collected within Scotland. The strength of the study is that the estimation is based on a large sample for Scotland that shows variability in prices, expenditure and purchases under promotion. This is the only dataset that provides information about whether food and drink purchases were made under promotion. Standard errors and 95 per cent confidence intervals of the impact estimates indicate that the results are robust for all the studied groups.

The study considers all price promotions, some of which are likely to be retained in any future policy (e.g., temporary price reductions). This suggests that the modeled impacts may be best viewed as an upper bound on the overall actual impacts that would follow from an introduction of promotion restrictions for discretionary foods.

An important question relates to the transferability of study results from one country to another. There are many factors that can influence transferability, and these include the size and type of promotions, the characteristics of the study populations, differences in access to supermarkets and other retail outlets, between country differences in taxation, pricing, and differential regulation of food and drinks, including the relative availability of “unhealthy” and “healthy” food and drinks within supermarkets and other outlets. More generally, is has been noted that it is important to consider enabling policies, or the system-wide policy context where interventions are designed and delivered factors (25). Therefore, further studies using datasets beyond those limited to Scotland would be a welcome addition to the evidence base on the impact of promotion restrictions.

A limitation of the analysis is that it was only possible to include food bought for consumption in the home. According to the most recent information from Defra's Family Food 2017/18 (16), household consumption accounted for about 1,737 kcal and out of home consumption 202 kcal (approximately 10.3% in the UK and 10.4% in Scotland). It is unclear how restriction of promotions of discretionary foods bought in supermarkets and other retail outlets would impact on purchases of out of home foods. Further data analysis of out of home purchases would be required to assess these impacts.

In conclusion, the results indicate that restricting the advertising of promotions has the potential to reduce the number of calories, sugar, saturated fats and sodium (even when considering the substitution effects) for most food groups. However, it should be noted that the results are aggregated across all price promotions, some of which are likely to be retained in any future policy (e.g., temporary price reductions). This suggests that the modeled impacts may be best viewed as an upper bound on the overall actual impacts that would follow from an introduction of promotion restrictions for discretionary foods.

## Data Availability Statement

The data analyzed in this study is subject to the following licenses/restrictions: The Kantar Worldpanel dataset is proprietorial data and by contract we cannot disclose them. Nevertheless the Supplementary Tables contain a detailed analysis of the data. Requests to access these datasets should be directed to cesar.revoredo@sruc.ac.uk.

## Author Contributions

CRG planned the study, prepared the dataset, wrote the estimation, and simulation routines. PM wrote the manuscript. PN co-wrote the manuscript and prepare a literature review. FA estimated the first version of the models and compile part of the literature. WD estimated the price equations and re-estimated the models. All authors contributed to the article and approved the submitted version.

## Funding

This paper was funded by the Scottish Government *via* the research project Economic Modelling, Reducing Health Harms of Foods High in Fat, Sugar or Salt (reference JUN358629) and the Scottish Government Strategic Research Programme 2022–27, Project - Assessing the impact of dietary health interventions for driving long-term positive changes in diet and nutrition in Scotland.

## Conflict of Interest

The authors declare that the research was conducted in the absence of any commercial or financial relationships that could be construed as a potential conflict of interest.

## Publisher's Note

All claims expressed in this article are solely those of the authors and do not necessarily represent those of their affiliated organizations, or those of the publisher, the editors and the reviewers. Any product that may be evaluated in this article, or claim that may be made by its manufacturer, is not guaranteed or endorsed by the publisher.

## Footnotes

^2^The lifestage categories classify households by the age of the head of the household and the presence of dependent children in the household. The ‘pre-family' are from 16 years old to 34 years old without children (childless couples over the age of 35 years are automatically included in the empty nester); ‘young family' same age but with children; ‘middle family' are 35 years old to 44 years old with children; ‘older family' are those older than 44 years old and with children and 45+ without children is the remaining group (i.e., other dependents, empty nester and retired). All the classifications were provided by Kantar Worldpanel with the dataset.

^3^The model was estimated as a cross section. A preliminary version was estimated using fixed effects (i.e., as a panel data model); however, due to the similarities in the results, this paper uses the cross section to increase the number of observations as in the panel dataset those households with only one observation are eliminated from estimation. The full regressions for the different groups are presented in the Supplementary Tables S1–S11; the price equations are presented in Supplementary Table S12.
