# Supplementary material for: Expenditure and Nutritional Impact of Banning the Promotion of Foods High in Fat, Sugar and Salt in Scotland
Source: Front Nutr. 2022 Jun 29;9:874018. doi: 10.3389/fnut.2022.874018 (PMC9277539; doi:10.3389/fnut.2022.874018)
Supplement: Supplementary file 1 [file Table_1.docx]

**Annex**

Table A1 – Results of the simulation on discretionary foods

(Changes are in per capita per week terms)

| **Group** | **Category** | | | | | | | | |  |
| --- | --- | --- | --- | --- | --- | --- | --- | --- | --- | --- |
|  | **Discretionary foods** | | | | | | |  | **Other** | **Total** |
|  | **Take** | **Biscuits** | **Take** | **Cakes** | **Total** | **Take** | **Edible** | **Total** | **foods** |  |
|  | **home** |  | **home** | **pastries** | **puddings** | **home** | **ices and** |  | **and** |  |
|  | **confectionery** |  | **savouries** | **and sugar** | **and** | **sugary** | **ice cream** |  | **drinks** |  |
|  |  |  |  | **morning** | **desserts** | **drinks** |  |  |  |  |
|  |  |  |  | **goods** |  |  |  |  |  |  |
|  |  |  |  |  |  |  |  |  |  |  |
| **All the sample** | |  |  |  |  |  |  |  |  |  |
| Δ in share | -0.009 | -0.007 | -0.006 | -0.005 | -0.003 | -0.007 | -0.004 | -0.041 | 0.038 | -0.003 |
| Δ in expenditure (£) | -0.257 | -0.191 | -0.155 | -0.131 | -0.088 | -0.199 | -0.113 | -1.135 | 1.056 | -0.079 |
| Δ in quantity (Kg) | -0.081 | -0.033 | -0.020 | -0.094 | -0.016 | -0.173 | -0.033 | -0.449 | 0.214 | -0.235 |
| Δ in energy (kcal) | -377.422 | -152.426 | -100.468 | -176.288 | -34.275 | -51.073 | -69.426 | -961.380 | 309.942 | -651.438 |
| Δ in protein(g) | -4.053 | -2.111 | -1.509 | -4.306 | -0.516 | -0.215 | -0.882 | -13.591 | 7.610 | -5.981 |
| Δ in carbohydrate(g) | -52.210 | -20.937 | -10.331 | -27.722 | -4.639 | -11.997 | -8.243 | -136.078 | 17.100 | -118.978 |
| Δ in sugar(g) | -43.053 | -9.804 | -0.853 | -9.242 | -3.290 | -11.336 | -6.922 | -84.500 | 10.657 | -73.843 |
| Δ in fat(g) | -17.093 | -6.461 | -5.737 | -5.642 | -1.483 | -0.071 | -3.604 | -40.091 | 18.422 | -21.669 |
| Δ in saturates(g) | -10.000 | -3.237 | -0.655 | -2.173 | -0.866 | -0.031 | -2.460 | -19.421 | 7.566 | -11.855 |
| Δ in fibre(g) | -1.293 | -1.187 | -0.827 | -1.617 | -0.147 | -0.115 | -0.189 | -5.374 | 2.609 | -2.765 |
| Δ in sodium(g) | -0.081 | -0.086 | -0.118 | -0.153 | -0.015 | -0.016 | -0.019 | -0.488 | 0.255 | -0.233 |
|  |  |  |  |  |  |  |  |  |  |  |

Table A2 – Results of the simulation on discretionary foods - by income classification

(Changes are in per capita per week terms)

| **Group** | **Category** | | | | | | | | |  |
| --- | --- | --- | --- | --- | --- | --- | --- | --- | --- | --- |
|  | **Discretionary foods** | | | | | | |  | **Other** | **Total** |
|  | **Take** | **Biscuits** | **Take** | **Cakes** | **Total** | **Take** | **Edible** | **Total** | **foods** |  |
|  | **home** |  | **home** | **pastries** | **puddings** | **home** | **ices and** |  | **and** |  |
|  | **confectionery** |  | **savouries** | **and sugar** | **and** | **sugary** | **ice cream** |  | **drinks** |  |
|  |  |  |  | **morning** | **desserts** | **drinks** |  |  |  |  |
|  |  |  |  | **goods** |  |  |  |  |  |  |
|  |  |  |  |  |  |  |  |  |  |  |
| **£0 - £29,999** |  |  |  |  |  |  |  |  |  |  |
| Δ in share | -0.011 | -0.007 | -0.005 | -0.005 | -0.003 | -0.008 | -0.004 | -0.044 | 0.042 | -0.002 |
| Δ in expenditure (£) | -0.315 | -0.202 | -0.131 | -0.143 | -0.097 | -0.234 | -0.118 | -1.240 | 1.181 | -0.059 |
| Δ in quantity (Kg) | -0.103 | -0.036 | -0.017 | -0.109 | -0.019 | -0.207 | -0.035 | -0.526 | 0.241 | -0.286 |
| Δ in energy (kcal) | -482.142 | -169.164 | -86.133 | -202.083 | -38.436 | -60.303 | -74.630 | -1112.891 | 369.531 | -743.360 |
| Δ in protein(g) | -5.123 | -2.306 | -1.285 | -4.945 | -0.587 | -0.295 | -0.940 | -15.481 | 9.591 | -5.890 |
| Δ in carbohydrate(g) | -67.175 | -23.233 | -8.821 | -31.705 | -5.195 | -14.116 | -8.907 | -159.152 | 20.277 | -138.875 |
| Δ in sugar(g) | -55.103 | -10.989 | -0.713 | -10.970 | -3.678 | -13.371 | -7.450 | -102.274 | 11.638 | -90.636 |
| Δ in fat(g) | -21.703 | -7.193 | -4.941 | -6.548 | -1.662 | -0.101 | -3.856 | -46.004 | 22.641 | -23.363 |
| Δ in saturates(g) | -12.679 | -3.635 | -0.567 | -2.520 | -0.971 | -0.044 | -2.642 | -23.059 | 9.199 | -13.860 |
| Δ in fibre(g) | -1.634 | -1.278 | -0.700 | -1.815 | -0.166 | -0.152 | -0.207 | -5.951 | 3.053 | -2.897 |
| Δ in sodium(g) | -0.105 | -0.094 | -0.101 | -0.172 | -0.017 | -0.021 | -0.020 | -0.529 | 0.321 | -0.208 |
| **£30,000 - £39,999** |  |  |  |  |  |  |  |  |  |  |
| Δ in share | -0.010 | -0.010 | -0.006 | -0.005 | -0.003 | -0.009 | -0.004 | -0.046 | 0.039 | -0.007 |
| Δ in expenditure (£) | -0.268 | -0.254 | -0.155 | -0.120 | -0.073 | -0.240 | -0.104 | -1.213 | 1.034 | -0.179 |
| Δ in quantity (Kg) | -0.084 | -0.043 | -0.020 | -0.084 | -0.013 | -0.209 | -0.030 | -0.482 | 0.188 | -0.294 |
| Δ in energy (kcal) | -393.502 | -199.249 | -100.161 | -158.580 | -28.300 | -64.562 | -63.106 | -1007.462 | 250.459 | -757.003 |
| Δ in protein(g) | -4.260 | -2.761 | -1.483 | -3.723 | -0.420 | -0.193 | -0.824 | -13.664 | 6.307 | -7.357 |
| Δ in carbohydrate(g) | -53.796 | -27.241 | -10.374 | -24.803 | -3.868 | -15.317 | -7.320 | -142.720 | 7.946 | -134.774 |
| Δ in sugar(g) | -45.027 | -12.770 | -0.807 | -8.351 | -2.749 | -14.304 | -6.200 | -90.208 | 6.699 | -83.509 |
| Δ in fat(g) | -17.955 | -8.504 | -5.696 | -5.152 | -1.210 | -0.064 | -3.341 | -41.922 | 15.791 | -26.132 |
| Δ in saturates(g) | -10.562 | -4.265 | -0.635 | -1.991 | -0.706 | -0.028 | -2.280 | -20.467 | 6.197 | -14.271 |
| Δ in fibre(g) | -1.346 | -1.586 | -0.833 | -1.445 | -0.121 | -0.115 | -0.160 | -5.606 | 1.789 | -3.817 |
| Δ in sodium(g) | -0.082 | -0.113 | -0.119 | -0.136 | -0.012 | -0.014 | -0.018 | -0.494 | 0.198 | -0.296 |
|  |  |  |  |  |  |  |  |  |  |  |
|  |  |  |  |  |  |  |  |  |  |  |

Table A2 – Results of the simulation on discretionary foods - by income classification (cont.)

(Changes are in per capita per week terms)

| **Group** | **Category** | | | | | | | | |  |
| --- | --- | --- | --- | --- | --- | --- | --- | --- | --- | --- |
|  | **Discretionary foods** | | | | | | |  | **Other** | **Total** |
|  | **Take** | **Biscuits** | **Take** | **Cakes** | **Total** | **Take** | **Edible** | **Total** | **foods** |  |
|  | **home** |  | **home** | **pastries** | **puddings** | **home** | **ices and** |  | **and** |  |
|  | **confectionery** |  | **savouries** | **and sugar** | **and** | **sugary** | **ice cream** |  | **drinks** |  |
|  |  |  |  | **morning** | **desserts** | **drinks** |  |  |  |  |
|  |  |  |  | **goods** |  |  |  |  |  |  |
|  |  |  |  |  |  |  |  |  |  |  |
| **£40,000 - £49,999** |  |  |  |  |  |  |  |  |  |  |
| Δ in share | -0.008 | -0.005 | -0.008 | -0.002 | -0.003 | -0.006 | -0.005 | -0.037 | 0.031 | -0.006 |
| Δ in expenditure (£) | -0.206 | -0.139 | -0.198 | -0.065 | -0.070 | -0.160 | -0.136 | -0.974 | 0.814 | -0.160 |
| Δ in quantity (Kg) | -0.061 | -0.023 | -0.025 | -0.041 | -0.012 | -0.133 | -0.036 | -0.331 | 0.168 | -0.163 |
| Δ in energy (kcal) | -288.759 | -104.227 | -125.528 | -77.635 | -25.991 | -40.704 | -77.045 | -739.890 | 248.300 | -491.589 |
| Δ in protein(g) | -3.134 | -1.511 | -1.941 | -2.027 | -0.385 | -0.134 | -0.969 | -10.101 | 7.829 | -2.271 |
| Δ in carbohydrate(g) | -39.751 | -14.492 | -12.940 | -12.406 | -3.567 | -9.521 | -9.165 | -101.843 | 4.281 | -97.562 |
| Δ in sugar(g) | -33.148 | -6.518 | -1.120 | -3.540 | -2.529 | -8.979 | -7.749 | -63.582 | 5.809 | -57.773 |
| Δ in fat(g) | -13.103 | -4.289 | -7.118 | -2.346 | -1.103 | -0.040 | -4.000 | -31.999 | 18.076 | -13.923 |
| Δ in saturates(g) | -7.633 | -2.080 | -0.831 | -0.906 | -0.643 | -0.016 | -2.730 | -14.838 | 8.327 | -6.511 |
| Δ in fibre(g) | -0.986 | -0.908 | -1.069 | -0.754 | -0.113 | -0.077 | -0.196 | -4.103 | 1.077 | -3.026 |
| Δ in sodium(g) | -0.061 | -0.062 | -0.149 | -0.072 | -0.011 | -0.010 | -0.020 | -0.385 | 0.231 | -0.154 |
| **£50,000 - £59,999** |  |  |  |  |  |  |  |  |  |  |
| Δ in share | -0.013 | -0.008 | -0.008 | -0.009 | -0.002 | -0.011 | -0.004 | -0.055 | 0.046 | -0.009 |
| Δ in expenditure (£) | -0.339 | -0.205 | -0.197 | -0.221 | -0.051 | -0.290 | -0.107 | -1.410 | 1.170 | -0.240 |
| Δ in quantity (Kg) | -0.102 | -0.032 | -0.024 | -0.143 | -0.009 | -0.251 | -0.029 | -0.590 | 0.305 | -0.285 |
| Δ in energy (kcal) | -478.167 | -150.703 | -123.827 | -270.593 | -19.393 | -72.475 | -60.457 | -1175.616 | 465.699 | -709.917 |
| Δ in protein(g) | -5.190 | -2.109 | -1.841 | -6.522 | -0.287 | -0.232 | -0.763 | -16.943 | 16.517 | -0.426 |
| Δ in carbohydrate(g) | -66.096 | -20.839 | -13.041 | -42.631 | -2.588 | -17.240 | -7.265 | -169.701 | 49.530 | -120.170 |
| Δ in sugar(g) | -54.613 | -9.622 | -1.248 | -13.163 | -1.881 | -16.350 | -6.130 | -103.006 | 17.362 | -85.644 |
| Δ in fat(g) | -21.576 | -6.333 | -6.934 | -8.343 | -0.863 | -0.063 | -3.100 | -47.212 | 21.171 | -26.041 |
| Δ in saturates(g) | -12.615 | -3.144 | -0.782 | -3.194 | -0.500 | -0.024 | -2.077 | -22.334 | 9.599 | -12.735 |
| Δ in fibre(g) | -1.627 | -1.150 | -1.040 | -2.499 | -0.081 | -0.120 | -0.179 | -6.696 | 5.957 | -0.738 |
| Δ in sodium(g) | -0.105 | -0.088 | -0.147 | -0.250 | -0.008 | -0.016 | -0.017 | -0.631 | 0.605 | -0.026 |
|  |  |  |  |  |  |  |  |  |  |  |
|  |  |  |  |  |  |  |  |  |  |  |

Table A2 – Results of the simulation on discretionary foods - by income classification (cont.)

(Changes are in per capita per week terms)

| **Group** | **Category** | | | | | | | | |  |
| --- | --- | --- | --- | --- | --- | --- | --- | --- | --- | --- |
|  | **Discretionary foods** | | | | | | |  | **Other** | **Total** |
|  | **Take** | **Biscuits** | **Take** | **Cakes** | **Total** | **Take** | **Edible** | **Total** | **foods** |  |
|  | **home** |  | **home** | **pastries** | **puddings** | **home** | **ices and** |  | **and** |  |
|  | **confectionery** |  | **savouries** | **and sugar** | **and** | **sugary** | **ice cream** |  | **drinks** |  |
|  |  |  |  | **morning** | **desserts** | **drinks** |  |  |  |  |
|  |  |  |  | **goods** |  |  |  |  |  |  |
|  |  |  |  |  |  |  |  |  |  |  |
| **£60,000 - over** |  |  |  |  |  |  |  |  |  |  |
| Δ in share | -0.008 | -0.005 | -0.009 | -0.007 | -0.003 | -0.006 | -0.002 | -0.039 | 0.040 | 0.003 |
| Δ in expenditure (£) | -0.213 | -0.130 | -0.227 | -0.173 | -0.069 | -0.154 | -0.045 | -1.010 | 1.033 | 0.070 |
| Δ in quantity (Kg) | -0.054 | -0.019 | -0.027 | -0.110 | -0.012 | -0.122 | -0.012 | -0.355 | 0.261 | -0.093 |
| Δ in energy (kcal) | -255.254 | -89.665 | -138.831 | -207.492 | -26.813 | -35.415 | -25.694 | -779.164 | 388.119 | -358.963 |
| Δ in protein(g) | -2.959 | -1.345 | -2.217 | -4.805 | -0.372 | -0.096 | -0.345 | -12.139 | 6.854 | -3.128 |
| Δ in carbohydrate(g) | -33.510 | -12.100 | -14.047 | -32.674 | -3.536 | -8.392 | -3.026 | -107.285 | 29.814 | -76.464 |
| Δ in sugar(g) | -27.578 | -5.507 | -1.293 | -10.414 | -2.476 | -7.985 | -2.542 | -57.796 | 18.805 | -39.696 |
| Δ in fat(g) | -12.263 | -3.846 | -7.968 | -6.575 | -1.217 | -0.024 | -1.341 | -33.235 | 24.860 | -5.894 |
| Δ in saturates(g) | -7.251 | -1.851 | -0.903 | -2.547 | -0.711 | -0.007 | -0.898 | -14.168 | 10.603 | -2.979 |
| Δ in fibre(g) | -1.027 | -0.737 | -1.169 | -2.116 | -0.113 | -0.070 | -0.069 | -5.301 | 4.367 | -1.143 |
| Δ in sodium(g) | -0.051 | -0.052 | -0.166 | -0.183 | -0.011 | -0.012 | -0.007 | -0.482 | 0.237 | -0.154 |
|  |  |  |  |  |  |  |  |  |  |  |
|  |  |  |  |  |  |  |  |  |  |  |

Table A3 – Results of the simulation on discretionary foods - by lifestage classification

(Changes are in per capita per week terms)

| **Group** | **Category** | | | | | | | | |  |
| --- | --- | --- | --- | --- | --- | --- | --- | --- | --- | --- |
|  | **Discretionary foods** | | | | | | |  | **Other** | **Total** |
|  | **Take** | **Biscuits** | **Take** | **Cakes** | **Total** | **Take** | **Edible** | **Total** | **foods** |  |
|  | **home** |  | **home** | **pastries** | **puddings** | **home** | **ices and** |  | **and** |  |
|  | **confectionery** |  | **savouries** | **and sugar** | **and** | **sugary** | **ice cream** |  | **drinks** |  |
|  |  |  |  | **morning** | **desserts** | **drinks** |  |  |  |  |
|  |  |  |  | **goods** |  |  |  |  |  |  |
|  |  |  |  |  |  |  |  |  |  |  |
| **Pre-family** |  |  |  |  |  |  |  |  |  |  |
| Δ in share | -0.012 | -0.008 | -0.007 | -0.004 | -0.003 | -0.009 | -0.003 | -0.046 | 0.048 | 0.002 |
| Δ in expenditure (£) | -0.341 | -0.220 | -0.203 | -0.111 | -0.081 | -0.268 | -0.092 | -1.317 | 1.363 | 0.047 |
| Δ in quantity (Kg) | -0.108 | -0.035 | -0.025 | -0.078 | -0.014 | -0.221 | -0.024 | -0.504 | 0.271 | -0.233 |
| Δ in energy (kcal) | -498.142 | -161.790 | -125.124 | -146.025 | -30.527 | -71.143 | -50.812 | -1083.563 | 401.590 | -681.973 |
| Δ in protein(g) | -5.582 | -2.354 | -1.917 | -3.434 | -0.452 | -0.263 | -0.657 | -14.660 | 16.005 | 1.345 |
| Δ in carbohydrate(g) | -68.853 | -22.150 | -13.150 | -23.059 | -4.063 | -16.748 | -6.003 | -154.026 | 29.838 | -124.188 |
| Δ in sugar(g) | -54.858 | -10.264 | -1.130 | -7.004 | -2.943 | -15.817 | -5.011 | -97.028 | 14.980 | -82.047 |
| Δ in fat(g) | -22.899 | -6.827 | -6.999 | -4.508 | -1.360 | -0.093 | -2.651 | -45.336 | 20.314 | -25.022 |
| Δ in saturates(g) | -13.465 | -3.287 | -0.783 | -1.722 | -0.793 | -0.039 | -1.779 | -21.867 | 8.118 | -13.748 |
| Δ in fibre(g) | -1.704 | -1.365 | -1.079 | -1.519 | -0.132 | -0.116 | -0.132 | -6.047 | 3.574 | -2.472 |
| Δ in sodium(g) | -0.101 | -0.094 | -0.151 | -0.132 | -0.013 | -0.015 | -0.014 | -0.520 | 0.487 | -0.032 |
| **Young family** |  |  |  |  |  |  |  |  |  |  |
| Δ in share | -0.015 | -0.009 | -0.010 | -0.006 | -0.003 | -0.007 | -0.003 | -0.053 | 0.050 | -0.004 |
| Δ in expenditure (£) | -0.272 | -0.162 | -0.178 | -0.106 | -0.051 | -0.126 | -0.062 | -0.957 | 0.888 | -0.069 |
| Δ in quantity (Kg) | -0.091 | -0.027 | -0.023 | -0.063 | -0.010 | -0.108 | -0.017 | -0.340 | 0.227 | -0.113 |
| Δ in energy (kcal) | -431.542 | -123.026 | -118.178 | -120.668 | -21.339 | -35.197 | -34.940 | -884.890 | 373.499 | -511.391 |
| Δ in protein(g) | -4.756 | -1.699 | -1.572 | -2.796 | -0.333 | -0.143 | -0.437 | -11.736 | 11.236 | -0.500 |
| Δ in carbohydrate(g) | -59.157 | -17.281 | -12.860 | -18.886 | -2.992 | -8.292 | -4.298 | -123.765 | 27.299 | -96.466 |
| Δ in sugar(g) | -49.764 | -8.114 | -1.017 | -5.759 | -2.225 | -7.943 | -3.534 | -78.356 | 12.750 | -65.606 |
| Δ in fat(g) | -19.556 | -5.051 | -6.533 | -3.786 | -0.884 | -0.052 | -1.749 | -37.610 | 21.788 | -15.822 |
| Δ in saturates(g) | -11.432 | -2.463 | -0.716 | -1.474 | -0.510 | -0.021 | -1.187 | -17.804 | 9.195 | -8.609 |
| Δ in fibre(g) | -1.435 | -0.959 | -0.916 | -1.115 | -0.080 | -0.067 | -0.101 | -4.673 | 3.338 | -1.335 |
| Δ in sodium(g) | -0.090 | -0.069 | -0.148 | -0.114 | -0.009 | -0.007 | -0.009 | -0.448 | 0.324 | -0.124 |
|  |  |  |  |  |  |  |  |  |  |  |

Table A3 – Results of the simulation on discretionary foods - by lifestage classification (cont.)

(Changes are in per capita per week terms)

| **Group** | **Category** | | | | | | | | |  |
| --- | --- | --- | --- | --- | --- | --- | --- | --- | --- | --- |
|  | **Discretionary foods** | | | | | | |  | **Other** | **Total** |
|  | **Take** | **Biscuits** | **Take** | **Cakes** | **Total** | **Take** | **Edible** | **Total** | **foods** |  |
|  | **home** |  | **home** | **pastries** | **puddings** | **home** | **ices and** |  | **and** |  |
|  | **confectionery** |  | **savouries** | **and sugar** | **and** | **sugary** | **ice cream** |  | **drinks** |  |
|  |  |  |  | **morning** | **desserts** | **drinks** |  |  |  |  |
|  |  |  |  | **goods** |  |  |  |  |  |  |
|  |  |  |  |  |  |  |  |  |  |  |
| **Middle family** |  |  |  |  |  |  |  |  |  |  |
| Δ in share | -0.008 | -0.005 | -0.005 | -0.005 | -0.003 | -0.007 | -0.005 | -0.038 | 0.025 | -0.013 |
| Δ in expenditure (£) | -0.163 | -0.108 | -0.107 | -0.100 | -0.059 | -0.132 | -0.091 | -0.759 | 0.492 | -0.266 |
| Δ in quantity (Kg) | -0.061 | -0.018 | -0.014 | -0.063 | -0.011 | -0.120 | -0.025 | -0.312 | 0.137 | -0.176 |
| Δ in energy (kcal) | -289.425 | -82.718 | -70.840 | -118.477 | -25.673 | -33.854 | -52.801 | -673.787 | 265.562 | -408.226 |
| Δ in protein(g) | -3.168 | -1.158 | -1.004 | -2.823 | -0.388 | -0.141 | -0.676 | -9.358 | 12.926 | 3.568 |
| Δ in carbohydrate(g) | -39.870 | -11.471 | -7.521 | -18.631 | -3.434 | -7.991 | -6.424 | -95.343 | 21.298 | -74.044 |
| Δ in sugar(g) | -33.620 | -5.603 | -0.638 | -5.687 | -2.493 | -7.592 | -5.349 | -60.982 | 5.927 | -55.055 |
| Δ in fat(g) | -13.073 | -3.453 | -3.970 | -3.728 | -1.133 | -0.044 | -2.667 | -28.068 | 15.824 | -12.245 |
| Δ in saturates(g) | -7.613 | -1.703 | -0.440 | -1.444 | -0.633 | -0.019 | -1.830 | -13.681 | 6.188 | -7.493 |
| Δ in fibre(g) | -0.934 | -0.650 | -0.579 | -1.091 | -0.110 | -0.074 | -0.149 | -3.587 | 2.505 | -1.082 |
| Δ in sodium(g) | -0.062 | -0.045 | -0.086 | -0.111 | -0.011 | -0.008 | -0.015 | -0.338 | 0.396 | 0.058 |
| **Older family** |  |  |  |  |  |  |  |  |  |  |
| Δ in share | -0.006 | -0.007 | -0.003 | -0.005 | -0.006 | -0.009 | -0.006 | -0.041 | 0.034 | -0.007 |
| Δ in expenditure (£) | -0.121 | -0.134 | -0.055 | -0.096 | -0.111 | -0.182 | -0.113 | -0.810 | 0.670 | -0.141 |
| Δ in quantity (Kg) | -0.044 | -0.023 | -0.007 | -0.064 | -0.021 | -0.167 | -0.032 | -0.358 | 0.109 | -0.249 |
| Δ in energy (kcal) | -208.261 | -106.102 | -37.122 | -121.249 | -44.489 | -49.496 | -67.067 | -633.786 | 165.036 | -468.750 |
| Δ in protein(g) | -2.285 | -1.452 | -0.546 | -2.835 | -0.709 | -0.198 | -0.834 | -8.860 | 5.394 | -3.466 |
| Δ in carbohydrate(g) | -28.845 | -14.787 | -3.867 | -18.946 | -5.974 | -11.734 | -8.186 | -92.339 | 8.026 | -84.313 |
| Δ in sugar(g) | -23.887 | -7.073 | -0.327 | -5.976 | -4.265 | -11.137 | -6.852 | -59.518 | 3.293 | -56.225 |
| Δ in fat(g) | -9.403 | -4.409 | -2.105 | -3.857 | -1.914 | -0.073 | -3.390 | -25.152 | 8.437 | -16.715 |
| Δ in saturates(g) | -5.462 | -2.182 | -0.234 | -1.459 | -1.103 | -0.036 | -2.323 | -12.799 | 2.322 | -10.476 |
| Δ in fibre(g) | -0.661 | -0.826 | -0.306 | -1.148 | -0.182 | -0.095 | -0.174 | -3.391 | 1.391 | -2.000 |
| Δ in sodium(g) | -0.045 | -0.058 | -0.045 | -0.108 | -0.020 | -0.010 | -0.017 | -0.305 | 0.189 | -0.116 |
|  |  |  |  |  |  |  |  |  |  |  |
|  |  |  |  |  |  |  |  |  |  |  |

Table A3 – Results of the simulation on discretionary foods - by lifestage classification (cont.)

(Changes are in per capita per week terms)

| **Group** | **Category** | | | | | | | | |  |
| --- | --- | --- | --- | --- | --- | --- | --- | --- | --- | --- |
|  | **Discretionary foods** | | | | | | |  | **Other** | **Total** |
|  | **Take** | **Biscuits** | **Take** | **Cakes** | **Total** | **Take** | **Edible** | **Total** | **foods** |  |
|  | **home** |  | **home** | **pastries** | **puddings** | **home** | **ices and** |  | **and** |  |
|  | **confectionery** |  | **savouries** | **and sugar** | **and** | **sugary** | **ice cream** |  | **drinks** |  |
|  |  |  |  | **morning** | **desserts** | **drinks** |  |  |  |  |
|  |  |  |  | **goods** |  |  |  |  |  |  |
|  |  |  |  |  |  |  |  |  |  |  |
| **45+ no children** |  |  |  |  |  |  |  |  |  |  |
| Δ in share | -0.008 | -0.006 | -0.004 | -0.005 | -0.003 | -0.006 | -0.004 | -0.036 | 0.030 | -0.006 |
| Δ in expenditure (£) | -0.244 | -0.197 | -0.119 | -0.152 | -0.110 | -0.196 | -0.134 | -1.151 | 0.959 | -0.192 |
| Δ in quantity (Kg) | -0.072 | -0.035 | -0.015 | -0.118 | -0.020 | -0.169 | -0.041 | -0.471 | 0.185 | -0.285 |
| Δ in energy (kcal) | -337.500 | -163.804 | -76.688 | -219.427 | -41.796 | -48.002 | -86.848 | -974.065 | 249.670 | -724.394 |
| Δ in protein(g) | -3.550 | -2.245 | -1.199 | -5.496 | -0.625 | -0.214 | -1.105 | -14.434 | 3.439 | -10.995 |
| Δ in carbohydrate(g) | -46.790 | -22.352 | -7.631 | -34.534 | -5.661 | -11.225 | -10.181 | -138.374 | 12.897 | -125.477 |
| Δ in sugar(g) | -38.590 | -10.389 | -0.622 | -12.113 | -3.956 | -10.553 | -8.607 | -84.829 | 9.602 | -75.227 |
| Δ in fat(g) | -15.244 | -7.022 | -4.470 | -7.120 | -1.805 | -0.067 | -4.565 | -40.291 | 14.903 | -25.388 |
| Δ in saturates(g) | -8.925 | -3.575 | -0.525 | -2.745 | -1.062 | -0.029 | -3.125 | -19.986 | 6.508 | -13.478 |
| Δ in fibre(g) | -1.186 | -1.251 | -0.633 | -1.945 | -0.184 | -0.126 | -0.236 | -5.560 | 2.192 | -3.368 |
| Δ in sodium(g) | -0.074 | -0.093 | -0.087 | -0.183 | -0.018 | -0.020 | -0.023 | -0.497 | 0.142 | -0.355 |
|  |  |  |  |  |  |  |  |  |  |  |
|  |  |  |  |  |  |  |  |  |  |  |

Table A4 – Results of the policy simulation - other food and drinks

(Changes are in per capita per week terms)

|  | **Other food and drinks** | | | | | | | | | | | |
| --- | --- | --- | --- | --- | --- | --- | --- | --- | --- | --- | --- | --- |
|  | **Dairy** | **Meat** | **Fats** | **Fruit** | **Vegetables** | **Grains** | **Prepared** | **Sugar** | **Condiments** | **Low calorie** | **Alcoholic** | **Total** |
|  | **products** | **and** | **and** |  |  |  | **ready to** | **and** | **and** | **soft drinks** | **beverages** |  |
|  |  | **fish** | **eggs** |  |  |  | **eat foods** | **preserves** | **sauces** | **and** |  |  |
|  |  |  |  |  |  |  |  |  |  | **juices** |  |  |
|  |  |  |  |  |  |  |  |  |  |  |  |  |
|  |  |  |  |  |  |  |  |  |  |  |  |  |
| **All the sample** | | |  |  |  |  |  |  |  |  |  |  |
| Δ in share | 0.003 | 0.004 | 0.003 | 0.005 | 0.007 | 0.001 | -0.003 | 0.000 | 0.001 | 0.001 | 0.016 | 0.038 |
| Δ in expenditure (£) | 0.076 | 0.115 | 0.071 | 0.142 | 0.204 | 0.017 | -0.079 | 0.009 | 0.030 | 0.026 | 0.446 | 1.056 |
| Δ in quantity (Kg) | 0.020 | 0.014 | 0.025 | 0.037 | 0.079 | 0.006 | -0.014 | 0.002 | 0.003 | 0.003 | 0.039 | 0.214 |
| Δ in energy (kcal) | 34.621 | 26.090 | 120.926 | 30.970 | 48.163 | 16.050 | -24.382 | 5.298 | 3.750 | 1.897 | 46.557 | 309.942 |
| Δ in protein(g) | 1.929 | 2.893 | 0.796 | 0.550 | 1.860 | 0.485 | -1.151 | 0.071 | 0.073 | 0.063 | 0.042 | 7.610 |
| Δ in carbohydrate(g) | 1.204 | 0.449 | 0.167 | 4.960 | 7.642 | 2.801 | -2.259 | 0.801 | 0.428 | 0.309 | 0.599 | 17.100 |
| Δ in sugar(g) | 1.025 | 0.092 | 0.114 | 4.560 | 3.337 | 0.394 | -0.435 | 0.609 | 0.257 | 0.221 | 0.483 | 10.657 |
| Δ in fat(g) | 2.437 | 1.411 | 13.035 | 1.009 | 0.971 | 0.278 | -1.157 | 0.198 | 0.181 | 0.046 | 0.014 | 18.422 |
| Δ in saturates(g) | 1.553 | 0.531 | 5.188 | 0.186 | 0.194 | 0.080 | -0.332 | 0.082 | 0.040 | 0.035 | 0.006 | 7.566 |
| Δ in fibre(g) | 0.033 | 0.054 | 0.020 | 0.630 | 1.703 | 0.249 | -0.225 | 0.025 | 0.045 | 0.075 | 0.000 | 2.609 |
| Δ in sodium(g) | 0.051 | 0.064 | 0.071 | 0.007 | 0.039 | 0.016 | -0.053 | 0.004 | 0.052 | 0.002 | 0.003 | 0.255 |
|  |  |  |  |  |  |  |  |  |  |  |  |  |
|  |  |  |  |  |  |  |  |  |  |  |  |  |
