# Supplementary material for: Expenditure and Nutritional Impact of Banning the Promotion of Foods High in Fat, Sugar and Salt in Scotland
Source: Front Nutr. 2022 Jun 29;9:874018. doi: 10.3389/fnut.2022.874018 (PMC9277539; doi:10.3389/fnut.2022.874018)
Supplement: Supplementary file 2 [file Data_Sheet_1.pdf]

Supplementary Tables

Table S1 - Scotland - All the sample - All categories - Demand model results

Equations (1–5)

Variables	Take home confectionery			Biscuits			Take home savouries			Cakes, pastries and sugar morning goods			Total puddings and desserts		
	Coef.	St. Err.	Sig.	Coef.	St. Err.	Sig.	Coef.	St. Err.	Sig.	Coef.	St. Err.	Sig.	Coef.	St. Err.	Sig.
**Intercept**	0.032	0.002	*	0.017	0.004	*	0.040	0.004	*	0.031	0.002	*	0.015	0.002	*
**Prices**															
Take home confectionery	-0.006	0.001	*	-0.002	0.001	*	-0.004	0.001	*	0.000	0.001		-0.001	0.000	*
Biscuits	-0.002	0.001	*	0.000	0.006		0.012	0.005	*	-0.001	0.001		-0.006	0.003	*
Take home savouries	-0.004	0.001	*	0.012	0.005	*	-0.004	0.007		0.001	0.001		0.005	0.003	*
Cakes, pastries, and sugar morning goods	0.000	0.001		-0.001	0.001		0.001	0.001		-0.001	0.001		0.000	0.001	
Total puddings and desserts	-0.001	0.000	*	-0.006	0.003	*	0.005	0.003	*	0.000	0.001		-0.011	0.002	*
Take home sugary drinks	0.000	0.000		0.000	0.000		-0.001	0.000	*	0.000	0.000		0.000	0.000	
Edible ices and ice creams	-0.001	0.000		0.000	0.003		0.002	0.003		0.001	0.001	*	0.000	0.002	
Dairy products	0.002	0.001	*	-0.005	0.003	*	0.006	0.003	*	-0.002	0.002		-0.002	0.002	
Meat and fish	-0.006	0.002	*	0.011	0.007		-0.019	0.007	*	0.005	0.003		0.000	0.004	
Fats and eggs	0.000	0.001		-0.001	0.001		0.000	0.001		0.000	0.001		0.001	0.001	
Fruit	0.010	0.001	*	0.005	0.003		-0.011	0.003	*	0.000	0.002		-0.002	0.002	
Vegetables	0.003	0.001	*	-0.006	0.002	*	0.004	0.002	*	0.003	0.002	*	0.002	0.001	
Grains	-0.001	0.001	*	0.004	0.002	*	0.003	0.002		-0.001	0.001		-0.001	0.001	
Prepared ready to eat foods	-0.004	0.002	*	-0.020	0.007	*	0.015	0.007	*	-0.004	0.003		0.014	0.004	*
Sugar and preserves	0.000	0.000		0.008	0.002	*	-0.003	0.002		0.000	0.001		-0.002	0.001	
Condiments and sauces	-0.001	0.000	*	0.001	0.002		-0.004	0.002	*	0.000	0.001		0.000	0.001	
Low calorie, soft drinks and juices	-0.001	0.001		0.004	0.001	*	0.000	0.001		-0.003	0.001	*	0.002	0.001	*
Alcoholic beverages	0.009	0.002	*	-0.001	0.002		0.000	0.002		0.001	0.002		0.000	0.001	
Numeraire category	0.001	-0.017		-0.001	-0.052		-0.002	-0.053		0.000	-0.024		0.000	-0.031	
**Total expenditure**	0.002	0.001	*	-0.003	0.000	*	-0.006	0.000	*	-0.001	0.000	*	0.000	0.000	
**Promotions**															
Take home confectionery	0.006	0.000	*	0.000	0.000	*	0.000	0.000	*	0.001	0.000	*	0.000	0.000	*
Biscuits	0.000	0.000		0.004	0.000	*	0.000	0.000		0.001	0.000	*	0.000	0.000	
Take home savouries	0.000	0.000		0.000	0.000		0.002	0.000	*	0.000	0.000		0.000	0.000	
Cakes, pastries, and sugar morning goods	0.000	0.000	*	0.000	0.000	*	0.000	0.000	*	0.002	0.000	*	0.000	0.000	
Total puddings and desserts	0.001	0.000	*	0.000	0.000	*	0.000	0.000		0.000	0.000	*	0.002	0.000	*
Take home sugary drinks	0.000	0.000		0.000	0.000		0.000	0.000	*	0.000	0.000		0.000	0.000	*
Edible ices and ice creams	0.001	0.000	*	0.000	0.000		0.000	0.000		0.000	0.000		0.000	0.000	*
Dairy products	0.000	0.000		0.000	0.000	*	0.000	0.000		0.000	0.000		0.000	0.000	*
Meat and fish	-0.001	0.000	*	0.000	0.000	*	0.000	0.000		0.000	0.000		0.000	0.000	*
Fats and eggs	0.000	0.000	*	0.000	0.000	*	0.000	0.000	*	0.000	0.000		0.000	0.000	
Fruit	0.000	0.000	*	0.000	0.000	*	0.000	0.000	*	0.000	0.000	*	0.000	0.000	
Vegetables	-0.001	0.000	*	0.000	0.000	*	-0.001	0.000	*	-0.001	0.000	*	0.000	0.000	*
Grains	0.000	0.000	*	0.000	0.000		0.000	0.000		0.000	0.000		0.000	0.000	*
Prepared ready to eat foods	0.000	0.000		0.000	0.000	*	0.000	0.000	*	0.000	0.000		0.000	0.000	
Sugar and preserves	0.001	0.000	*	0.000	0.000		0.000	0.000	*	0.001	0.000	*	0.000	0.000	
Condiments and sauces	0.000	0.000		0.000	0.000	*	0.000	0.000	*	0.000	0.000		0.000	0.000	*
Low calorie, soft drinks and juices	0.000	0.000		0.000	0.000	*	0.000	0.000	*	0.000	0.000	*	0.000	0.000	*
Alcoholic beverages	-0.002	0.000	*	-0.001	0.000	*	-0.001	0.000	*	-0.001	0.000	*	0.000	0.000	*
Numeraire category	-0.002	0.000	*	-0.001	0.000	*	-0.001	0.000	*	-0.001	0.000	*	0.000	0.000	*
Log-Likelihood	431857.000														
Obs.	9914.000														

Equations (6–10)

Variables	Take home sugary drinks			Edible ices and ice cream			Dairy products			Meat and fish			Fats and eggs		
	Coef.	St. Err.	Sig.	Coef.	St. Err.	Sig.	Coef.	St. Err.	Sig.	Coef.	St. Err.	Sig.	Coef.	St. Err.	Sig.
**Intercept**	0.016	0.001	*	0.013	0.002	*	0.096	0.005	*	0.136	0.010	*	0.029	0.002	*
**Prices**															
Take home confectionery	0.000	0.000		-0.001	0.000		0.002	0.001	*	-0.006	0.002	*	0.000	0.001	
Biscuits	0.000	0.000		0.000	0.003		-0.005	0.003	*	0.011	0.007		-0.001	0.001	
Take home savouries	-0.001	0.000	*	0.002	0.003		0.006	0.003	*	-0.019	0.007	*	0.000	0.001	
Cakes, pastries, and sugar morning goods	0.000	0.000		0.001	0.001	*	-0.002	0.002		0.005	0.003		0.000	0.001	
Total puddings and desserts	0.000	0.000		0.000	0.002		-0.002	0.002		0.000	0.004		0.001	0.001	
Take home sugary drinks	0.000	0.000		0.000	0.000		-0.001	0.000	*	-0.001	0.000	*	0.000	0.000	*
Edible ices and ice creams	0.000	0.000		0.002	0.003		-0.004	0.002	*	-0.003	0.005		0.001	0.001	*
Dairy products	-0.001	0.000	*	-0.004	0.002	*	-0.019	0.006	*	0.009	0.008		0.001	0.002	
Meat and fish	-0.001	0.000	*	-0.003	0.005		0.009	0.008		0.001	0.022		0.001	0.003	
Fats and eggs	0.000	0.000	*	0.001	0.001	*	0.001	0.002		0.001	0.003		0.002	0.001	
Fruit	0.000	0.000		0.004	0.002	*	0.009	0.005	*	-0.009	0.009		-0.006	0.002	*
Vegetables	0.000	0.000		0.002	0.001		-0.008	0.003	*	-0.025	0.006	*	-0.005	0.001	*
Grains	-0.001	0.000	*	-0.003	0.001	*	-0.003	0.003		0.016	0.006	*	-0.004	0.001	*
Prepared ready to eat foods	0.000	0.000		-0.003	0.005		0.015	0.007	*	0.027	0.016		0.007	0.003	*
Sugar and preserves	0.000	0.000		-0.002	0.002		0.000	0.002		0.003	0.004		-0.001	0.001	
Condiments and sauces	0.000	0.000		0.001	0.002		0.000	0.002		0.007	0.004	*	0.000	0.001	
Low calorie, soft drinks and juices	0.000	0.000		0.002	0.001	*	0.010	0.002	*	-0.022	0.004	*	0.002	0.001	*
Alcoholic beverages	0.003	0.001	*	0.001	0.001		-0.005	0.003		0.007	0.006		0.003	0.001	*
Numeraire category	0.001	-0.004		0.000	-0.034		-0.003	-0.056		-0.002	-0.118		-0.001	-0.023	
**Total expenditure**	0.001	0.000	*	-0.001	0.000	*	-0.010	0.001	*	-0.007	0.001	*	-0.001	0.000	*
**Promotions**															
Take home confectionery	0.000	0.000	*	0.000	0.000	*	0.000	0.000		-0.001	0.000	*	0.000	0.000	*
Biscuits	0.000	0.000	*	0.000	0.000	*	0.000	0.000	*	-0.001	0.000	*	0.000	0.000	*
Take home savouries	0.000	0.000		0.000	0.000		0.000	0.000	*	0.001	0.000	*	0.000	0.000	*
Cakes, pastries, and sugar morning goods	0.000	0.000	*	0.000	0.000		0.001	0.000	*	0.000	0.000		0.000	0.000	
Total puddings and desserts	0.000	0.000	*	0.000	0.000	*	0.000	0.000	*	-0.001	0.000	*	0.000	0.000	*
Take home sugary drinks	0.003	0.000	*	0.000	0.000	*	-0.001	0.000	*	0.000	0.000		0.000	0.000	*
Edible ices and ice creams	0.000	0.000		0.003	0.000	*	-0.001	0.000	*	-0.001	0.000	*	0.000	0.000	*
Dairy products	0.000	0.000		0.000	0.000		0.002	0.000	*	-0.001	0.000	*	0.000	0.000	
Meat and fish	0.000	0.000		0.000	0.000	*	0.000	0.000	*	0.007	0.000	*	0.000	0.000	
Fats and eggs	0.000	0.000	*	0.000	0.000	*	0.000	0.000		0.002	0.000	*	0.002	0.000	*
Fruit	0.000	0.000	*	0.000	0.000		0.001	0.000	*	-0.001	0.000	*	0.000	0.000	*
Vegetables	0.000	0.000	*	0.000	0.000	*	0.000	0.000	*	0.001	0.000	*	0.000	0.000	*
Grains	0.000	0.000	*	0.000	0.000		0.001	0.000	*	-0.001	0.000	*	0.000	0.000	
Prepared ready to eat foods	0.000	0.000		0.000	0.000		0.000	0.000		-0.004	0.000	*	-0.001	0.000	*
Sugar and preserves	0.000	0.000		0.000	0.000		-0.001	0.000	*	0.000	0.001		0.000	0.000	
Condiments and sauces	0.000	0.000		0.000	0.000		0.000	0.000		0.000	0.000		0.000	0.000	*
Low calorie, soft drinks and juices	0.000	0.000	*	0.000	0.000		0.000	0.000	*	0.000	0.000	*	0.000	0.000	*
Alcoholic beverages	-0.001	0.000	*	0.000	0.000	*	-0.002	0.000	*	-0.001	0.000	*	0.000	0.000	*
Numeraire category	-0.001	0.000	*	-0.001	0.000	*	-0.001	0.000	*	-0.002	0.000	*	0.000	0.000	*
Log-Likelihood															
Obs.															

Equations (11–15)

Variables	Fruit			Vegetables			Grains			Prepared ready to eat foods			Sugar and preserves		
	Coef.	St. Err.	Sig.	Coef.	St. Err.	Sig.	Coef.	St. Err.	Sig.	Coef.	St. Err.	Sig.	Coef.	St. Err.	Sig.
**Intercept**	0.079	0.006	*	0.077	0.004	*	0.064	0.003	*	0.130	0.009	*	0.021	0.002	*
**Prices**															
Take home confectionery	0.010	0.001	*	0.003	0.001	*	-0.001	0.001	*	-0.004	0.002	*	0.000	0.000	
Biscuits	0.005	0.003		-0.006	0.002	*	0.004	0.002	*	-0.020	0.007	*	0.008	0.002	*
Take home savouries	-0.011	0.003	*	0.004	0.002	*	0.003	0.002		0.015	0.007	*	-0.003	0.002	
Cakes, pastries, and sugar morning goods	0.000	0.002		0.003	0.002	*	-0.001	0.001		-0.004	0.003		0.000	0.001	
Total puddings and desserts	-0.002	0.002		0.002	0.001		-0.001	0.001		0.014	0.004	*	-0.002	0.001	
Take home sugary drinks	0.000	0.000		0.000	0.000		-0.001	0.000	*	0.000	0.000		0.000	0.000	
Edible ices and ice creams	0.004	0.002	*	0.002	0.001		-0.003	0.001	*	-0.003	0.005		-0.002	0.002	
Dairy products	0.009	0.005	*	-0.008	0.003	*	-0.003	0.003		0.015	0.007	*	0.000	0.002	
Meat and fish	-0.009	0.009		-0.025	0.006	*	0.016	0.006	*	0.027	0.016		0.003	0.004	
Fats and eggs	-0.006	0.002	*	-0.005	0.001	*	-0.004	0.001	*	0.007	0.003	*	-0.001	0.001	
Fruit	0.000	0.007		0.000	0.004		-0.001	0.003		0.005	0.009		0.001	0.002	
Vegetables	0.000	0.004		-0.001	0.004		0.001	0.002		0.032	0.006	*	-0.003	0.001	*
Grains	-0.001	0.003		0.001	0.002		-0.002	0.003		0.001	0.005		-0.005	0.001	*
Prepared ready to eat foods	0.005	0.009		0.032	0.006	*	0.001	0.005		-0.096	0.021	*	0.010	0.004	*
Sugar and preserves	0.001	0.002		-0.003	0.001	*	-0.005	0.001	*	0.010	0.004	*	-0.009	0.002	*
Condiments and sauces	-0.003	0.002	*	0.002	0.001		0.005	0.001	*	-0.003	0.004		0.001	0.001	
Low calorie, soft drinks and juices	0.006	0.003	*	0.007	0.002	*	-0.003	0.001	*	-0.001	0.004		0.002	0.001	*
Alcoholic beverages	-0.009	0.004	*	-0.005	0.003	*	-0.002	0.002		0.009	0.005	*	0.000	0.001	
Numeraire category	0.000	-0.063		-0.002	-0.045		-0.004	-0.039		-0.003	-0.112		0.000	-0.028	
**Total expenditure**	0.001	0.001		-0.002	0.001	*	-0.013	0.000	*	-0.011	0.001	*	-0.002	0.000	*
**Promotions**															
Take home confectionery	-0.001	0.000	*	-0.001	0.000	*	0.000	0.000		0.000	0.000		0.000	0.000	
Biscuits	0.000	0.000		-0.001	0.000	*	0.000	0.000		0.000	0.000		0.000	0.000	*
Take home savouries	-0.001	0.000	*	0.000	0.000	*	0.000	0.000		0.000	0.000		0.000	0.000	
Cakes, pastries, and sugar morning goods	0.000	0.000		0.000	0.000		0.000	0.000		0.000	0.000		0.000	0.000	
Total puddings and desserts	0.000	0.000		-0.001	0.000	*	0.000	0.000		0.001	0.000	*	0.000	0.000	
Take home sugary drinks	-0.001	0.000	*	-0.001	0.000	*	0.000	0.000	*	0.001	0.000	*	0.000	0.000	*
Edible ices and ice creams	0.000	0.000		0.000	0.000	*	0.000	0.000	*	0.000	0.000		0.000	0.000	
Dairy products	0.000	0.000		0.000	0.000		0.001	0.000	*	-0.001	0.000	*	0.000	0.000	
Meat and fish	-0.001	0.000	*	0.000	0.000		0.000	0.000	*	-0.001	0.000	*	0.000	0.000	*
Fats and eggs	0.000	0.000	*	0.000	0.000		0.000	0.000		-0.001	0.000	*	0.000	0.000	
Fruit	0.006	0.000	*	0.001	0.000	*	0.000	0.000	*	0.000	0.000		0.000	0.000	*
Vegetables	0.002	0.000	*	0.005	0.000	*	0.000	0.000		-0.002	0.000	*	0.000	0.000	
Grains	0.000	0.000		0.000	0.000	*	0.002	0.000	*	0.001	0.000	*	0.000	0.000	*
Prepared ready to eat foods	-0.002	0.000	*	-0.002	0.000	*	0.000	0.000	*	0.011	0.000	*	0.000	0.000	*
Sugar and preserves	-0.001	0.000	*	-0.001	0.000	*	0.000	0.000		0.001	0.000	*	0.003	0.000	*
Condiments and sauces	0.000	0.000		0.001	0.000	*	0.001	0.000	*	0.001	0.000	*	0.000	0.000	*
Low calorie, soft drinks and juices	0.000	0.000	*	0.000	0.000	*	0.000	0.000	*	0.000	0.000		0.000	0.000	*
Alcoholic beverages	-0.002	0.000	*	-0.001	0.000	*	-0.002	0.000	*	-0.003	0.000	*	-0.001	0.000	*
Numeraire category	-0.002	0.000	*	-0.001	0.000	*	-0.001	0.000	*	-0.001	0.000	*	0.000	0.000	*
Log-Likelihood															
Obs.															

Equations (16–19)

Variables	Condiments and sauces			Low calorie soft drinks and juices			Alcoholic beverages			Numeraire category		
	Coef.	St. Err.	Sig.	Coef.	St. Err.	Sig.	Coef.	St. Err.	Sig.	Coef.	St. Err.	Sig.
**Intercept**	0.027	0.002	*	0.054	0.003	*	0.055	0.008	*	0.069	0.020	*
**Prices**												
Take home confectionery	-0.001	0.000	*	-0.001	0.001		0.009	0.002	*	0.001	0.005	
Biscuits	0.001	0.002		0.004	0.001	*	-0.001	0.002		-0.001	0.015	
Take home savouries	-0.004	0.002	*	0.000	0.001		0.000	0.002		-0.002	0.015	
Cakes, pastries, and sugar morning goods	0.000	0.001		-0.003	0.001	*	0.001	0.002		0.000	0.007	
Total puddings and desserts	0.000	0.001		0.002	0.001	*	0.000	0.001		0.000	0.009	
Take home sugary drinks	0.000	0.000		0.000	0.000		0.003	0.001	*	0.001	0.001	
Edible ices and ice creams	0.001	0.002		0.002	0.001	*	0.001	0.001		0.000	0.010	
Dairy products	0.000	0.002		0.010	0.002	*	-0.005	0.003		-0.003	0.016	
Meat and fish	0.007	0.004	*	-0.022	0.004	*	0.007	0.006		-0.002	0.035	
Fats and eggs	0.000	0.001		0.002	0.001	*	0.003	0.001	*	-0.001	0.007	
Fruit	-0.003	0.002	*	0.006	0.003	*	-0.009	0.004	*	0.000	0.018	
Vegetables	0.002	0.001		0.007	0.002	*	-0.005	0.003	*	-0.002	0.013	
Grains	0.005	0.001	*	-0.003	0.001	*	-0.002	0.002		-0.004	0.011	
Prepared ready to eat foods	-0.003	0.004		-0.001	0.004		0.009	0.005	*	-0.003	0.033	
Sugar and preserves	0.001	0.001		0.002	0.001	*	0.000	0.001		0.000	0.008	
Condiments and sauces	-0.004	0.002	*	-0.001	0.001		0.000	0.001		-0.001	0.008	
Low calorie, soft drinks and juices	-0.001	0.001		-0.001	0.002		-0.003	0.002		-0.001	0.008	
Alcoholic beverages	0.000	0.001		-0.003	0.002		-0.014	0.009		0.006	0.014	
Numeraire category	-0.001	-0.029		-0.001	-0.029		0.006	-0.048		0.012	0.225	
**Total expenditure**	-0.002	0.000	*	-0.002	0.000	*	0.026	0.002	*	0.031	0.003	*
**Promotions**												
Take home confectionery	0.000	0.000	*	0.000	0.000	*	-0.005	0.001	*	0.000	0.001	
Biscuits	0.000	0.000		0.000	0.000		-0.002	0.001	*	0.001	0.001	
Take home savouries	0.000	0.000		0.000	0.000		0.000	0.000		-0.001	0.001	*
Cakes, pastries, and sugar morning goods	0.000	0.000		0.000	0.000		-0.001	0.000	*	-0.001	0.001	
Total puddings and desserts	0.000	0.000	*	0.001	0.000	*	-0.003	0.001	*	0.000	0.001	
Take home sugary drinks	0.000	0.000		-0.001	0.000	*	0.000	0.000		0.000	0.000	
Edible ices and ice creams	0.000	0.000	*	0.000	0.000		-0.001	0.000	*	0.000	0.001	
Dairy products	0.000	0.000		0.000	0.000		-0.002	0.000	*	0.000	0.000	
Meat and fish	0.000	0.000		0.000	0.000	*	-0.001	0.001		-0.001	0.001	
Fats and eggs	0.000	0.000	*	0.000	0.000	*	0.000	0.000		-0.001	0.001	
Fruit	0.000	0.000	*	0.000	0.000	*	-0.002	0.000	*	-0.002	0.001	*
Vegetables	0.000	0.000		-0.001	0.000	*	-0.001	0.001		-0.002	0.001	*
Grains	0.000	0.000		0.000	0.000		-0.001	0.000	*	0.000	0.001	
Prepared ready to eat foods	0.000	0.000	*	0.000	0.000		-0.003	0.001	*	0.001	0.001	
Sugar and preserves	-0.001	0.000	*	0.001	0.000	*	-0.002	0.001	*	0.000	0.001	
Condiments and sauces	0.003	0.000	*	0.000	0.000	*	-0.003	0.001	*	-0.001	0.001	
Low calorie, soft drinks and juices	0.000	0.000		0.003	0.000	*	0.000	0.000	*	-0.001	0.000	
Alcoholic beverages	0.000	0.000	*	-0.001	0.000	*	0.022	0.000	*	-0.002	0.001	*
Numeraire category	0.000	0.000	*	0.000	0.000	*	-0.002	0.001	*	0.017	0.001	*
Log-Likelihood												
Obs.												

Note: '*' indicates statistically significant at 5 per cent.

Table S2 - Scotland - £0 - £29,999 income group - All categories - Demand model results

Variables	Equations														
	Take home confectionery			Biscuits			Take home savouries			Cakes, pastries and sugar morning goods			Total puddings and desserts		
	Coef.	St. Err.	Sig.	Coef.	St. Err.	Sig.	Coef.	St. Err.	Sig.	Coef.	St. Err.	Sig.	Coef.	St. Err.	Sig.
**Intercept**	0.040	0.008	*	0.004	0.010		0.045	0.010	*	0.041	0.010	*	0.026	0.006	*
**Prices**															
Take home confectionery	-0.003	0.003		-0.004	0.002	*	-0.007	0.002	*	0.002	0.002		0.001	0.001	
Biscuits	-0.004	0.002	*	0.004	0.005		0.008	0.004	*	-0.006	0.003	*	-0.005	0.002	*
Take home savouries	-0.007	0.002	*	0.008	0.004	*	-0.012	0.005	*	0.009	0.003	*	0.001	0.002	
Cakes, pastries, and sugar morning goods	0.002	0.002		-0.006	0.003	*	0.009	0.003	*	-0.007	0.004	*	0.000	0.002	
Total puddings and desserts	0.001	0.001		-0.005	0.002	*	0.001	0.002		0.000	0.002		-0.005	0.002	*
Take home sugary drinks	-0.004	0.002	*	0.001	0.003		0.006	0.003	*	0.004	0.003	*	0.001	0.002	
Edible ices and ice creams	-0.002	0.001		0.002	0.003		-0.001	0.003		-0.003	0.002		0.001	0.002	
Dairy products	0.006	0.003	*	-0.003	0.004		0.009	0.004	*	0.000	0.004		-0.001	0.003	
Meat and fish	-0.003	0.005		0.006	0.006		-0.015	0.006	*	0.001	0.006		-0.003	0.004	
Fats and eggs	0.003	0.002		-0.002	0.003		-0.004	0.003		0.004	0.002	*	0.003	0.002	*
Fruit	0.004	0.003		0.003	0.004		-0.006	0.004		0.001	0.004		-0.004	0.002	*
Vegetables	0.003	0.003		-0.003	0.004		-0.001	0.004		0.003	0.004		0.003	0.002	
Grains	-0.001	0.002		0.002	0.003		0.002	0.003		0.006	0.003	*	0.000	0.002	
Prepared ready to eat foods	0.004	0.005		-0.004	0.007		0.010	0.007		-0.009	0.007		0.010	0.004	*
Sugar and preserves	0.001	0.001		0.002	0.001	*	0.000	0.001		0.001	0.001		0.000	0.001	
Condiments and sauces	0.000	0.001		0.000	0.001		-0.002	0.001	*	-0.002	0.001		0.000	0.001	
Low calorie, soft drinks and juices	-0.004	0.002	*	0.001	0.002		0.003	0.002		-0.005	0.002	*	0.000	0.001	
Alcoholic beverages	0.004	0.002	*	-0.002	0.001	*	0.001	0.001		0.000	0.001		0.000	0.001	
Numeraire category	0.001	-0.042		0.000	-0.056		-0.001	-0.057		0.001	-0.053		0.000	-0.034	
**Total expenditure**	0.005	0.001	*	-0.003	0.001	*	-0.004	0.000	*	0.000	0.001		0.001	0.000	*
**Promotions**															
Take home confectionery	0.007	0.000	*	0.001	0.000	*	0.000	0.000	*	0.001	0.000	*	0.000	0.000	*
Biscuits	0.000	0.000		0.004	0.000	*	0.000	0.000		0.000	0.000	*	0.000	0.000	
Take home savouries	0.000	0.000		0.000	0.000		0.002	0.000	*	0.000	0.000		0.000	0.000	
Cakes, pastries, and sugar morning goods	0.000	0.000		0.000	0.000		0.000	0.000	*	0.002	0.000	*	0.000	0.000	
Total puddings and desserts	0.000	0.000		0.000	0.000	*	0.000	0.000		0.000	0.000	*	0.003	0.000	*
Take home sugary drinks	0.000	0.000		0.000	0.000		0.000	0.000	*	0.000	0.000		0.000	0.000	*
Edible ices and ice creams	0.001	0.000	*	0.000	0.000		0.000	0.000		0.000	0.000		0.000	0.000	
Dairy products	0.000	0.000	*	0.000	0.000	*	0.000	0.000		0.000	0.000		0.000	0.000	
Meat and fish	0.000	0.000		-0.001	0.000	*	0.000	0.000		0.000	0.000		0.000	0.000	*
Fats and eggs	-0.001	0.000	*	0.000	0.000		0.000	0.000	*	0.000	0.000		0.000	0.000	
Fruit	0.000	0.000	*	0.000	0.000		-0.001	0.000	*	0.000	0.000	*	0.000	0.000	*
Vegetables	-0.001	0.000	*	-0.001	0.000	*	0.000	0.000	*	-0.001	0.000	*	0.000	0.000	*
Grains	0.000	0.000	*	0.000	0.000	*	0.000	0.000		0.000	0.000		0.000	0.000	*
Prepared ready to eat foods	-0.001	0.000	*	0.000	0.000		0.001	0.000	*	0.000	0.000		0.000	0.000	*
Sugar and preserves	0.000	0.000		0.000	0.000		0.000	0.000		0.001	0.000	*	0.000	0.000	
Condiments and sauces	0.000	0.000		0.000	0.000		0.000	0.000		0.000	0.000		0.000	0.000	
Low calorie, soft drinks and juices	0.000	0.000		0.000	0.000	*	0.000	0.000	*	0.000	0.000	*	0.000	0.000	*
Alcoholic beverages	-0.002	0.000	*	-0.001	0.000	*	-0.001	0.000	*	-0.001	0.000	*	0.000	0.000	*
Numeraire category	-0.002	0.000	*	-0.001	0.000	*	0.000	0.000		0.000	0.000		0.000	0.000	
Log-Likelihood	265333.000														
Obs.	6175.000														

Variables	Equations														
	Take home sugary drinks			Edible ices and ice cream			Dairy products			Meat and fish			Fats and eggs		
	Coef.	St. Err.	Sig.	Coef.	St. Err.	Sig.	Coef.	St. Err.	Sig.	Coef.	St. Err.	Sig.	Coef.	St. Err.	Sig.
**Intercept**	0.018	0.010	*	0.000	0.007		0.097	0.015	*	0.133	0.025	*	0.030	0.008	*
**Prices**															
Take home confectionery	-0.004	0.002	*	-0.002	0.001		0.006	0.003	*	-0.003	0.005		0.003	0.002	
Biscuits	0.001	0.003		0.002	0.003		-0.003	0.004		0.006	0.006		-0.002	0.003	
Take home savouries	0.006	0.003	*	-0.001	0.003		0.009	0.004	*	-0.015	0.006	*	-0.004	0.003	
Cakes, pastries, and sugar morning goods	0.004	0.003	*	-0.003	0.002		0.000	0.004		0.001	0.006		0.004	0.002	*
Total puddings and desserts	0.001	0.002		0.001	0.002		-0.001	0.003		-0.003	0.004		0.003	0.002	*
Take home sugary drinks	-0.005	0.004		-0.001	0.002		0.006	0.004		-0.015	0.006	*	-0.004	0.002	*
Edible ices and ice creams	-0.001	0.002		0.000	0.003		-0.007	0.003	*	0.003	0.004		0.004	0.002	*
Dairy products	0.006	0.004		-0.007	0.003	*	0.010	0.009		-0.004	0.010		-0.001	0.004	
Meat and fish	-0.015	0.006	*	0.003	0.004		-0.004	0.010		-0.035	0.022		0.002	0.005	
Fats and eggs	-0.004	0.002	*	0.004	0.002	*	-0.001	0.004		0.002	0.005		0.003	0.003	
Fruit	0.001	0.004		0.003	0.003		0.001	0.006		-0.005	0.010		-0.009	0.003	*
Vegetables	0.002	0.004		0.000	0.003		-0.014	0.006	*	0.010	0.009		-0.002	0.003	
Grains	-0.001	0.003		-0.001	0.002		0.001	0.004		0.023	0.007	*	-0.005	0.002	*
Prepared ready to eat foods	0.006	0.006		-0.002	0.005		0.003	0.010		0.064	0.016	*	0.006	0.006	
Sugar and preserves	0.000	0.001		0.000	0.001		-0.003	0.002		0.000	0.003		-0.001	0.001	
Condiments and sauces	0.001	0.001		0.000	0.001		0.003	0.002		-0.001	0.003		0.000	0.001	
Low calorie, soft drinks and juices	0.002	0.002		0.004	0.001	*	0.006	0.003	*	-0.019	0.005	*	0.003	0.002	*
Alcoholic beverages	-0.002	0.001		0.000	0.001		-0.005	0.002	*	-0.001	0.004		0.000	0.001	
Numeraire category	0.000	-0.052		-0.001	-0.040		-0.004	-0.083		-0.009	-0.133		0.000	-0.046	
**Total expenditure**	-0.001	0.001		-0.001	0.000		-0.010	0.001	*	-0.015	0.002	*	-0.001	0.000	*
**Promotions**															
Take home confectionery	0.001	0.000	*	0.000	0.000		0.000	0.000		-0.003	0.001	*	0.000	0.000	*
Biscuits	0.000	0.000		0.000	0.000	*	-0.001	0.000		-0.001	0.001		-0.001	0.000	*
Take home savouries	0.000	0.000		0.000	0.000		-0.001	0.000	*	0.001	0.000	*	0.000	0.000	*
Cakes, pastries, and sugar morning goods	0.000	0.000	*	0.000	0.000		0.001	0.000	*	-0.001	0.001		0.000	0.000	
Total puddings and desserts	0.000	0.000		0.000	0.000	*	0.001	0.000	*	-0.001	0.001	*	0.000	0.000	*
Take home sugary drinks	0.003	0.000	*	0.000	0.000		-0.001	0.000	*	0.000	0.000		0.000	0.000	*
Edible ices and ice creams	0.000	0.000	*	0.003	0.000	*	-0.001	0.000	*	0.000	0.001		0.000	0.000	
Dairy products	0.000	0.000		0.000	0.000		0.003	0.000	*	-0.001	0.000	*	0.000	0.000	
Meat and fish	0.000	0.000		0.000	0.000		-0.001	0.000	*	0.008	0.001	*	0.000	0.000	
Fats and eggs	0.000	0.000	*	0.000	0.000		0.000	0.000		0.002	0.000	*	0.001	0.000	*
Fruit	-0.001	0.000	*	0.000	0.000		0.001	0.000	*	0.000	0.001		0.000	0.000	*
Vegetables	0.000	0.000		0.000	0.000	*	-0.001	0.000		0.001	0.001		0.000	0.000	*
Grains	0.000	0.000		0.000	0.000	*	0.001	0.000	*	-0.001	0.001		0.000	0.000	
Prepared ready to eat foods	0.000	0.000		0.000	0.000		0.001	0.000		-0.004	0.001	*	0.000	0.000	*
Sugar and preserves	0.000	0.000		0.000	0.000		-0.001	0.000	*	0.000	0.001		0.000	0.000	
Condiments and sauces	0.000	0.000		0.000	0.000		0.000	0.000		-0.001	0.001		0.000	0.000	*
Low calorie, soft drinks and juices	0.000	0.000	*	0.000	0.000		-0.001	0.000	*	0.000	0.000		0.000	0.000	*
Alcoholic beverages	-0.001	0.000	*	-0.001	0.000	*	-0.002	0.000	*	-0.001	0.001	*	0.000	0.000	*
Numeraire category	-0.001	0.000	*	-0.001	0.000	*	-0.001	0.001	*	-0.001	0.001		0.000	0.000	

Variables	Equations														
	Fruit			Vegetables			Grains			Prepared ready to eat foods			Sugar and preserves		
	Coef.	St. Err.	Sig.	Coef.	St. Err.	Sig.	Coef.	St. Err.	Sig.	Coef.	St. Err.	Sig.	Coef.	St. Err.	Sig.
**Intercept**	0.071	0.014	*	0.059	0.013	*	0.035	0.009	*	0.046	0.023	*	0.012	0.004	*
**Prices**															
Take home confectionery	0.004	0.003		0.003	0.003		-0.001	0.002		0.004	0.005		0.001	0.001	
Biscuits	0.003	0.004		-0.003	0.004		0.002	0.003		-0.004	0.007		0.002	0.001	*
Take home savouries	-0.006	0.004		-0.001	0.004		0.002	0.003		0.010	0.007		0.000	0.001	
Cakes, pastries, and sugar morning goods	0.001	0.004		0.003	0.004		0.006	0.003	*	-0.009	0.007		0.001	0.001	
Total puddings and desserts	-0.004	0.002	*	0.003	0.002		0.000	0.002		0.010	0.004	*	0.000	0.001	
Take home sugary drinks	0.001	0.004		0.002	0.004		-0.001	0.003		0.006	0.006		0.000	0.001	
Edible ices and ice creams	0.003	0.003		0.000	0.003		-0.001	0.002		-0.002	0.005		0.000	0.001	
Dairy products	0.001	0.006		-0.014	0.006	*	0.001	0.004		0.003	0.010		-0.003	0.002	
Meat and fish	-0.005	0.010		0.010	0.009		0.023	0.007	*	0.064	0.016	*	0.000	0.003	
Fats and eggs	-0.009	0.003	*	-0.002	0.003		-0.005	0.002	*	0.006	0.006		-0.001	0.001	
Fruit	-0.004	0.009		-0.017	0.006	*	-0.007	0.004	*	0.031	0.010	*	-0.001	0.002	
Vegetables	-0.017	0.006	*	-0.016	0.007	*	0.001	0.004		0.027	0.009	*	-0.004	0.002	*
Grains	-0.007	0.004	*	0.001	0.004		0.001	0.004		-0.009	0.007		-0.002	0.001	
Prepared ready to eat foods	0.031	0.010	*	0.027	0.009	*	-0.009	0.007		-0.132	0.023	*	0.008	0.003	*
Sugar and preserves	-0.001	0.002		-0.004	0.002	*	-0.002	0.001		0.008	0.003	*	-0.002	0.001	*
Condiments and sauces	-0.004	0.002	*	0.000	0.002		0.003	0.001	*	0.003	0.003		0.001	0.001	
Low calorie, soft drinks and juices	0.007	0.003	*	0.011	0.003	*	-0.003	0.002		-0.003	0.005		0.001	0.001	
Alcoholic beverages	0.001	0.002		-0.001	0.002		-0.005	0.001	*	0.001	0.003		0.001	0.001	
Numeraire category	0.004	-0.081		-0.002	-0.074		-0.004	-0.054		-0.012	-0.136		-0.001	-0.023	
**Total expenditure**	0.002	0.001	*	-0.001	0.001		-0.011	0.001	*	-0.014	0.001	*	-0.001	0.000	*
**Promotions**															
Take home confectionery	0.000	0.000		-0.001	0.000	*	0.000	0.000		0.002	0.001	*	0.000	0.000	
Biscuits	0.000	0.000		-0.001	0.000	*	0.000	0.000		0.001	0.001		0.000	0.000	*
Take home savouries	0.000	0.000		0.000	0.000		0.000	0.000	*	0.000	0.000		0.000	0.000	
Cakes, pastries, and sugar morning goods	-0.001	0.000	*	-0.001	0.000	*	0.000	0.000		0.000	0.000		0.000	0.000	
Total puddings and desserts	0.000	0.000		-0.001	0.000	*	0.000	0.000	*	0.000	0.000		0.000	0.000	*
Take home sugary drinks	-0.001	0.000	*	-0.001	0.000	*	0.000	0.000		0.000	0.000		0.000	0.000	*
Edible ices and ice creams	0.000	0.000		0.000	0.000		0.000	0.000	*	-0.001	0.000		0.000	0.000	
Dairy products	0.000	0.000		0.000	0.000	*	0.001	0.000	*	-0.001	0.000	*	0.000	0.000	*
Meat and fish	-0.001	0.000	*	0.000	0.000		0.000	0.000		0.000	0.001		0.000	0.000	
Fats and eggs	0.000	0.000		0.000	0.000		0.000	0.000	*	-0.002	0.000	*	0.000	0.000	
Fruit	0.008	0.000	*	0.001	0.000	*	-0.001	0.000	*	-0.001	0.000		0.000	0.000	*
Vegetables	0.001	0.000		0.006	0.000	*	0.000	0.000		0.000	0.001		0.000	0.000	
Grains	-0.001	0.000	*	0.000	0.000		0.003	0.000	*	0.001	0.000	*	0.000	0.000	
Prepared ready to eat foods	-0.002	0.000	*	-0.001	0.000	*	0.001	0.000	*	0.010	0.001	*	0.000	0.000	
Sugar and preserves	-0.002	0.001	*	-0.002	0.000	*	-0.001	0.000	*	0.003	0.001	*	0.003	0.000	*
Condiments and sauces	0.000	0.000		0.001	0.000	*	0.001	0.000	*	0.000	0.001		0.000	0.000	*
Low calorie, soft drinks and juices	0.000	0.000		0.000	0.000		0.000	0.000		0.000	0.000	*	0.000	0.000	*
Alcoholic beverages	-0.002	0.000	*	-0.001	0.000	*	-0.002	0.000	*	-0.003	0.000	*	-0.001	0.000	*
Numeraire category	-0.002	0.001	*	-0.001	0.000	*	-0.001	0.000	*	0.001	0.001		0.000	0.000	

Variables	Equations											
	Condiments and sauces			Low calorie soft drinks and juices			Alcoholic beverages			Numeraire category		
	Coef.	St. Err.	Sig.	Coef.	St. Err.	Sig.	Coef.	St. Err.	Sig.	Coef.	St. Err.	Sig.
**Intercept**	0.023	0.004	*	0.054	0.008	*	0.114	0.009	*	0.151	0.051	*
**Prices**												
Take home confectionery	0.000	0.001		-0.004	0.002	*	0.004	0.002	*	0.001	0.011	
Biscuits	0.000	0.001		0.001	0.002		-0.002	0.001	*	0.000	0.015	
Take home savouries	-0.002	0.001	*	0.003	0.002		0.001	0.001		-0.001	0.015	
Cakes, pastries, and sugar morning goods	-0.002	0.001		-0.005	0.002	*	0.000	0.001		0.001	0.014	
Total puddings and desserts	0.000	0.001		0.000	0.001		0.000	0.001		0.000	0.009	
Take home sugary drinks	0.001	0.001		0.002	0.002		-0.002	0.001		0.000	0.014	
Edible ices and ice creams	0.000	0.001		0.004	0.001	*	0.000	0.001		-0.001	0.011	
Dairy products	0.003	0.002		0.006	0.003	*	-0.005	0.002	*	-0.004	0.023	
Meat and fish	-0.001	0.003		-0.019	0.005	*	-0.001	0.004		-0.009	0.037	
Fats and eggs	0.000	0.001		0.003	0.002	*	0.000	0.001		0.000	0.012	
Fruit	-0.004	0.002	*	0.007	0.003	*	0.001	0.002		0.004	0.022	
Vegetables	0.000	0.002		0.011	0.003	*	-0.001	0.002		-0.002	0.020	
Grains	0.003	0.001	*	-0.003	0.002		-0.005	0.001	*	-0.004	0.014	
Prepared ready to eat foods	0.003	0.003		-0.003	0.005		0.001	0.003		-0.012	0.038	
Sugar and preserves	0.001	0.001		0.001	0.001		0.001	0.001		-0.001	0.006	
Condiments and sauces	-0.001	0.001		0.000	0.001		0.000	0.001		-0.001	0.006	
Low calorie, soft drinks and juices	0.000	0.001		-0.001	0.003		-0.002	0.002		0.000	0.011	
Alcoholic beverages	0.000	0.001		-0.002	0.002		-0.003	0.006		0.014	0.009	
Numeraire category	-0.001	-0.022		0.000	-0.043		0.014	-0.032		0.013	0.284	
**Total expenditure**	-0.003	0.000	*	-0.002	0.001	*	0.008	0.003	*	0.050	0.004	*
**Promotions**												
Take home confectionery	0.000	0.000	*	-0.001	0.000	*	-0.006	0.001	*	-0.001	0.002	
Biscuits	0.000	0.000		0.000	0.000		-0.002	0.001	*	0.002	0.002	
Take home savouries	0.000	0.000	*	0.000	0.000	*	0.002	0.001	*	-0.001	0.001	
Cakes, pastries, and sugar morning goods	0.000	0.000		0.000	0.000		-0.001	0.001		-0.001	0.001	
Total puddings and desserts	0.000	0.000	*	0.001	0.000	*	-0.003	0.001	*	0.000	0.001	
Take home sugary drinks	0.000	0.000		-0.001	0.000	*	0.000	0.000		0.000	0.001	
Edible ices and ice creams	0.000	0.000	*	0.000	0.000		-0.002	0.001	*	-0.001	0.001	
Dairy products	0.000	0.000		0.000	0.000		-0.002	0.000	*	0.001	0.001	
Meat and fish	0.000	0.000	*	0.000	0.000		-0.002	0.001		-0.001	0.002	
Fats and eggs	0.000	0.000	*	0.000	0.000		0.001	0.001	*	-0.001	0.001	
Fruit	0.000	0.000	*	0.000	0.000		-0.002	0.001	*	-0.003	0.001	*
Vegetables	0.000	0.000		0.000	0.000		-0.001	0.001		-0.002	0.002	
Grains	0.000	0.000		0.000	0.000		-0.002	0.001	*	0.000	0.001	
Prepared ready to eat foods	0.000	0.000	*	0.001	0.000	*	-0.003	0.001	*	0.000	0.002	
Sugar and preserves	-0.001	0.000	*	0.001	0.000	*	-0.003	0.001	*	0.000	0.002	
Condiments and sauces	0.002	0.000	*	-0.001	0.000	*	-0.002	0.001	*	0.000	0.002	
Low calorie, soft drinks and juices	0.000	0.000		0.003	0.000	*	0.000	0.000		-0.001	0.001	
Alcoholic beverages	0.000	0.000	*	-0.001	0.000	*	0.025	0.001	*	-0.002	0.001	
Numeraire category	0.000	0.000	*	0.001	0.000	*	0.000	0.001		0.009	0.002	*

Note: '*' indicates statistically significant at 5 per cent.

Table S3 - Scotland - £30,000 - £39,999 income group - All categories - Demand model results

Variables	Take home confectionery			Biscuits			Take home savouries			Cakes, pastries and sugar morning goods			Total puddings and desserts			Take home sugary drinks			Edible ices and ice cream		
	Coef.	St. Err.	Sig.	Coef.	St. Err.	Sig.	Coef.	St. Err.	Sig.	Coef.	St. Err.	Sig.	Coef.	St. Err.	Sig.	Coef.	St. Err.	Sig.	Coef.	St. Err.	Sig.
**Intercept**	0.035	0.009	*	0.030	0.012	*	-0.002	0.012		0.007	0.011		0.010	0.007		0.005	0.012		0.008	0.008	
**Prices**																					
Take home confectionery	-0.021	0.003	*	-0.006	0.002	*	-0.008	0.002	*	0.000	0.002		-0.002	0.001		-0.004	0.002	*	0.001	0.001	
Biscuits	-0.006	0.002	*	0.005	0.006		0.010	0.005	*	-0.004	0.003		-0.004	0.003		0.005	0.003	*	-0.003	0.003	
Take home savouries	-0.008	0.002	*	0.010	0.005	*	-0.002	0.007		0.003	0.003		0.004	0.003		0.000	0.003		0.005	0.003	
Cakes, pastries, and sugar morning goods	0.000	0.002		-0.004	0.003		0.003	0.003		0.000	0.004		0.001	0.002		-0.008	0.003	*	0.004	0.002	
Total puddings and desserts	-0.002	0.001		-0.004	0.003		0.004	0.003		0.001	0.002		-0.005	0.002	*	-0.001	0.002		0.000	0.002	
Take home sugary drinks	-0.004	0.002	*	0.005	0.003	*	0.000	0.003		-0.008	0.003	*	-0.001	0.002		-0.012	0.004	*	0.001	0.002	
Edible ices and ice creams	0.001	0.001		-0.003	0.003		0.005	0.003		0.004	0.002		0.000	0.002		0.001	0.002		0.004	0.003	
Dairy products	0.011	0.004	*	0.000	0.005		0.007	0.005		-0.002	0.005		-0.005	0.003		0.014	0.005	*	-0.001	0.003	
Meat and fish	0.003	0.007		-0.016	0.008	*	0.002	0.008		0.020	0.008	*	-0.004	0.005		0.002	0.008		-0.007	0.005	
Fats and eggs	0.004	0.002	*	0.001	0.003		-0.003	0.003		-0.001	0.003		0.001	0.002		0.004	0.002		-0.005	0.002	*
Fruit	0.020	0.004	*	0.011	0.005	*	-0.010	0.005	*	-0.013	0.004	*	0.001	0.003		0.001	0.005		0.005	0.003	
Vegetables	0.010	0.004	*	-0.004	0.005		-0.002	0.005		0.000	0.004		-0.002	0.003		0.004	0.004		0.000	0.003	
Grains	-0.001	0.002		0.007	0.003	*	0.004	0.003		-0.001	0.003		0.002	0.002		0.005	0.003	*	-0.005	0.002	*
Prepared ready to eat foods	-0.002	0.006		-0.008	0.009		-0.005	0.009		0.002	0.008		0.013	0.005	*	-0.012	0.008		0.003	0.006	
Sugar and preserves	0.000	0.001		0.002	0.002		0.002	0.002		-0.001	0.001		0.001	0.001		0.001	0.001		-0.001	0.001	
Condiments and sauces	-0.002	0.001	*	0.000	0.001		0.000	0.001		-0.001	0.001		-0.001	0.001		-0.001	0.001		0.001	0.001	
Low calorie, soft drinks and juices	0.000	0.002		0.004	0.002		-0.003	0.002		0.002	0.002		0.000	0.001		-0.001	0.003		-0.001	0.002	
Alcoholic beverages	-0.002	0.002		0.000	0.001		-0.002	0.001		0.001	0.001		0.000	0.001		0.000	0.002		0.000	0.001	
Numeraire category	-0.001	-0.051		0.000	-0.069		-0.002	-0.071		-0.001	-0.062		0.000	-0.041		0.003	-0.061		0.000	-0.047	
**Total expenditure**	0.002	0.001		0.000	0.001		-0.006	0.001	*	-0.002	0.001	*	0.000	0.000		0.001	0.001		0.000	0.000	
**Promotions**																					
Take home confectionery	0.006	0.000	*	0.000	0.000		0.000	0.000	*	0.001	0.000	*	0.000	0.000		0.000	0.000		0.000	0.000	
Biscuits	0.000	0.000		0.005	0.000	*	0.000	0.000		0.000	0.000	*	0.000	0.000	*	0.000	0.000		0.000	0.000	
Take home savouries	0.000	0.000		0.000	0.000	*	0.002	0.000	*	0.000	0.000		0.000	0.000		0.000	0.000	*	0.000	0.000	*
Cakes, pastries, and sugar morning goods	0.000	0.000	*	0.000	0.000		0.000	0.000		0.001	0.000	*	0.000	0.000		0.000	0.000	*	0.000	0.000	
Total puddings and desserts	0.001	0.000	*	0.001	0.000	*	0.000	0.000		0.000	0.000		0.002	0.000	*	0.000	0.000		0.000	0.000	
Take home sugary drinks	0.000	0.000		0.000	0.000		0.000	0.000	*	0.000	0.000	*	0.000	0.000		0.003	0.000	*	0.000	0.000	
Edible ices and ice creams	0.001	0.000	*	0.000	0.000		0.000	0.000		0.000	0.000		0.000	0.000		0.000	0.000	*	0.002	0.000	*
Dairy products	0.000	0.000		0.000	0.000		0.000	0.000		0.000	0.000		0.000	0.000	*	0.000	0.000		0.000	0.000	
Meat and fish	-0.001	0.000		0.000	0.000		0.000	0.000		0.000	0.000		0.000	0.000		0.000	0.000		0.000	0.000	*
Fats and eggs	0.000	0.000		0.000	0.000		0.000	0.000	*	0.000	0.000		0.000	0.000		0.000	0.000		0.000	0.000	*
Fruit	0.000	0.000		0.000	0.000	*	0.000	0.000		-0.001	0.000	*	0.000	0.000	*	-0.001	0.000	*	0.000	0.000	
Vegetables	-0.001	0.000	*	0.000	0.000		-0.001	0.000	*	-0.001	0.000	*	0.000	0.000	*	0.000	0.000		0.000	0.000	
Grains	0.000	0.000		0.000	0.000	*	0.000	0.000		0.000	0.000		0.000	0.000		0.000	0.000	*	0.000	0.000	
Prepared ready to eat foods	-0.001	0.000		-0.001	0.000	*	0.001	0.000		0.001	0.000		0.000	0.000		0.001	0.000		0.000	0.000	
Sugar and preserves	0.000	0.001		0.000	0.000		-0.001	0.000	*	0.000	0.000		0.000	0.000		-0.001	0.000	*	0.000	0.000	*
Condiments and sauces	0.000	0.001		-0.001	0.000	*	0.000	0.000		0.000	0.000		0.000	0.000		-0.001	0.001		0.000	0.000	
Low calorie, soft drinks and juices	0.000	0.000	*	0.000	0.000	*	0.000	0.000		0.000	0.000	*	0.000	0.000	*	0.000	0.000	*	0.000	0.000	
Alcoholic beverages	-0.001	0.000	*	-0.001	0.000	*	-0.001	0.000	*	-0.001	0.000	*	0.000	0.000	*	-0.001	0.000	*	0.000	0.000	*
Numeraire category	-0.001	0.000	*	-0.001	0.000	*	-0.001	0.000	*	0.000	0.000		0.000	0.000	*	-0.001	0.000	*	0.000	0.000	*
Log-Likelihood	77210.900																				
Obs.	1701.000																				

Variables	Dairy products			Meat and fish			Fats and eggs			Fruit			Vegetables			Grains			Prepared ready to eat foods		
	Coef.	St. Err.	Sig.	Coef.	St. Err.	Sig.	Coef.	St. Err.	Sig.	Coef.	St. Err.	Sig.	Coef.	St. Err.	Sig.	Coef.	St. Err.	Sig.	Coef.	St. Err.	Sig.
**Intercept**	0.096	0.018	*	0.179	0.031	*	0.045	0.010	*	0.045	0.017	*	0.085	0.016	*	0.033	0.011	*	0.078	0.028	*
**Prices**																					
Take home confectionery	0.011	0.004	*	0.003	0.007		0.004	0.002	*	0.020	0.004	*	0.010	0.004	*	-0.001	0.002		-0.002	0.006	
Biscuits	0.000	0.005		-0.016	0.008	*	0.001	0.003		0.011	0.005	*	-0.004	0.005		0.007	0.003	*	-0.008	0.009	
Take home savouries	0.007	0.005		0.002	0.008		-0.003	0.003		-0.010	0.005	*	-0.002	0.005		0.004	0.003		-0.005	0.009	
Cakes, pastries, and sugar morning goods	-0.002	0.005		0.020	0.008	*	-0.001	0.003		-0.013	0.004	*	0.000	0.004		-0.001	0.003		0.002	0.008	
Total puddings and desserts	-0.005	0.003		-0.004	0.005		0.001	0.002		0.001	0.003		-0.002	0.003		0.002	0.002		0.013	0.005	*
Take home sugary drinks	0.014	0.005	*	0.002	0.008		0.004	0.002		0.001	0.005		0.004	0.004		0.005	0.003	*	-0.012	0.008	
Edible ices and ice creams	-0.001	0.003		-0.007	0.005		-0.005	0.002	*	0.005	0.003		0.000	0.003		-0.005	0.002	*	0.003	0.006	
Dairy products	-0.024	0.010	*	0.002	0.012		0.003	0.004		0.009	0.007		-0.008	0.007		-0.002	0.005		0.006	0.013	
Meat and fish	0.002	0.012		0.000	0.029		-0.006	0.006		0.011	0.012		0.000	0.011		0.004	0.008		-0.015	0.021	
Fats and eggs	0.003	0.004		-0.006	0.006		0.006	0.003	*	-0.007	0.004	*	0.000	0.004		0.000	0.003		0.003	0.007	
Fruit	0.009	0.007		0.011	0.012		-0.007	0.004	*	0.025	0.010	*	-0.011	0.007		-0.008	0.005	*	-0.031	0.012	*
Vegetables	-0.008	0.007		0.000	0.011		0.000	0.004		-0.011	0.007		0.010	0.009		-0.005	0.004		0.014	0.011	
Grains	-0.002	0.005		0.004	0.008		0.000	0.003		-0.008	0.005	*	-0.005	0.004		-0.007	0.004	*	0.002	0.008	
Prepared ready to eat foods	0.006	0.013		-0.015	0.021		0.003	0.007		-0.031	0.012	*	0.014	0.011		0.002	0.008		0.030	0.029	
Sugar and preserves	0.000	0.002		0.003	0.004		0.000	0.001		-0.001	0.002		-0.005	0.002	*	0.000	0.001		0.004	0.004	
Condiments and sauces	-0.004	0.002	*	0.006	0.003	*	0.000	0.001		0.000	0.002		0.005	0.002	*	0.002	0.001	*	-0.002	0.003	
Low calorie, soft drinks and juices	0.001	0.004		-0.016	0.007	*	0.002	0.002		0.006	0.004		0.005	0.004		0.005	0.003	*	0.003	0.006	
Alcoholic beverages	-0.002	0.003		0.005	0.005		0.001	0.001		-0.001	0.003		-0.006	0.002	*	-0.002	0.002		0.002	0.004	
Numeraire category	-0.006	-0.099		0.005	-0.167		-0.002	-0.054		-0.007	-0.097		-0.003	-0.090		-0.002	-0.063		-0.006	-0.168	
**Total expenditure**	-0.004	0.001	*	-0.010	0.003	*	0.000	0.001		0.000	0.002		0.000	0.001		-0.009	0.001	*	-0.015	0.002	*
**Promotions**																					
Take home confectionery	-0.001	0.001		0.002	0.001	*	0.000	0.000		-0.002	0.001	*	-0.001	0.001	*	0.000	0.000		0.002	0.001	*
Biscuits	0.000	0.000		0.000	0.001		0.000	0.000	*	0.000	0.001		-0.001	0.000	*	0.001	0.000	*	-0.001	0.001	
Take home savouries	0.001	0.000	*	-0.002	0.001	*	0.000	0.000		0.000	0.000		0.000	0.000		0.001	0.000	*	0.000	0.001	
Cakes, pastries, and sugar morning goods	0.001	0.000	*	0.001	0.001		0.000	0.000		0.001	0.000	*	0.001	0.000	*	0.000	0.000		0.000	0.000	
Total puddings and desserts	0.000	0.000		-0.002	0.001	*	0.000	0.000		0.001	0.000		-0.001	0.000	*	0.000	0.000		0.001	0.001	
Take home sugary drinks	0.000	0.000		0.000	0.000		0.000	0.000	*	-0.001	0.000	*	-0.001	0.000	*	0.000	0.000		0.001	0.000	
Edible ices and ice creams	-0.001	0.000	*	-0.002	0.001	*	0.000	0.000	*	0.000	0.000		-0.001	0.000	*	0.000	0.000	*	0.000	0.001	
Dairy products	0.002	0.000	*	-0.001	0.000	*	0.000	0.000		0.000	0.000		-0.001	0.000	*	0.000	0.000	*	-0.001	0.000	*
Meat and fish	0.000	0.001		0.007	0.001	*	0.000	0.000		-0.001	0.001		0.000	0.000		0.001	0.000	*	0.001	0.001	
Fats and eggs	-0.001	0.000	*	0.002	0.001	*	0.002	0.000	*	0.001	0.000	*	0.000	0.000		0.000	0.000		-0.001	0.000	*
Fruit	0.001	0.000	*	-0.001	0.001	*	0.000	0.000	*	0.004	0.000	*	0.000	0.000		0.000	0.000		0.000	0.000	
Vegetables	0.000	0.001		0.001	0.001		0.000	0.000		0.001	0.001	*	0.005	0.001	*	-0.001	0.000	*	-0.002	0.001	*
Grains	0.001	0.000	*	-0.001	0.001	*	0.000	0.000		0.000	0.000		-0.001	0.000	*	0.002	0.000	*	0.001	0.001	
Prepared ready to eat foods	-0.001	0.001		-0.004	0.001	*	-0.002	0.000	*	-0.001	0.001		-0.003	0.001	*	-0.001	0.000		0.014	0.001	*
Sugar and preserves	0.000	0.001		0.000	0.001		0.000	0.000		0.000	0.001		0.001	0.001		0.000	0.000		-0.003	0.001	*
Condiments and sauces	0.000	0.001		-0.004	0.002	*	0.000	0.000		0.000	0.001		0.002	0.001	*	0.002	0.001	*	0.000	0.001	
Low calorie, soft drinks and juices	0.000	0.000	*	0.000	0.000		0.000	0.000		0.000	0.000		0.000	0.000		0.000	0.000	*	0.000	0.000	
Alcoholic beverages	-0.003	0.000	*	-0.001	0.001		0.000	0.000	*	-0.003	0.000	*	-0.001	0.000	*	-0.002	0.000	*	-0.003	0.001	*
Numeraire category	-0.002	0.001	*	0.000	0.001		0.000	0.000		-0.003	0.001	*	0.000	0.001		0.000	0.000		0.000	0.001	

Variables	Sugar and preserves			Condiments and sauces			Low calorie soft drinks and juices			Alcoholic beverages			Numeraire category		
	Coef.	St. Err.	Sig.	Coef.	St. Err.	Sig.	Coef.	St. Err.	Sig.	Coef.	St. Err.	Sig.	Coef.	St. Err.	Sig.
**Intercept**	0.015	0.005	*	0.015	0.005	*	0.058	0.009	*	0.112	0.011	*	0.148	0.062	*
**Prices**															
Take home confectionery	0.000	0.001		-0.002	0.001	*	0.000	0.002		-0.002	0.002		-0.001	0.014	
Biscuits	0.002	0.002		0.000	0.001		0.004	0.002		0.000	0.001		0.000	0.018	
Take home savouries	0.002	0.002		0.000	0.001		-0.003	0.002		-0.002	0.001		-0.002	0.019	
Cakes, pastries, and sugar morning goods	-0.001	0.001		-0.001	0.001		0.002	0.002		0.001	0.001		-0.001	0.016	
Total puddings and desserts	0.001	0.001		-0.001	0.001		0.000	0.001		0.000	0.001		0.000	0.011	
Take home sugary drinks	0.001	0.001		-0.001	0.001		-0.001	0.003		0.000	0.002		0.003	0.016	
Edible ices and ice creams	-0.001	0.001		0.001	0.001		-0.001	0.002		0.000	0.001		0.000	0.012	
Dairy products	0.000	0.002		-0.004	0.002	*	0.001	0.004		-0.002	0.003		-0.006	0.027	
Meat and fish	0.003	0.004		0.006	0.003	*	-0.016	0.007	*	0.005	0.005		0.005	0.047	
Fats and eggs	0.000	0.001		0.000	0.001		0.002	0.002		0.001	0.001		-0.002	0.014	
Fruit	-0.001	0.002		0.000	0.002		0.006	0.004		-0.001	0.003		-0.007	0.027	
Vegetables	-0.005	0.002	*	0.005	0.002	*	0.005	0.004		-0.006	0.002	*	-0.003	0.024	
Grains	0.000	0.001		0.002	0.001	*	0.005	0.003	*	-0.002	0.002		-0.002	0.017	
Prepared ready to eat foods	0.004	0.004		-0.002	0.003		0.003	0.006		0.002	0.004		-0.006	0.047	
Sugar and preserves	-0.003	0.001	*	-0.001	0.001		-0.001	0.001		0.001	0.001		-0.002	0.008	
Condiments and sauces	-0.001	0.001		0.000	0.001		0.001	0.001		-0.002	0.001	*	-0.002	0.007	
Low calorie, soft drinks and juices	-0.001	0.001		0.001	0.001		-0.005	0.003		-0.002	0.002		0.000	0.014	
Alcoholic beverages	0.001	0.001		-0.002	0.001	*	-0.002	0.002		-0.002	0.008		0.009	0.012	
Numeraire category	-0.002	-0.030		-0.002	-0.026		0.000	-0.053		0.009	-0.041		0.015	0.347	
**Total expenditure**	-0.001	0.000		-0.002	0.000	*	0.001	0.001		0.000	0.004		0.047	0.007	*
**Promotions**															
Take home confectionery	0.000	0.000		0.000	0.000	*	0.000	0.000		-0.003	0.002	*	-0.003	0.003	
Biscuits	0.000	0.000	*	0.000	0.000		0.000	0.000		-0.004	0.001	*	0.001	0.002	
Take home savouries	0.000	0.000	*	0.000	0.000		0.001	0.000	*	-0.001	0.001		-0.001	0.002	
Cakes, pastries, and sugar morning goods	0.000	0.000		0.000	0.000		0.000	0.000		-0.003	0.001	*	-0.001	0.001	
Total puddings and desserts	0.000	0.000		0.000	0.000		0.000	0.000		-0.002	0.001		-0.001	0.002	
Take home sugary drinks	0.000	0.000		0.000	0.000		0.000	0.000	*	-0.001	0.001	*	0.000	0.001	
Edible ices and ice creams	0.000	0.000		0.000	0.000	*	0.000	0.000		0.001	0.001		0.001	0.002	
Dairy products	0.000	0.000		0.000	0.000	*	0.000	0.000		0.000	0.001		0.000	0.001	
Meat and fish	0.000	0.000		0.000	0.000		0.000	0.000		-0.004	0.002	*	-0.002	0.002	
Fats and eggs	0.000	0.000		0.000	0.000		0.000	0.000	*	0.000	0.001		-0.001	0.001	
Fruit	0.000	0.000	*	0.000	0.000		0.000	0.000	*	-0.001	0.001		0.000	0.001	
Vegetables	0.000	0.000		0.000	0.000		-0.002	0.000	*	0.003	0.002		-0.002	0.003	
Grains	0.000	0.000		0.000	0.000		0.000	0.000		-0.003	0.001	*	0.000	0.002	
Prepared ready to eat foods	0.000	0.000	*	-0.001	0.000	*	0.000	0.000		-0.001	0.002		0.000	0.003	
Sugar and preserves	0.003	0.000	*	0.000	0.000	*	0.000	0.001		0.003	0.002		0.000	0.003	
Condiments and sauces	0.000	0.000		0.005	0.000	*	0.000	0.001		-0.003	0.003		0.000	0.004	
Low calorie, soft drinks and juices	0.000	0.000		0.000	0.000		0.002	0.000	*	0.001	0.001	*	-0.001	0.001	
Alcoholic beverages	0.000	0.000	*	0.000	0.000	*	-0.001	0.000	*	0.022	0.001	*	-0.001	0.002	
Numeraire category	0.000	0.000	*	0.000	0.000		-0.001	0.000	*	-0.004	0.002	*	0.014	0.003	*

Note: '*' indicates statistically significant at 5 per cent.

Table S4 - Scotland - £40,000 - £49,999 income group - All categories - Demand model results

Equations (1 of 3)

Variables	Take home confectionery Coef.	St. Err.	Sig.	Biscuits Coef.	St. Err.	Sig.	Take home savouries Coef.	St. Err.	Sig.	Cakes, pastries and sugar morning goods Coef.	St. Err.	Sig.	Total puddings and desserts Coef.	St. Err.	Sig.	Take home sugary drinks Coef.	St. Err.	Sig.
**Intercept**	0.050	0.011	*	-0.014	0.013		0.010	0.014		0.002	0.013		0.002	0.008		-0.004	0.014	
**Prices**																		
Take home confectionery	-0.005	0.004		-0.003	0.002		-0.004	0.003		-0.007	0.003	*	0.000	0.001		0.002	0.003	
Biscuits	-0.003	0.002		0.007	0.007		-0.002	0.005		-0.009	0.004	*	0.002	0.003		-0.003	0.003	
Take home savouries	-0.004	0.003		-0.002	0.005		0.009	0.008		-0.012	0.004	*	-0.002	0.003		-0.001	0.004	
Cakes, pastries, and sugar morning goods	-0.007	0.003	*	-0.009	0.004	*	-0.012	0.004	*	0.000	0.005		0.003	0.002		-0.002	0.004	
Total puddings and desserts	0.000	0.001		0.002	0.003		-0.002	0.003		0.003	0.002		0.000	0.002		0.002	0.002	
Take home sugary drinks	0.002	0.003		-0.003	0.003		-0.001	0.004		-0.002	0.004		0.002	0.002		-0.009	0.005	*
Edible ices and ice creams	0.005	0.002	*	0.004	0.003		-0.004	0.004		0.003	0.003		0.000	0.002		0.004	0.002	*
Dairy products	0.009	0.005	*	0.000	0.006		0.010	0.006		-0.001	0.006		-0.002	0.003		0.006	0.006	
Meat and fish	-0.016	0.008	*	0.012	0.009		-0.006	0.010		0.023	0.009	*	-0.004	0.005		-0.011	0.009	
Fats and eggs	0.006	0.002	*	0.000	0.004		0.000	0.004		-0.006	0.003	*	-0.005	0.002	*	-0.003	0.003	
Fruit	0.008	0.006		-0.012	0.006	*	-0.003	0.006		-0.001	0.006		0.004	0.003		0.009	0.006	
Vegetables	-0.009	0.004	*	0.005	0.005		0.001	0.006		0.006	0.005		-0.002	0.003		0.001	0.005	
Grains	0.002	0.003		0.000	0.003		-0.002	0.004		-0.004	0.004		0.000	0.002		-0.008	0.004	*
Prepared ready to eat foods	0.006	0.008		0.000	0.010		0.023	0.011	*	0.008	0.010		0.002	0.006		0.003	0.010	
Sugar and preserves	0.001	0.001		0.000	0.002		-0.002	0.002		-0.001	0.002		0.001	0.001		0.002	0.002	
Condiments and sauces	-0.004	0.002	*	0.000	0.002		-0.002	0.002		0.001	0.002		0.002	0.001	*	0.002	0.002	
Low calorie, soft drinks and juices	0.001	0.003		0.002	0.003		0.000	0.003		0.002	0.003		0.003	0.002	*	0.004	0.003	
Alcoholic beverages	0.005	0.002	*	0.000	0.001		0.001	0.002		0.001	0.002		-0.002	0.001	*	0.003	0.002	
Numeraire category	0.001	-0.062		-0.004	-0.078		-0.005	-0.085		-0.004	-0.076		-0.002	-0.044		-0.001	-0.075	
**Total expenditure**	0.003	0.001	*	-0.003	0.001	*	-0.008	0.001	*	0.000	0.001		-0.001	0.000		0.002	0.001	*
**Promotions**																		
Take home confectionery	0.005	0.000	*	0.000	0.000	*	0.000	0.000		0.000	0.000		0.000	0.000		0.000	0.000	
Biscuits	0.000	0.001		0.004	0.000	*	0.001	0.000	*	0.000	0.000		0.001	0.000	*	0.000	0.000	
Take home savouries	0.000	0.000		0.000	0.000	*	0.003	0.000	*	0.000	0.000		0.000	0.000		0.000	0.000	
Cakes, pastries, and sugar morning goods	-0.001	0.000	*	0.000	0.000		0.000	0.000		0.002	0.000	*	0.000	0.000		0.001	0.000	*
Total puddings and desserts	0.001	0.000	*	0.000	0.000		0.001	0.000	*	0.001	0.000	*	0.002	0.000	*	0.000	0.000	
Take home sugary drinks	0.000	0.000		0.000	0.000	*	0.000	0.000	*	0.000	0.000		0.000	0.000		0.002	0.000	*
Edible ices and ice creams	0.000	0.000		0.000	0.000		-0.001	0.000	*	0.000	0.000		0.000	0.000		0.000	0.000	
Dairy products	0.000	0.000		0.000	0.000		0.000	0.000		0.000	0.000		0.000	0.000	*	0.000	0.000	
Meat and fish	-0.001	0.001		0.000	0.000		0.000	0.000		0.001	0.000		0.000	0.000	*	0.000	0.000	
Fats and eggs	0.000	0.000		0.000	0.000	*	-0.001	0.000	*	0.000	0.000		0.000	0.000		-0.001	0.000	*
Fruit	0.000	0.000		0.000	0.000		0.000	0.000		0.000	0.000		0.000	0.000		0.000	0.000	
Vegetables	-0.001	0.001	*	0.001	0.000		0.000	0.000		-0.001	0.000	*	-0.001	0.000	*	-0.001	0.000	
Grains	-0.001	0.000		0.000	0.000		0.000	0.000		0.000	0.000		0.000	0.000		0.000	0.000	
Prepared ready to eat foods	0.000	0.001		-0.001	0.000	*	0.000	0.000		0.000	0.000		0.000	0.000		0.000	0.000	
Sugar and preserves	0.001	0.001		0.001	0.001	*	0.000	0.001		0.003	0.001	*	0.000	0.000		0.001	0.001	
Condiments and sauces	0.000	0.001		0.000	0.000		0.000	0.000		0.000	0.001		-0.001	0.000	*	0.000	0.001	
Low calorie, soft drinks and juices	0.000	0.000		0.000	0.000	*	0.000	0.000	*	0.000	0.000		0.000	0.000		0.000	0.000	
Alcoholic beverages	-0.001	0.000	*	-0.001	0.000	*	-0.001	0.000	*	-0.001	0.000	*	0.000	0.000		0.000	0.000	
Numeraire category	-0.003	0.001	*	-0.002	0.000	*	-0.001	0.000	*	-0.001	0.001		0.000	0.000	*	-0.001	0.001	*
Log-Likelihood	52643.000																	
Obs.	1168.000																	

Equations (2 of 3)

Variables	Edible ices and ice cream Coef.	St. Err.	Sig.	Dairy products Coef.	St. Err.	Sig.	Meat and fish Coef.	St. Err.	Sig.	Fats and eggs Coef.	St. Err.	Sig.	Fruit Coef.	St. Err.	Sig.	Vegetables Coef.	St. Err.	Sig.
**Intercept**	0.021	0.009	*	0.090	0.023	*	0.100	0.037	*	0.021	0.012	*	0.112	0.024	*	0.112	0.020	*
**Prices**																		
Take home confectionery	0.005	0.002	*	0.009	0.005	*	-0.016	0.008	*	0.006	0.002	*	0.008	0.006		-0.009	0.004	*
Biscuits	0.004	0.003		0.000	0.006		0.012	0.009		0.000	0.004		-0.012	0.006	*	0.005	0.005	
Take home savouries	-0.004	0.004		0.010	0.006		-0.006	0.010		0.000	0.004		-0.003	0.006		0.001	0.006	
Cakes, pastries, and sugar morning goods	0.003	0.003		-0.001	0.006		0.023	0.009	*	-0.006	0.003	*	-0.001	0.006		0.006	0.005	
Total puddings and desserts	0.000	0.002		-0.002	0.003		-0.004	0.005		-0.005	0.002	*	0.004	0.003		-0.002	0.003	
Take home sugary drinks	0.004	0.002	*	0.006	0.006		-0.011	0.009		-0.003	0.003		0.009	0.006		0.001	0.005	
Edible ices and ice creams	-0.004	0.004		0.003	0.004		-0.002	0.006		0.003	0.003		0.004	0.004		-0.003	0.004	
Dairy products	0.003	0.004		-0.022	0.014		0.003	0.016		0.004	0.006		0.019	0.011	*	-0.004	0.009	
Meat and fish	-0.002	0.006		0.003	0.016		0.009	0.034		0.016	0.008	*	0.006	0.017		-0.046	0.014	*
Fats and eggs	0.003	0.003		0.004	0.006		0.016	0.008	*	0.006	0.005		-0.018	0.006	*	0.000	0.005	
Fruit	0.004	0.004		0.019	0.011	*	0.006	0.017		-0.018	0.006	*	-0.004	0.016		0.009	0.010	
Vegetables	-0.003	0.004		-0.004	0.009		-0.046	0.014	*	0.000	0.005		0.009	0.010		0.029	0.011	*
Grains	-0.002	0.002		-0.001	0.006		0.010	0.010		0.001	0.003		-0.010	0.007		0.002	0.005	
Prepared ready to eat foods	-0.010	0.007		-0.021	0.016		-0.002	0.025		-0.007	0.009		0.000	0.018		0.009	0.014	
Sugar and preserves	0.000	0.001		-0.005	0.003	*	0.004	0.005		0.002	0.002		-0.007	0.003	*	-0.001	0.003	
Condiments and sauces	0.000	0.001		0.005	0.003		0.003	0.005		-0.001	0.002		0.002	0.003		0.003	0.003	
Low calorie, soft drinks and juices	-0.001	0.002		-0.001	0.005		0.000	0.009		0.004	0.003		0.003	0.006		0.000	0.005	
Alcoholic beverages	0.000	0.001		-0.003	0.003		0.004	0.005		0.000	0.002		-0.013	0.004	*	-0.002	0.003	
Numeraire category	-0.002	-0.054		0.001	-0.129		-0.004	-0.203		-0.002	-0.071		0.003	-0.139		0.003	-0.113	
**Total expenditure**	0.000	0.000		-0.008	0.002	*	-0.006	0.003	*	-0.001	0.001	*	-0.003	0.002		-0.006	0.002	*
**Promotions**																		
Take home confectionery	0.000	0.000		-0.001	0.001		0.000	0.001		0.000	0.000		-0.001	0.001		0.000	0.001	
Biscuits	0.000	0.000		0.000	0.001		-0.001	0.001		0.000	0.000		-0.002	0.001	*	-0.001	0.001	
Take home savouries	0.000	0.000		-0.001	0.000	*	0.001	0.001		0.000	0.000		0.000	0.001		-0.001	0.000	*
Cakes, pastries, and sugar morning goods	0.000	0.000		0.000	0.001		0.000	0.001		0.000	0.000		0.000	0.001		0.001	0.000	
Total puddings and desserts	0.000	0.000		0.000	0.001		-0.001	0.001		-0.001	0.000	*	0.001	0.001		-0.002	0.001	*
Take home sugary drinks	0.000	0.000		-0.001	0.000	*	0.000	0.000		0.000	0.000	*	-0.001	0.000	*	-0.001	0.000	*
Edible ices and ice creams	0.004	0.000	*	-0.001	0.001		-0.003	0.001	*	-0.001	0.000	*	0.001	0.001		0.000	0.000	
Dairy products	0.000	0.000		0.003	0.000	*	-0.001	0.000	*	0.000	0.000		0.000	0.000		0.000	0.000	
Meat and fish	0.000	0.000	*	-0.001	0.001		0.009	0.001	*	0.000	0.000		0.000	0.001		-0.001	0.001	
Fats and eggs	0.000	0.000		0.002	0.000	*	0.000	0.001		0.003	0.000	*	-0.001	0.001		0.000	0.000	
Fruit	0.000	0.000		-0.002	0.000	*	0.001	0.001		0.000	0.000	*	0.005	0.001	*	0.000	0.000	
Vegetables	0.000	0.000		0.002	0.001	*	0.002	0.001		0.001	0.000	*	0.002	0.001	*	0.006	0.001	*
Grains	0.000	0.000		0.002	0.001	*	0.000	0.001		0.000	0.000		0.001	0.001	*	0.000	0.000	
Prepared ready to eat foods	0.000	0.000		0.003	0.001	*	-0.004	0.001	*	-0.001	0.000	*	-0.002	0.001		-0.001	0.001	
Sugar and preserves	0.001	0.000	*	0.001	0.001		-0.005	0.002	*	0.000	0.001		-0.001	0.002		-0.003	0.001	*
Condiments and sauces	0.000	0.000		-0.001	0.001		0.001	0.002		0.000	0.000		-0.003	0.001	*	0.000	0.001	
Low calorie, soft drinks and juices	0.000	0.000		0.000	0.000		-0.001	0.000		0.000	0.000	*	-0.001	0.000	*	-0.001	0.000	*
Alcoholic beverages	0.000	0.000		-0.003	0.001	*	-0.003	0.001	*	0.000	0.000	*	-0.003	0.001	*	-0.002	0.001	*
Numeraire category	0.000	0.000	*	-0.001	0.001		-0.006	0.002	*	-0.001	0.000	*	-0.002	0.001	*	-0.001	0.001	

Equations (3 of 3)

Variables	Grains Coef.	St. Err.	Sig.	Prepared ready to eat foods Coef.	St. Err.	Sig.	Sugar and preserves Coef.	St. Err.	Sig.	Condiments and sauces Coef.	St. Err.	Sig.	Low calorie soft drinks and juices Coef.	St. Err.	Sig.	Alcoholic beverages Coef.	St. Err.	Sig.	Numeraire category Coef.	St. Err.	Sig.
**Intercept**	0.007	0.013		0.107	0.036	*	0.014	0.007	*	0.030	0.007	*	0.045	0.012	*	0.129	0.014	*	0.167	0.077	*
**Prices**																					
Take home confectionery	0.002	0.003		0.006	0.008		0.001	0.001		-0.004	0.002	*	0.001	0.003		0.005	0.002	*	0.001	0.017	
Biscuits	0.000	0.003		0.000	0.010		0.000	0.002		0.000	0.002		0.002	0.003		0.000	0.001		-0.004	0.021	
Take home savouries	-0.002	0.004		0.023	0.011	*	-0.002	0.002		-0.002	0.002		0.000	0.003		0.001	0.002		-0.005	0.023	
Cakes, pastries, and sugar morning goods	-0.004	0.004		0.008	0.010		-0.001	0.002		0.001	0.002		0.002	0.003		0.001	0.002		-0.004	0.020	
Total puddings and desserts	0.000	0.002		0.002	0.006		0.001	0.001		0.002	0.001	*	0.003	0.002	*	-0.002	0.001	*	-0.002	0.012	
Take home sugary drinks	-0.008	0.004	*	0.003	0.010		0.002	0.002		0.002	0.002		0.004	0.003		0.003	0.002		-0.001	0.020	
Edible ices and ice creams	-0.002	0.002		-0.010	0.007		0.000	0.001		0.000	0.001		-0.001	0.002		0.000	0.001		-0.002	0.014	
Dairy products	-0.001	0.006		-0.021	0.016		-0.005	0.003	*	0.005	0.003		-0.001	0.005		-0.003	0.003		0.001	0.035	
Meat and fish	0.010	0.010		-0.002	0.025		0.004	0.005		0.003	0.005		0.000	0.009		0.004	0.005		-0.004	0.057	
Fats and eggs	0.001	0.003		-0.007	0.009		0.002	0.002		-0.001	0.002		0.004	0.003		0.000	0.002		-0.002	0.019	
Fruit	-0.010	0.007		0.000	0.018		-0.007	0.003	*	0.002	0.003		0.003	0.006		-0.013	0.004	*	0.003	0.038	
Vegetables	0.002	0.005		0.009	0.014		-0.001	0.003		0.003	0.003		0.000	0.005		-0.002	0.003		0.003	0.031	
Grains	0.004	0.005		0.022	0.010	*	0.002	0.002		0.002	0.002		-0.011	0.004	*	0.000	0.002		-0.005	0.021	
Prepared ready to eat foods	0.022	0.010	*	-0.023	0.036		0.011	0.005	*	-0.013	0.005	*	-0.012	0.009		0.003	0.005		0.000	0.060	
Sugar and preserves	0.002	0.002		0.011	0.005	*	-0.002	0.001		0.000	0.001		0.000	0.002		-0.002	0.001	*	-0.002	0.010	
Condiments and sauces	0.002	0.002		-0.013	0.005	*	0.000	0.001		-0.002	0.001		0.001	0.002		0.000	0.001		0.001	0.010	
Low calorie, soft drinks and juices	-0.011	0.004	*	-0.012	0.009		0.000	0.002		0.001	0.002		0.002	0.005		0.005	0.003	*	-0.001	0.019	
Alcoholic beverages	0.000	0.002		0.003	0.005		-0.002	0.001	*	0.000	0.001		0.005	0.003	*	-0.006	0.008		0.006	0.014	
Numeraire category	-0.005	-0.078		0.000	-0.212		-0.002	-0.038		0.001	-0.038		-0.001	-0.070		0.006	-0.049		0.017	0.436	
**Total expenditure**	-0.016	0.001	*	-0.005	0.003	*	-0.003	0.001	*	-0.004	0.001	*	0.002	0.001		0.011	0.005	*	0.046	0.008	*
**Promotions**																					
Take home confectionery	0.000	0.000		-0.002	0.001	*	0.000	0.000		0.000	0.000		0.002	0.001	*	-0.003	0.002	*	-0.001	0.003	
Biscuits	0.001	0.000	*	0.002	0.001	*	0.000	0.000	*	0.000	0.000		0.000	0.001		-0.003	0.002	*	-0.001	0.003	
Take home savouries	-0.001	0.000	*	0.000	0.001		0.000	0.000		0.000	0.000		-0.001	0.000		0.001	0.001		-0.001	0.002	
Cakes, pastries, and sugar morning goods	0.000	0.000		-0.001	0.001		0.000	0.000		0.000	0.000		-0.001	0.000		-0.001	0.001		0.000	0.002	
Total puddings and desserts	-0.001	0.000		0.002	0.001	*	0.000	0.000		-0.001	0.000	*	0.001	0.000	*	-0.003	0.002	*	0.000	0.002	
Take home sugary drinks	0.000	0.000		0.000	0.000		0.000	0.000		0.000	0.000	*	-0.001	0.000	*	0.000	0.001		0.000	0.001	
Edible ices and ice creams	0.000	0.000		0.001	0.001		0.000	0.000	*	0.000	0.000		0.000	0.000		0.001	0.001		-0.001	0.002	
Dairy products	0.001	0.000	*	-0.001	0.000	*	0.000	0.000		0.000	0.000		0.000	0.000		0.000	0.001		-0.001	0.001	
Meat and fish	-0.001	0.000	*	-0.002	0.001	*	0.000	0.000		0.000	0.000		0.001	0.001		0.000	0.002		-0.003	0.003	
Fats and eggs	0.000	0.000		0.000	0.001		0.000	0.000		0.000	0.000		0.000	0.000		-0.001	0.001		-0.001	0.002	
Fruit	0.000	0.000		0.001	0.001	*	0.000	0.000		0.000	0.000	*	-0.001	0.000	*	-0.002	0.001	*	-0.001	0.002	
Vegetables	0.001	0.001		-0.003	0.001	*	0.000	0.000		0.001	0.000	*	-0.001	0.001		-0.004	0.002	*	-0.003	0.004	
Grains	0.002	0.000	*	-0.002	0.001	*	0.000	0.000		0.000	0.000		0.000	0.000		-0.001	0.001		0.000	0.002	
Prepared ready to eat foods	-0.001	0.000		0.015	0.001	*	-0.001	0.000	*	0.000	0.000		-0.001	0.001		-0.006	0.002	*	0.000	0.003	
Sugar and preserves	0.001	0.001		0.000	0.002		0.003	0.000	*	-0.001	0.000		0.001	0.001		-0.009	0.003	*	0.004	0.005	
Condiments and sauces	0.003	0.001	*	0.003	0.002	*	-0.001	0.000	*	0.004	0.000	*	-0.001	0.001		-0.007	0.003	*	0.002	0.005	
Low calorie, soft drinks and juices	0.000	0.000		0.000	0.000		0.000	0.000		0.000	0.000		0.003	0.000	*	0.000	0.001		-0.001	0.001	
Alcoholic beverages	-0.002	0.000	*	-0.003	0.001	*	0.000	0.000	*	-0.001	0.000	*	-0.002	0.000	*	0.024	0.002	*	-0.001	0.003	
Numeraire category	0.001	0.001		-0.002	0.001		-0.001	0.000	*	0.000	0.000		-0.003	0.001	*	0.004	0.003	*	0.019	0.004	*

Note: '*' indicates statistically significant at 5 per cent.

Table S5 - Scotland - £50,000 - £59,999 - All categories - Demand model results

Equations (part 1)

Variables	Take home confectionery			Biscuits			Take home savouries			Cakes, pastries and sugar morning goods			Total puddings and desserts		
	Coef.	St. Err.	Sig.	Coef.	St. Err.	Sig.	Coef.	St. Err.	Sig.	Coef.	St. Err.	Sig.	Coef.	St. Err.	Sig.
**Intercept**	0.038	0.014	*	-0.010	0.017		0.052	0.018	*	0.017	0.018		0.009	0.011	
**Prices**															
Take home confectionery	-0.008	0.005	*	0.004	0.003		-0.007	0.003	*	0.015	0.003	*	-0.002	0.002	
Biscuits	0.004	0.003		0.003	0.008		0.007	0.007		0.001	0.005		-0.004	0.004	
Take home savouries	-0.007	0.003	*	0.007	0.007		-0.003	0.010		0.006	0.005		-0.001	0.004	
Cakes, pastries, and sugar morning goods	0.015	0.003	*	0.001	0.005		0.006	0.005		-0.006	0.007		-0.001	0.003	
Total puddings and desserts	-0.002	0.002		-0.004	0.004		-0.001	0.004		-0.001	0.003		-0.004	0.003	
Take home sugary drinks	-0.004	0.004		-0.009	0.005	*	0.009	0.005	*	0.006	0.005		0.000	0.003	
Edible ices and ice creams	0.000	0.002		0.002	0.004		-0.006	0.005		0.001	0.003		-0.001	0.003	
Dairy products	0.002	0.006		-0.004	0.008		-0.007	0.008		-0.008	0.008		-0.009	0.005	*
Meat and fish	-0.012	0.010		0.015	0.012		-0.005	0.012		-0.002	0.013		0.006	0.007	
Fats and eggs	-0.012	0.003	*	-0.002	0.004		0.002	0.005		0.007	0.004	*	-0.002	0.003	
Fruit	0.014	0.006	*	-0.008	0.007		0.001	0.008		-0.013	0.007	*	0.003	0.004	
Vegetables	0.016	0.006	*	0.003	0.006		0.001	0.007		-0.005	0.007		-0.002	0.004	
Grains	0.002	0.004		0.000	0.004		0.008	0.005	*	0.008	0.005	*	0.002	0.003	
Prepared ready to eat foods	-0.003	0.010		-0.007	0.013		0.004	0.013		-0.014	0.013		0.015	0.008	*
Sugar and preserves	0.001	0.001		0.003	0.002		0.003	0.002		0.005	0.002	*	-0.002	0.001	
Condiments and sauces	0.001	0.002		-0.001	0.002		-0.006	0.002	*	0.003	0.002		-0.001	0.001	
Low calorie, soft drinks and juices	-0.008	0.004	*	0.005	0.003		-0.002	0.004		0.001	0.004		0.000	0.002	
Alcoholic beverages	-0.001	0.003		-0.005	0.002	*	-0.005	0.002	*	0.000	0.002		0.002	0.001	*
Numeraire category	0.002	-0.076		-0.004	-0.099		0.000	-0.106		-0.004	-0.096		0.000	-0.059	
**Total expenditure**	0.000	0.002		-0.005	0.001	*	-0.007	0.001	*	-0.002	0.001	*	0.000	0.001	
**Promotions**															
Take home confectionery	0.006	0.001	*	0.001	0.000		-0.001	0.000		0.000	0.000		0.000	0.000	
Biscuits	0.001	0.001		0.003	0.000	*	0.001	0.000		0.001	0.000		0.000	0.000	
Take home savouries	0.001	0.001		0.000	0.000		0.003	0.000	*	0.000	0.000		0.000	0.000	
Cakes, pastries, and sugar morning goods	0.000	0.001		0.000	0.000		0.001	0.000	*	0.003	0.000	*	0.000	0.000	
Total puddings and desserts	0.001	0.001		0.001	0.000	*	0.001	0.000	*	0.001	0.000		0.002	0.000	*
Take home sugary drinks	0.001	0.000	*	0.000	0.000		0.000	0.000		0.001	0.000	*	0.000	0.000	
Edible ices and ice creams	0.002	0.001	*	0.000	0.000		0.000	0.000		0.001	0.000		0.000	0.000	
Dairy products	0.001	0.000	*	0.000	0.000	*	0.000	0.000		0.000	0.000		0.000	0.000	
Meat and fish	-0.002	0.001	*	0.000	0.000		0.000	0.000		-0.002	0.001	*	0.000	0.000	
Fats and eggs	0.000	0.000		0.000	0.000		0.000	0.000		0.000	0.000		0.001	0.000	*
Fruit	0.000	0.001		0.001	0.000		0.000	0.000		-0.001	0.000		0.000	0.000	
Vegetables	-0.002	0.001	*	-0.002	0.000	*	-0.002	0.000	*	0.000	0.001		-0.001	0.000	*
Grains	0.000	0.001		0.000	0.000		0.000	0.000		0.000	0.000		0.000	0.000	
Prepared ready to eat foods	0.001	0.001		0.001	0.001		-0.001	0.001		0.000	0.001		0.000	0.000	
Sugar and preserves	0.002	0.001	*	0.000	0.001		-0.001	0.001	*	0.002	0.001	*	-0.001	0.000	*
Condiments and sauces	0.001	0.001		-0.001	0.001		0.000	0.001		0.001	0.001		-0.001	0.000	
Low calorie, soft drinks and juices	0.000	0.000		0.000	0.000	*	0.000	0.000	*	0.000	0.000		0.000	0.000	
Alcoholic beverages	-0.002	0.001	*	-0.001	0.000	*	-0.001	0.000	*	-0.002	0.000	*	-0.001	0.000	*
Numeraire category	-0.001	0.001		0.000	0.000		0.000	0.000		0.000	0.000		0.000	0.000	
Log-Likelihood	29354.600														
Obs.	619.000														

Equations (part 2)

Variables	Take home sugary drinks			Edible ices and ice cream			Dairy products			Meat and fish			Fats and eggs		
	Coef.	St. Err.	Sig.	Coef.	St. Err.	Sig.	Coef.	St. Err.	Sig.	Coef.	St. Err.	Sig.	Coef.	St. Err.	Sig.
**Intercept**	0.004	0.020		0.011	0.012		0.130	0.031	*	0.010	0.054		0.034	0.014	*
**Prices**															
Take home confectionery	-0.004	0.004		0.000	0.002		0.002	0.006		-0.012	0.010		-0.012	0.003	*
Biscuits	-0.009	0.005	*	0.002	0.004		-0.004	0.008		0.015	0.012		-0.002	0.004	
Take home savouries	0.009	0.005	*	-0.006	0.005		-0.007	0.008		-0.005	0.012		0.002	0.005	
Cakes, pastries, and sugar morning goods	0.006	0.005		0.001	0.003		-0.008	0.008		-0.002	0.013		0.007	0.004	*
Total puddings and desserts	0.000	0.003		-0.001	0.003		-0.009	0.005	*	0.006	0.007		-0.002	0.003	
Take home sugary drinks	-0.006	0.007		0.000	0.003		0.009	0.008		0.003	0.013		-0.002	0.004	
Edible ices and ice creams	0.000	0.003		0.005	0.004		-0.004	0.005		-0.010	0.008		0.003	0.003	
Dairy products	0.009	0.008		-0.004	0.005		0.009	0.018		0.011	0.021		0.001	0.006	
Meat and fish	0.003	0.013		-0.010	0.008		0.011	0.021		0.100	0.048	*	0.008	0.009	
Fats and eggs	-0.002	0.004		0.003	0.003		0.001	0.006		0.008	0.009		0.007	0.005	
Fruit	-0.006	0.008		0.006	0.005		0.011	0.013		-0.054	0.021	*	0.010	0.006	
Vegetables	0.002	0.007		-0.005	0.004		-0.007	0.011		-0.033	0.019	*	0.014	0.005	*
Grains	-0.006	0.005		-0.001	0.003		0.004	0.008		-0.021	0.014		-0.004	0.004	
Prepared ready to eat foods	-0.006	0.013		0.015	0.008	*	-0.017	0.022		-0.005	0.034		-0.037	0.010	*
Sugar and preserves	0.003	0.002		-0.002	0.001		0.001	0.003		-0.002	0.005		-0.002	0.002	
Condiments and sauces	0.000	0.002		0.000	0.001		0.000	0.004		0.002	0.006		0.002	0.002	
Low calorie, soft drinks and juices	0.009	0.004	*	0.001	0.002		0.005	0.007		0.018	0.012		0.007	0.003	*
Alcoholic beverages	0.000	0.003		-0.001	0.001		-0.005	0.005		0.010	0.008		0.000	0.002	
Numeraire category	-0.001	-0.102		-0.001	-0.067		0.007	-0.164		-0.027	-0.272		0.000	-0.079	
**Total expenditure**	0.003	0.001	*	0.000	0.001		-0.010	0.002	*	-0.010	0.004	*	-0.001	0.001	
**Promotions**															
Take home confectionery	0.000	0.001		0.000	0.000		-0.002	0.001	*	0.001	0.002		0.000	0.000	
Biscuits	0.002	0.001	*	0.000	0.000		-0.001	0.001		-0.002	0.002		-0.001	0.000	*
Take home savouries	0.001	0.001	*	0.000	0.000		-0.001	0.001		0.003	0.002	*	0.000	0.000	
Cakes, pastries, and sugar morning goods	0.000	0.000		0.000	0.000		0.000	0.001		-0.001	0.001		-0.001	0.000	*
Total puddings and desserts	0.000	0.000		0.000	0.000		0.001	0.001		-0.005	0.001	*	0.000	0.000	
Take home sugary drinks	0.003	0.000	*	0.000	0.000		-0.001	0.000	*	0.001	0.001	*	0.000	0.000	
Edible ices and ice creams	-0.001	0.001		0.004	0.000	*	-0.003	0.001	*	-0.001	0.002		0.000	0.000	
Dairy products	0.002	0.000	*	0.000	0.000		0.003	0.001	*	-0.001	0.001		0.000	0.000	
Meat and fish	0.000	0.001		0.000	0.000		0.000	0.001		0.007	0.002	*	0.000	0.000	
Fats and eggs	0.000	0.000		0.000	0.000		0.001	0.001		0.000	0.001		0.001	0.000	*
Fruit	-0.001	0.001		0.000	0.000		0.003	0.001	*	-0.001	0.002		0.000	0.000	
Vegetables	-0.002	0.001	*	0.000	0.000		0.001	0.001		-0.001	0.002		0.001	0.000	*
Grains	-0.002	0.001	*	0.000	0.000		-0.001	0.001		0.000	0.001		0.000	0.000	
Prepared ready to eat foods	0.001	0.001		0.000	0.000		-0.001	0.001		-0.004	0.002	*	0.000	0.000	
Sugar and preserves	-0.001	0.001	*	0.000	0.000		-0.001	0.001		-0.003	0.002		0.002	0.000	*
Condiments and sauces	0.000	0.001		0.000	0.001		-0.001	0.002		-0.005	0.004		-0.001	0.001	
Low calorie, soft drinks and juices	0.000	0.000	*	0.000	0.000		0.000	0.000		0.000	0.000		0.000	0.000	*
Alcoholic beverages	-0.001	0.000	*	0.000	0.000		-0.004	0.001	*	0.000	0.001		0.000	0.000	
Numeraire category	-0.001	0.001		0.000	0.000		-0.001	0.001		0.000	0.001		-0.001	0.000	*

Equations (part 3)

Variables	Fruit			Vegetables			Grains			Prepared ready to eat foods			Sugar and preserves		
	Coef.	St. Err.	Sig.	Coef.	St. Err.	Sig.	Coef.	St. Err.	Sig.	Coef.	St. Err.	Sig.	Coef.	St. Err.	Sig.
**Intercept**	0.108	0.030	*	0.117	0.027	*	0.077	0.019	*	0.030	0.049		0.027	0.007	*
**Prices**															
Take home confectionery	0.014	0.006	*	0.016	0.006	*	0.002	0.004		-0.003	0.010		0.001	0.001	
Biscuits	-0.008	0.007		0.003	0.006		0.000	0.004		-0.007	0.013		0.003	0.002	
Take home savouries	0.001	0.008		0.001	0.007		0.008	0.005	*	0.004	0.013		0.003	0.002	
Cakes, pastries, and sugar morning goods	-0.013	0.007	*	-0.005	0.007		0.008	0.005	*	-0.014	0.013		0.005	0.002	*
Total puddings and desserts	0.003	0.004		-0.002	0.004		0.002	0.003		0.015	0.008	*	-0.002	0.001	
Take home sugary drinks	-0.006	0.008		0.002	0.007		-0.006	0.005		-0.006	0.013		0.003	0.002	
Edible ices and ice creams	0.006	0.005		-0.005	0.004		-0.001	0.003		0.015	0.008	*	-0.002	0.001	
Dairy products	0.011	0.013		-0.007	0.011		0.004	0.008		-0.017	0.022		0.001	0.003	
Meat and fish	-0.054	0.021	*	-0.033	0.019	*	-0.021	0.014		-0.005	0.034		-0.002	0.005	
Fats and eggs	0.010	0.006		0.014	0.005	*	-0.004	0.004		-0.037	0.010	*	-0.002	0.002	
Fruit	-0.029	0.018		0.013	0.012		0.010	0.008		0.041	0.021	*	0.007	0.003	*
Vegetables	0.013	0.012		-0.004	0.014		-0.004	0.007		0.012	0.019		-0.001	0.003	
Grains	0.010	0.008		-0.004	0.007		-0.002	0.007		0.009	0.013		-0.005	0.002	*
Prepared ready to eat foods	0.041	0.021	*	0.012	0.019		0.009	0.013		0.015	0.048		-0.009	0.005	*
Sugar and preserves	0.007	0.003	*	-0.001	0.003		-0.005	0.002	*	-0.009	0.005	*	-0.002	0.001	*
Condiments and sauces	-0.005	0.004		0.003	0.003		-0.001	0.002		0.011	0.006	*	0.000	0.001	
Low calorie, soft drinks and juices	-0.003	0.007		-0.006	0.007		0.002	0.005		-0.025	0.011	*	0.005	0.002	*
Alcoholic beverages	-0.002	0.006		0.000	0.005		-0.004	0.003		-0.001	0.007		-0.001	0.001	
Numeraire category	0.005	-0.166		0.004	-0.146		0.004	-0.102		0.003	-0.274		-0.001	-0.039	
**Total expenditure**	-0.008	0.003	*	-0.004	0.002	*	-0.011	0.002	*	-0.005	0.004		-0.003	0.000	*
**Promotions**															
Take home confectionery	-0.001	0.001		-0.003	0.001	*	-0.001	0.001	*	-0.003	0.001	*	0.000	0.000	
Biscuits	0.002	0.001		-0.001	0.001		-0.001	0.001		0.001	0.001		0.000	0.000	
Take home savouries	-0.002	0.001	*	-0.003	0.001	*	0.000	0.001		0.001	0.001		0.000	0.000	
Cakes, pastries, and sugar morning goods	0.001	0.001		0.000	0.001		-0.001	0.001	*	-0.001	0.001		0.000	0.000	
Total puddings and desserts	0.000	0.001		0.000	0.001		0.001	0.001		0.000	0.001		0.000	0.000	
Take home sugary drinks	-0.001	0.000	*	-0.002	0.000	*	-0.001	0.000	*	0.002	0.001	*	0.000	0.000	
Edible ices and ice creams	-0.001	0.001		0.000	0.001		-0.001	0.001	*	0.000	0.001		0.000	0.000	*
Dairy products	0.001	0.001		0.000	0.001		0.000	0.000		-0.002	0.001	*	0.000	0.000	
Meat and fish	-0.003	0.001	*	-0.003	0.001	*	0.001	0.001		-0.005	0.002	*	0.000	0.000	
Fats and eggs	0.000	0.001		0.000	0.001		0.001	0.000	*	0.002	0.001	*	0.000	0.000	
Fruit	0.009	0.001	*	0.002	0.001	*	0.000	0.001		0.000	0.001		0.000	0.000	
Vegetables	0.002	0.001	*	0.007	0.001	*	0.000	0.001		-0.009	0.002	*	0.000	0.000	
Grains	-0.001	0.001		-0.001	0.001		0.003	0.001	*	0.002	0.001		0.000	0.000	
Prepared ready to eat foods	-0.003	0.002		-0.002	0.001		0.001	0.001		0.013	0.002	*	0.000	0.000	
Sugar and preserves	0.003	0.002	*	0.000	0.001		0.001	0.001		0.001	0.002		0.004	0.000	*
Condiments and sauces	-0.003	0.003		-0.002	0.002		0.002	0.001	*	0.007	0.003	*	0.000	0.000	
Low calorie, soft drinks and juices	-0.001	0.000	*	0.000	0.000		0.000	0.000		0.001	0.000	*	0.000	0.000	
Alcoholic beverages	-0.002	0.001	*	-0.001	0.001		-0.002	0.001	*	-0.004	0.001	*	0.000	0.000	*
Numeraire category	-0.002	0.001	*	-0.001	0.001		0.000	0.001		0.000	0.001		0.000	0.000	

Equations (part 4)

Variables	Condiments and sauces			Low calorie soft drinks and juices			Alcoholic beverages			Numeraire category		
	Coef.	St. Err.	Sig.	Coef.	St. Err.	Sig.	Coef.	St. Err.	Sig.	Coef.	St. Err.	Sig.
**Intercept**	0.031	0.008	*	0.059	0.016	*	0.122	0.020	*	0.134	0.105	
**Prices**												
Take home confectionery	0.001	0.002		-0.008	0.004	*	-0.001	0.003		0.002	0.021	
Biscuits	-0.001	0.002		0.005	0.003		-0.005	0.002	*	-0.004	0.027	
Take home savouries	-0.006	0.002	*	-0.002	0.004		-0.005	0.002	*	0.000	0.029	
Cakes, pastries, and sugar morning goods	0.003	0.002		0.001	0.004		0.000	0.002		-0.004	0.026	
Total puddings and desserts	-0.001	0.001		0.000	0.002		0.002	0.001	*	0.000	0.016	
Take home sugary drinks	0.000	0.002		0.009	0.004	*	0.000	0.003		-0.001	0.028	
Edible ices and ice creams	0.000	0.001		0.001	0.002		-0.001	0.001		-0.001	0.018	
Dairy products	0.000	0.004		0.005	0.007		-0.005	0.005		0.007	0.045	
Meat and fish	0.002	0.006		0.018	0.012		0.010	0.008		-0.027	0.078	
Fats and eggs	0.002	0.002		0.007	0.003	*	0.000	0.002		0.000	0.021	
Fruit	-0.005	0.004		-0.003	0.007		-0.002	0.006		0.005	0.046	
Vegetables	0.003	0.003		-0.006	0.007		0.000	0.005		0.004	0.040	
Grains	-0.001	0.002		0.002	0.005		-0.004	0.003		0.004	0.028	
Prepared ready to eat foods	0.011	0.006	*	-0.025	0.011	*	-0.001	0.007		0.003	0.078	
Sugar and preserves	0.000	0.001		0.005	0.002	*	-0.001	0.001		-0.001	0.011	
Condiments and sauces	-0.003	0.001	*	-0.003	0.002		0.001	0.001		-0.003	0.012	
Low calorie, soft drinks and juices	-0.003	0.002		-0.009	0.007		-0.003	0.004		0.005	0.024	
Alcoholic beverages	0.001	0.001		-0.003	0.004		-0.004	0.013		0.018	0.021	
Numeraire category	-0.003	-0.044		0.005	-0.089		0.018	-0.071		-0.007	0.559	
**Total expenditure**	-0.002	0.001	*	-0.001	0.002		0.032	0.007	*	0.036	0.011	*
**Promotions**												
Take home confectionery	-0.001	0.000	*	-0.003	0.001	*	0.011	0.003	*	-0.003	0.004	
Biscuits	-0.001	0.000	*	-0.001	0.001		-0.001	0.003		-0.002	0.004	
Take home savouries	0.000	0.000	*	0.002	0.001	*	-0.005	0.003	*	0.000	0.004	
Cakes, pastries, and sugar morning goods	0.000	0.000		-0.001	0.001		0.001	0.002		-0.002	0.004	
Total puddings and desserts	0.000	0.000	*	0.001	0.001	*	-0.004	0.002		0.001	0.004	
Take home sugary drinks	0.000	0.000	*	0.000	0.000		-0.001	0.001		0.000	0.002	
Edible ices and ice creams	0.000	0.000		0.002	0.001	*	0.001	0.003		-0.002	0.004	
Dairy products	0.000	0.000		-0.001	0.000		-0.003	0.001	*	-0.001	0.002	
Meat and fish	-0.001	0.000	*	0.001	0.001	*	0.002	0.003		0.004	0.005	
Fats and eggs	0.000	0.000		0.000	0.001		-0.002	0.002		-0.003	0.003	
Fruit	0.000	0.000		0.000	0.001		-0.011	0.003	*	0.000	0.004	
Vegetables	0.000	0.000		-0.001	0.001		0.009	0.003	*	-0.002	0.005	
Grains	0.000	0.000		0.000	0.001		-0.003	0.002		0.003	0.004	
Prepared ready to eat foods	0.000	0.000		0.000	0.001		-0.005	0.004		0.001	0.006	
Sugar and preserves	0.000	0.000		-0.001	0.001		-0.010	0.004	*	0.004	0.006	
Condiments and sauces	0.006	0.001	*	-0.001	0.002		-0.004	0.006		0.001	0.009	
Low calorie, soft drinks and juices	0.000	0.000		0.002	0.000	*	0.000	0.001		0.000	0.001	
Alcoholic beverages	0.000	0.000		-0.002	0.001	*	0.027	0.002	*	-0.004	0.004	
Numeraire category	0.000	0.000		-0.001	0.001		0.001	0.002		0.007	0.004	*

Note: '*' indicates statistically significant at 5 per cent.

Table S6 - Scotland - £60,000 - over income group - All categories - Demand model results

Variables	Equations														
	Take home confectionery			Biscuits			Take home savouries			Cakes, pastries and sugar morning goods			Total puddings and desserts		
	Coef.	St. Err.	Sig.	Coef.	St. Err.	Sig.	Coef.	St. Err.	Sig.	Coef.	St. Err.	Sig.	Coef.	St. Err.	Sig.
**Intercept**	0.054	0.016	*	0.023	0.018		0.055	0.020	*	0.001	0.019		0.023	0.012	*
**Prices**															
Take home confectionery	-0.003	0.005		-0.005	0.003		-0.006	0.004		-0.001	0.004		-0.004	0.002	
Biscuits	-0.005	0.003		0.006	0.009		-0.007	0.008		0.001	0.005		-0.004	0.004	
Take home savouries	-0.006	0.004		-0.007	0.008		0.022	0.011	*	0.010	0.006	*	-0.003	0.005	
Cakes, pastries, and sugar morning goods	-0.001	0.004		0.001	0.005		0.010	0.006	*	0.008	0.007		0.004	0.003	
Total puddings and desserts	-0.004	0.002		-0.004	0.004		-0.003	0.005		0.004	0.003		0.002	0.004	
Take home sugary drinks	0.007	0.004	*	0.002	0.005		0.008	0.005		-0.005	0.005		0.004	0.003	
Edible ices and ice creams	0.007	0.003	*	-0.002	0.005		-0.007	0.006		-0.001	0.004		0.001	0.003	
Dairy products	0.000	0.007		0.005	0.007		0.015	0.009	*	-0.007	0.008		0.002	0.005	
Meat and fish	0.013	0.011		0.024	0.012	*	-0.001	0.013		0.020	0.013		0.003	0.008	
Fats and eggs	0.004	0.003		0.006	0.005		-0.006	0.005		-0.006	0.004		0.000	0.003	
Fruit	0.006	0.007		-0.010	0.007		-0.015	0.008	*	-0.012	0.008		-0.001	0.005	
Vegetables	-0.005	0.006		-0.010	0.007		0.006	0.008		-0.002	0.007		0.001	0.004	
Grains	-0.005	0.004		0.002	0.005		0.001	0.005		0.002	0.005		-0.005	0.003	
Prepared ready to eat foods	-0.018	0.010	*	-0.014	0.013		-0.019	0.015		-0.013	0.012		-0.007	0.008	
Sugar and preserves	-0.002	0.002		0.000	0.002		0.003	0.003		0.000	0.002		-0.001	0.001	
Condiments and sauces	-0.003	0.002		-0.001	0.002		-0.006	0.002	*	-0.002	0.002		0.001	0.001	
Low calorie, soft drinks and juices	0.007	0.004	*	0.008	0.004	*	0.002	0.004		0.001	0.004		0.005	0.002	*
Alcoholic beverages	0.005	0.003	*	-0.001	0.002		0.000	0.002		0.001	0.002		0.000	0.001	
Numeraire category	0.003	-0.084		0.000	-0.103		0.002	-0.119		0.000	-0.101		0.001	-0.066	
**Total expenditure**	0.003	0.002		-0.002	0.001	*	-0.004	0.001	*	-0.005	0.001	*	-0.001	0.001	
**Promotions**															
Take home confectionery	0.004	0.001	*	0.001	0.000	*	0.001	0.000		0.001	0.001	*	0.000	0.000	
Biscuits	0.000	0.001		0.002	0.000	*	0.000	0.000		0.000	0.001		0.000	0.000	
Take home savouries	0.001	0.000	*	0.000	0.000		0.004	0.000	*	0.000	0.000		0.000	0.000	
Cakes, pastries, and sugar morning goods	0.000	0.001		0.000	0.000		0.000	0.000		0.003	0.000	*	0.000	0.000	
Total puddings and desserts	0.000	0.001		0.001	0.000	*	0.000	0.000		0.000	0.000		0.001	0.000	*
Take home sugary drinks	0.000	0.000		0.000	0.000	*	0.000	0.000		0.000	0.000		0.000	0.000	*
Edible ices and ice creams	0.001	0.000	*	0.001	0.000	*	0.000	0.000		0.001	0.000		0.000	0.000	
Dairy products	0.000	0.000		0.000	0.000	*	0.000	0.000		0.000	0.000	*	0.000	0.000	
Meat and fish	0.000	0.001		0.000	0.000		0.001	0.000	*	0.000	0.000		-0.001	0.000	*
Fats and eggs	-0.002	0.000	*	0.000	0.000		-0.001	0.000	*	0.000	0.000		0.000	0.000	
Fruit	0.000	0.001		0.000	0.000		0.000	0.000		0.000	0.000		0.000	0.000	
Vegetables	-0.001	0.001		-0.001	0.000	*	-0.001	0.001	*	0.000	0.001		0.000	0.000	
Grains	0.001	0.001	*	0.001	0.000	*	-0.001	0.000		0.000	0.000		0.000	0.000	
Prepared ready to eat foods	0.000	0.001		0.000	0.001		0.001	0.001		0.002	0.001	*	0.000	0.000	
Sugar and preserves	0.000	0.001		0.000	0.001		-0.001	0.001		-0.001	0.001		0.001	0.000	*
Condiments and sauces	0.002	0.001	*	0.001	0.001		0.001	0.001		0.000	0.001		0.000	0.000	
Low calorie, soft drinks and juices	0.000	0.000		0.000	0.000		0.000	0.000		0.000	0.000	*	0.000	0.000	
Alcoholic beverages	-0.002	0.001	*	-0.002	0.000	*	-0.002	0.000	*	-0.002	0.000	*	-0.001	0.000	*
Numeraire category	-0.002	0.001	*	-0.002	0.001	*	0.000	0.001		-0.002	0.001	*	-0.001	0.000	*
Log-Likelihood	26795.200														
Obs.	564.000														

Variables	Equations														
	Take home sugary drinks			Edible ices and ice cream			Dairy products			Meat and fish			Fats and eggs		
	Coef.	St. Err.	Sig.	Coef.	St. Err.	Sig.	Coef.	St. Err.	Sig.	Coef.	St. Err.	Sig.	Coef.	St. Err.	Sig.
**Intercept**	-0.008	0.018		0.025	0.014	*	0.052	0.033		0.228	0.053	*	0.021	0.015	
**Prices**															
Take home confectionery	0.007	0.004	*	0.007	0.003	*	0.000	0.007		0.013	0.011		0.004	0.003	
Biscuits	0.002	0.005		-0.002	0.005		0.005	0.007		0.024	0.012	*	0.006	0.005	
Take home savouries	0.008	0.005		-0.007	0.006		0.015	0.009	*	-0.001	0.013		-0.006	0.005	
Cakes, pastries, and sugar morning goods	-0.005	0.005		-0.001	0.004		-0.007	0.008		0.020	0.013		-0.006	0.004	
Total puddings and desserts	0.004	0.003		0.001	0.003		0.002	0.005		0.003	0.008		0.000	0.003	
Take home sugary drinks	-0.002	0.006		-0.002	0.004		0.007	0.008		0.007	0.012		0.004	0.004	
Edible ices and ice creams	-0.002	0.004		-0.002	0.006		0.006	0.006		-0.006	0.009		0.004	0.004	
Dairy products	0.007	0.008		0.006	0.006		-0.002	0.019		-0.045	0.022	*	0.002	0.006	
Meat and fish	0.007	0.012		-0.006	0.009		-0.045	0.022	*	-0.131	0.046	*	0.013	0.010	
Fats and eggs	0.004	0.004		0.004	0.004		0.002	0.006		0.013	0.010		0.006	0.005	
Fruit	-0.012	0.007	*	-0.005	0.005		-0.014	0.013		0.024	0.021		-0.014	0.006	*
Vegetables	-0.003	0.007		0.001	0.005		-0.030	0.012	*	0.041	0.018	*	-0.010	0.006	*
Grains	-0.002	0.005		-0.001	0.004		0.005	0.009		0.016	0.013		0.004	0.004	
Prepared ready to eat foods	-0.010	0.012		0.008	0.010		0.074	0.021	*	0.019	0.032		0.002	0.010	
Sugar and preserves	0.001	0.002		0.000	0.002		-0.003	0.004		0.010	0.007		0.002	0.002	
Condiments and sauces	0.004	0.002	*	-0.002	0.002		0.000	0.004		0.001	0.006		-0.003	0.002	*
Low calorie, soft drinks and juices	-0.005	0.004		0.002	0.003		0.010	0.007		-0.013	0.012		-0.005	0.003	
Alcoholic beverages	-0.001	0.002		0.001	0.001		-0.009	0.005	*	0.003	0.007		-0.001	0.002	
Numeraire category	-0.002	-0.095		-0.001	-0.081		-0.016	-0.172		0.003	-0.272		-0.002	-0.082	
**Total expenditure**	-0.002	0.001		-0.002	0.001	*	-0.008	0.003	*	-0.007	0.004		-0.002	0.001	*
**Promotions**															
Take home confectionery	0.000	0.000		0.001	0.000	*	0.000	0.001		-0.002	0.002		-0.001	0.000	
Biscuits	-0.001	0.000		0.000	0.000		0.000	0.001		0.000	0.002		0.000	0.000	
Take home savouries	0.001	0.000		0.000	0.000		-0.003	0.001	*	-0.001	0.001		-0.001	0.000	*
Cakes, pastries, and sugar morning goods	0.000	0.000		0.000	0.000		-0.002	0.001	*	0.007	0.001	*	0.000	0.000	
Total puddings and desserts	0.001	0.000	*	0.000	0.000		0.000	0.001		-0.001	0.001		0.000	0.000	
Take home sugary drinks	0.003	0.000	*	0.000	0.000		-0.001	0.001	*	0.001	0.001		0.000	0.000	
Edible ices and ice creams	0.000	0.000		0.002	0.000	*	0.000	0.001		0.001	0.001		0.000	0.000	
Dairy products	0.000	0.000		0.000	0.000		0.003	0.001	*	-0.003	0.001	*	0.000	0.000	
Meat and fish	0.000	0.000		-0.001	0.000	*	-0.002	0.001		0.004	0.002	*	0.000	0.000	
Fats and eggs	0.001	0.000		0.000	0.000		0.000	0.001		0.004	0.001	*	0.001	0.000	*
Fruit	-0.001	0.000	*	0.000	0.000		0.002	0.001	*	-0.001	0.001		0.000	0.000	
Vegetables	-0.001	0.001	*	-0.001	0.000	*	0.001	0.001		0.004	0.002	*	0.000	0.000	
Grains	-0.001	0.000	*	0.000	0.000		0.003	0.001	*	-0.004	0.002	*	0.000	0.000	
Prepared ready to eat foods	-0.002	0.001	*	0.000	0.000		-0.003	0.002	*	-0.003	0.003		-0.001	0.001	*
Sugar and preserves	0.003	0.001	*	0.001	0.001	*	-0.001	0.002		0.002	0.003		-0.001	0.001	
Condiments and sauces	0.000	0.001		0.000	0.000		0.001	0.002		0.000	0.002		0.000	0.001	
Low calorie, soft drinks and juices	0.000	0.000		0.000	0.000	*	0.000	0.000		0.000	0.001		0.000	0.000	*
Alcoholic beverages	-0.001	0.000	*	0.000	0.000		-0.003	0.001	*	0.003	0.001	*	0.000	0.000	
Numeraire category	-0.001	0.001	*	-0.001	0.000	*	0.001	0.001		-0.003	0.002	*	0.001	0.000	*

Variables	Equations														
	Fruit			Vegetables			Grains			Prepared ready to eat foods			Sugar and preserves		
	Coef.	St. Err.	Sig.	Coef.	St. Err.	Sig.	Coef.	St. Err.	Sig.	Coef.	St. Err.	Sig.	Coef.	St. Err.	Sig.
**Intercept**	0.013	0.030		0.020	0.027		0.043	0.019	*	0.117	0.047	*	-0.001	0.010	
**Prices**															
Take home confectionery	0.006	0.007		-0.005	0.006		-0.005	0.004		-0.018	0.010	*	-0.002	0.002	
Biscuits	-0.010	0.007		-0.010	0.007		0.002	0.005		-0.014	0.013		0.000	0.002	
Take home savouries	-0.015	0.008	*	0.006	0.008		0.001	0.005		-0.019	0.015		0.003	0.003	
Cakes, pastries, and sugar morning goods	-0.012	0.008		-0.002	0.007		0.002	0.005		-0.013	0.012		0.000	0.002	
Total puddings and desserts	-0.001	0.005		0.001	0.004		-0.005	0.003		-0.007	0.008		-0.001	0.001	
Take home sugary drinks	-0.012	0.007	*	-0.003	0.007		-0.002	0.005		-0.010	0.012		0.001	0.002	
Edible ices and ice creams	-0.005	0.005		0.001	0.005		-0.001	0.004		0.008	0.010		0.000	0.002	
Dairy products	-0.014	0.013		-0.030	0.012	*	0.005	0.009		0.074	0.021	*	-0.003	0.004	
Meat and fish	0.024	0.021		0.041	0.018	*	0.016	0.013		0.019	0.032		0.010	0.007	
Fats and eggs	-0.014	0.006	*	-0.010	0.006	*	0.004	0.004		0.002	0.010		0.002	0.002	
Fruit	0.029	0.018		-0.024	0.011	*	-0.002	0.008		0.054	0.020	*	0.000	0.004	
Vegetables	-0.024	0.011	*	0.003	0.014		0.020	0.007	*	0.026	0.018		-0.006	0.004	*
Grains	-0.002	0.008		0.020	0.007	*	0.006	0.007		-0.027	0.012	*	-0.001	0.003	
Prepared ready to eat foods	0.054	0.020	*	0.026	0.018		-0.027	0.012	*	-0.062	0.045		0.001	0.006	
Sugar and preserves	0.000	0.004		-0.006	0.004	*	-0.001	0.003		0.001	0.006		0.000	0.002	
Condiments and sauces	0.003	0.004		0.008	0.004	*	0.003	0.003		-0.004	0.006		0.001	0.001	
Low calorie, soft drinks and juices	0.003	0.007		-0.004	0.006		-0.007	0.005		-0.013	0.011		-0.002	0.003	
Alcoholic beverages	-0.007	0.006		-0.008	0.004	*	-0.003	0.003		0.000	0.006		-0.003	0.002	
Numeraire category	-0.002	-0.167		-0.005	-0.148		-0.005	-0.104		0.002	-0.268		-0.002	-0.053	
**Total expenditure**	-0.001	0.003		-0.002	0.002		-0.004	0.002	*	-0.004	0.003		-0.002	0.001	*
**Promotions**															
Take home confectionery	-0.002	0.001	*	-0.001	0.001		0.001	0.001		0.001	0.001		0.000	0.000	
Biscuits	-0.002	0.001		-0.002	0.001	*	0.001	0.001		0.001	0.001		0.000	0.000	
Take home savouries	-0.002	0.001	*	-0.001	0.001		-0.001	0.001	*	0.000	0.001		0.000	0.000	
Cakes, pastries, and sugar morning goods	-0.003	0.001	*	-0.001	0.001	*	0.000	0.001		0.002	0.001		0.000	0.000	
Total puddings and desserts	0.001	0.001		0.000	0.001		0.000	0.001		0.000	0.001		0.000	0.000	
Take home sugary drinks	-0.001	0.001	*	0.000	0.000		0.000	0.000		0.000	0.001		0.000	0.000	
Edible ices and ice creams	0.000	0.001		0.000	0.001		0.000	0.000		0.000	0.001		0.000	0.000	
Dairy products	0.001	0.001		0.000	0.000		0.001	0.000	*	0.000	0.001		0.000	0.000	
Meat and fish	-0.003	0.001	*	0.000	0.001		0.000	0.001		0.002	0.001	*	0.000	0.000	
Fats and eggs	-0.001	0.001		-0.002	0.001	*	0.000	0.000		0.000	0.001		0.000	0.000	
Fruit	0.010	0.001	*	0.002	0.001	*	0.001	0.001		-0.002	0.001	*	0.000	0.000	
Vegetables	0.004	0.001	*	0.006	0.001	*	-0.001	0.001	*	-0.001	0.001		0.000	0.000	
Grains	0.002	0.001		-0.001	0.001		0.003	0.001	*	-0.002	0.001	*	0.001	0.000	*
Prepared ready to eat foods	-0.006	0.002	*	-0.003	0.001	*	-0.001	0.001		0.012	0.002	*	0.000	0.001	
Sugar and preserves	0.002	0.002		0.001	0.002		-0.002	0.001		0.002	0.002		0.004	0.001	*
Condiments and sauces	0.001	0.002		-0.001	0.001		0.001	0.001		0.001	0.002		0.001	0.001	
Low calorie, soft drinks and juices	0.000	0.000		-0.001	0.000	*	-0.001	0.000	*	0.000	0.000		0.000	0.000	*
Alcoholic beverages	-0.003	0.001	*	-0.001	0.001		-0.002	0.001	*	-0.003	0.001	*	-0.001	0.000	*
Numeraire category	-0.001	0.002		-0.002	0.001	*	0.002	0.001	*	-0.004	0.002	*	0.001	0.000	*

Variables	Equations											
	Condiments and sauces			Low calorie soft drinks and juices			Alcoholic beverages			Numeraire category		
	Coef.	St. Err.	Sig.	Coef.	St. Err.	Sig.	Coef.	St. Err.	Sig.	Coef.	St. Err.	Sig.
**Intercept**	0.021	0.010	*	0.060	0.017	*	0.120	0.020	*	0.134	0.106	
**Prices**												
Take home confectionery	-0.003	0.002		0.007	0.004	*	0.005	0.003	*	0.003	0.023	
Biscuits	-0.001	0.002		0.008	0.004	*	-0.001	0.002		0.000	0.027	
Take home savouries	-0.006	0.002	*	0.002	0.004		0.000	0.002		0.002	0.032	
Cakes, pastries, and sugar morning goods	-0.002	0.002		0.001	0.004		0.001	0.002		0.000	0.027	
Total puddings and desserts	0.001	0.001		0.005	0.002	*	0.000	0.001		0.001	0.018	
Take home sugary drinks	0.004	0.002	*	-0.005	0.004		-0.001	0.002		-0.002	0.025	
Edible ices and ice creams	-0.002	0.002		0.002	0.003		0.001	0.001		-0.001	0.021	
Dairy products	0.000	0.004		0.010	0.007		-0.009	0.005	*	-0.016	0.047	
Meat and fish	0.001	0.006		-0.013	0.012		0.003	0.007		0.003	0.076	
Fats and eggs	-0.003	0.002	*	-0.005	0.003		-0.001	0.002		-0.002	0.022	
Fruit	0.003	0.004		0.003	0.007		-0.007	0.006		-0.002	0.045	
Vegetables	0.008	0.004	*	-0.004	0.006		-0.008	0.004	*	-0.005	0.040	
Grains	0.003	0.003		-0.007	0.005		-0.003	0.003		-0.005	0.028	
Prepared ready to eat foods	-0.004	0.006		-0.013	0.011		0.000	0.006		0.002	0.075	
Sugar and preserves	0.001	0.001		-0.002	0.003		-0.003	0.002		-0.002	0.014	
Condiments and sauces	0.001	0.002		0.000	0.002		0.001	0.002		-0.001	0.013	
Low calorie, soft drinks and juices	0.000	0.002		-0.005	0.006		0.004	0.004		0.010	0.024	
Alcoholic beverages	0.001	0.002		0.004	0.004		0.012	0.013		0.005	0.020	
Numeraire category	-0.001	-0.050		0.010	-0.090		0.005	-0.066		0.009	0.567	
**Total expenditure**	-0.001	0.001		0.000	0.002		0.013	0.008	*	0.030	0.012	*
**Promotions**												
Take home confectionery	0.000	0.000		-0.001	0.001		-0.003	0.003		-0.001	0.005	
Biscuits	0.000	0.000		-0.002	0.001	*	0.003	0.003		-0.001	0.005	
Take home savouries	0.000	0.000		0.000	0.001		0.004	0.003		-0.001	0.004	
Cakes, pastries, and sugar morning goods	0.000	0.000		0.000	0.001		-0.007	0.003	*	0.002	0.004	
Total puddings and desserts	0.000	0.000		-0.001	0.001		0.001	0.003		-0.003	0.004	
Take home sugary drinks	0.000	0.000		0.000	0.000		0.000	0.002		0.000	0.002	
Edible ices and ice creams	0.000	0.000		-0.001	0.001	*	-0.006	0.002	*	0.003	0.003	
Dairy products	0.000	0.000		0.000	0.000		-0.002	0.002		0.000	0.002	
Meat and fish	0.002	0.000	*	0.001	0.001	*	-0.003	0.003		-0.001	0.004	
Fats and eggs	0.000	0.000		0.000	0.001		-0.002	0.002		0.000	0.003	
Fruit	0.000	0.000		0.001	0.001	*	-0.008	0.003	*	-0.002	0.004	
Vegetables	-0.001	0.000	*	0.001	0.001		-0.003	0.003		-0.003	0.005	
Grains	0.001	0.000		0.001	0.001		-0.003	0.003		-0.001	0.004	
Prepared ready to eat foods	-0.001	0.001	*	-0.002	0.001		0.001	0.005		0.005	0.007	
Sugar and preserves	0.000	0.001		-0.001	0.001		-0.004	0.006		-0.006	0.008	
Condiments and sauces	0.004	0.001	*	-0.001	0.001		-0.008	0.005	*	-0.002	0.007	
Low calorie, soft drinks and juices	0.000	0.000	*	0.002	0.000	*	0.003	0.001	*	-0.001	0.002	
Alcoholic beverages	-0.001	0.000		-0.002	0.001	*	0.023	0.003	*	-0.001	0.004	
Numeraire category	0.000	0.000		-0.003	0.001	*	0.001	0.004		0.015	0.005	*

Note: '*' indicates statistically significant at 5 per cent.

Table S7 - Scotland - Pre-family group - All categories - Demand model results

Variables	Equations											
	Take home confectionery			Biscuits			Take home savouries			Cakes, pastries and sugar morning goods		
	Coef.	St. Err.	Sig.	Coef.	St. Err.	Sig.	Coef.	St. Err.	Sig.	Coef.	St. Err.	Sig.
**Intercept**	0.073	0.013	*	0.036	0.015	*	0.023	0.019		0.000	0.016	
**Prices**												
Take home confectionery	0.000	0.004		-0.002	0.003		-0.002	0.004		0.006	0.003	*
Biscuits	-0.002	0.003		0.018	0.007	*	0.011	0.006		-0.005	0.004	
Take home savouries	-0.002	0.004		0.011	0.006		-0.004	0.011		0.006	0.005	
Cakes, pastries, and sugar morning goods	0.006	0.003	*	-0.005	0.004		0.006	0.005		-0.012	0.006	*
Total puddings and desserts	0.003	0.002	*	0.002	0.003		-0.002	0.004		0.001	0.002	
Take home sugary drinks	-0.003	0.004		0.004	0.004		-0.006	0.005		0.003	0.004	
Edible ices and ice creams	-0.005	0.002	*	-0.008	0.004	*	0.010	0.005	*	-0.001	0.003	
Dairy products	0.023	0.006	*	-0.007	0.006		0.001	0.008		-0.021	0.007	*
Meat and fish	-0.023	0.009	*	-0.015	0.010		0.010	0.013		0.024	0.010	*
Fats and eggs	-0.002	0.003		0.008	0.004	*	-0.013	0.005	*	-0.001	0.004	
Fruit	0.020	0.006	*	0.006	0.006		-0.008	0.008		-0.021	0.007	*
Vegetables	0.002	0.005		0.003	0.006		0.004	0.008		0.000	0.006	
Grains	0.003	0.003		0.002	0.004		0.008	0.005	*	-0.004	0.004	
Prepared ready to eat foods	-0.013	0.009		-0.018	0.011	*	0.001	0.014		0.024	0.011	*
Sugar and preserves	-0.002	0.002		-0.002	0.002		-0.005	0.002	*	0.001	0.002	
Condiments and sauces	-0.001	0.002		-0.001	0.002		-0.002	0.002		-0.003	0.002	
Low calorie, soft drinks and juices	0.001	0.003		0.007	0.003	*	-0.008	0.004	*	0.000	0.003	
Alcoholic beverages	-0.006	0.002	*	-0.003	0.002	*	-0.003	0.002		0.003	0.002	
Numeraire category	0.000	-0.070		-0.001	-0.086		0.002	-0.111		-0.001	-0.085	
**Total expenditure**	-0.002	0.001	*	-0.003	0.001	*	-0.004	0.001	*	-0.001	0.001	
**Promotions**												
Take home confectionery	0.007	0.000	*	0.001	0.000	*	0.000	0.000		0.000	0.000	
Biscuits	0.001	0.000	*	0.004	0.000	*	0.000	0.000		0.000	0.000	
Take home savouries	0.000	0.000		0.000	0.000		0.003	0.000	*	0.000	0.000	
Cakes, pastries, and sugar morning goods	-0.001	0.000		0.000	0.000		0.000	0.000		0.003	0.000	*
Total puddings and desserts	0.000	0.000		0.000	0.000		0.000	0.000		0.000	0.000	
Take home sugary drinks	0.000	0.000	*	0.000	0.000		0.000	0.000	*	0.000	0.000	
Edible ices and ice creams	0.001	0.000	*	0.000	0.000		0.000	0.000		0.000	0.000	
Dairy products	0.000	0.000		0.000	0.000		0.000	0.000	*	0.000	0.000	
Meat and fish	-0.001	0.000		0.000	0.000		0.000	0.000		-0.001	0.000	*
Fats and eggs	-0.001	0.000		0.000	0.000		0.000	0.000		0.000	0.000	
Fruit	-0.001	0.000		0.000	0.000		0.000	0.000		-0.001	0.000	*
Vegetables	-0.001	0.000		0.000	0.000		-0.002	0.000	*	-0.001	0.000	*
Grains	-0.001	0.000	*	0.000	0.000		-0.001	0.000	*	0.000	0.000	*
Prepared ready to eat foods	-0.001	0.000		-0.001	0.000	*	-0.001	0.000	*	0.000	0.000	
Sugar and preserves	0.001	0.001		0.000	0.000		0.001	0.001	*	0.000	0.000	
Condiments and sauces	0.000	0.000		0.000	0.000		-0.001	0.000	*	0.001	0.000	*
Low calorie, soft drinks and juices	0.001	0.000	*	0.000	0.000	*	0.000	0.000		0.000	0.000	
Alcoholic beverages	-0.002	0.001	*	-0.001	0.000	*	-0.002	0.000	*	-0.001	0.000	*
Numeraire category	0.000	0.001		0.000	0.000		0.000	0.000		0.000	0.000	
Log-Likelihood	57620.100											
Obs.	1348.000											

Variables	Total puddings and desserts			Take home sugary drinks			Edible ices and ice cream			Dairy products			Meat and fish			Fats and eggs		
	Coef.	St. Err.	Sig.	Coef.	St. Err.	Sig.	Coef.	St. Err.	Sig.	Coef.	St. Err.	Sig.	Coef.	St. Err.	Sig.	Coef.	St. Err.	Sig.
**Intercept**	0.036	0.009	*	-0.024	0.018		0.009	0.011		0.088	0.027	*	0.138	0.043	*	0.006	0.013	
**Prices**																		
Take home confectionery	0.003	0.002	*	-0.003	0.004		-0.005	0.002	*	0.023	0.006	*	-0.023	0.009	*	-0.002	0.003	
Biscuits	0.002	0.003		0.004	0.004		-0.008	0.004	*	-0.007	0.006		-0.015	0.010		0.008	0.004	*
Take home savouries	-0.002	0.004		-0.006	0.005		0.010	0.005	*	0.001	0.008		0.010	0.013		-0.013	0.005	*
Cakes, pastries, and sugar morning goods	0.001	0.002		0.003	0.004		-0.001	0.003		-0.021	0.007	*	0.024	0.010	*	-0.001	0.004	
Total puddings and desserts	-0.004	0.003	*	0.004	0.002	*	-0.004	0.002		0.005	0.004		-0.005	0.005		0.003	0.002	
Take home sugary drinks	0.004	0.002	*	-0.014	0.007	*	0.003	0.003		-0.001	0.007		0.005	0.011		-0.003	0.004	
Edible ices and ice creams	-0.004	0.002		0.003	0.003		-0.003	0.005		-0.003	0.005		0.002	0.007		0.006	0.003	*
Dairy products	0.005	0.004		-0.001	0.007		-0.003	0.005		0.046	0.016	*	0.007	0.018		0.000	0.006	
Meat and fish	-0.005	0.005		0.005	0.011		0.002	0.007		0.007	0.018		-0.050	0.037		0.007	0.008	
Fats and eggs	0.003	0.002		-0.003	0.004		0.006	0.003	*	0.000	0.006		0.007	0.008		0.001	0.005	
Fruit	0.000	0.003		0.002	0.007		0.001	0.005		-0.005	0.011		-0.015	0.018		-0.008	0.006	
Vegetables	0.004	0.003		0.000	0.007		0.000	0.004		-0.017	0.010		0.012	0.016		-0.004	0.005	
Grains	-0.002	0.002		-0.001	0.004		-0.002	0.003		-0.017	0.007	*	0.017	0.010	*	-0.001	0.003	
Prepared ready to eat foods	-0.009	0.006		0.001	0.012		0.009	0.008		-0.018	0.018		0.056	0.028	*	0.005	0.009	
Sugar and preserves	-0.001	0.001		0.004	0.002	*	0.001	0.001		0.001	0.003		0.001	0.005		0.001	0.002	
Condiments and sauces	0.000	0.001		0.004	0.002	*	-0.001	0.001		0.006	0.003	*	0.005	0.005		-0.001	0.001	
Low calorie, soft drinks and juices	0.006	0.002	*	-0.003	0.004		-0.005	0.002	*	0.010	0.006	*	-0.031	0.009	*	0.003	0.003	
Alcoholic beverages	0.000	0.001		-0.001	0.002		-0.001	0.001		-0.010	0.004	*	0.005	0.006		0.000	0.001	
Numeraire category	-0.001	-0.048		0.003	-0.091		-0.001	-0.066		0.001	-0.144		-0.012	-0.225		-0.002	-0.074	
**Total expenditure**	0.001	0.000	*	0.003	0.001	*	-0.002	0.001	*	-0.007	0.002	*	-0.007	0.003	*	-0.002	0.001	*
**Promotions**																		
Take home confectionery	0.000	0.000		0.002	0.000	*	0.000	0.000	*	0.000	0.001		-0.002	0.001	*	0.000	0.000	
Biscuits	0.000	0.000		-0.001	0.000	*	0.000	0.000	*	-0.001	0.001	*	0.001	0.001		-0.001	0.000	*
Take home savouries	0.000	0.000		0.000	0.000		0.000	0.000		-0.001	0.001	*	0.001	0.001		0.000	0.000	
Cakes, pastries, and sugar morning goods	0.000	0.000		0.000	0.000		0.000	0.000		0.000	0.001		-0.001	0.001		0.000	0.000	
Total puddings and desserts	0.003	0.000	*	0.001	0.000	*	0.000	0.000	*	0.000	0.001		-0.003	0.001	*	0.001	0.000	*
Take home sugary drinks	0.000	0.000		0.003	0.000	*	0.000	0.000		-0.001	0.000		0.000	0.001		0.000	0.000	
Edible ices and ice creams	0.000	0.000		0.000	0.000		0.002	0.000	*	-0.001	0.001		-0.001	0.001		0.000	0.000	
Dairy products	0.000	0.000	*	0.000	0.000		0.000	0.000		0.004	0.000	*	-0.001	0.001	*	0.000	0.000	
Meat and fish	0.000	0.000		0.000	0.000		-0.001	0.000	*	-0.001	0.001	*	0.007	0.001	*	0.000	0.000	
Fats and eggs	0.000	0.000		0.000	0.000		0.000	0.000		0.000	0.001		0.004	0.001	*	0.002	0.000	*
Fruit	0.000	0.000	*	-0.001	0.000	*	0.000	0.000		0.000	0.001		0.000	0.001		-0.001	0.000	*
Vegetables	0.000	0.000		0.000	0.000		-0.001	0.000	*	-0.001	0.001		0.001	0.001		0.001	0.000	*
Grains	0.000	0.000	*	0.000	0.000		0.000	0.000	*	0.001	0.000	*	-0.001	0.001	*	0.000	0.000	
Prepared ready to eat foods	0.000	0.000		0.000	0.000		0.000	0.000	*	0.001	0.001		-0.003	0.001	*	-0.001	0.000	*
Sugar and preserves	0.000	0.000		0.001	0.001		0.000	0.000		-0.002	0.001	*	0.000	0.001		-0.001	0.000	*
Condiments and sauces	0.000	0.000		0.000	0.000		0.000	0.000		-0.001	0.000		0.000	0.001		0.000	0.000	*
Low calorie, soft drinks and juices	0.000	0.000		0.000	0.000	*	0.000	0.000		-0.001	0.000	*	0.000	0.000		0.000	0.000	*
Alcoholic beverages	0.000	0.000	*	-0.002	0.000	*	0.000	0.000		-0.002	0.001	*	-0.002	0.001	*	-0.001	0.000	*
Numeraire category	0.000	0.000		-0.001	0.000		0.000	0.000		0.001	0.001		-0.002	0.001	*	0.000	0.000	

Variables	Fruit			Vegetables			Grains			Prepared ready to eat foods			Sugar and preserves			Condiments and sauces		
	Coef.	St. Err.	Sig.	Coef.	St. Err.	Sig.	Coef.	St. Err.	Sig.	Coef.	St. Err.	Sig.	Coef.	St. Err.	Sig.	Coef.	St. Err.	Sig.
**Intercept**	0.074	0.026	*	0.053	0.024	*	0.024	0.015		0.081	0.041	*	0.021	0.007	*	0.030	0.008	*
**Prices**																		
Take home confectionery	0.020	0.006	*	0.002	0.005		0.003	0.003		-0.013	0.009		-0.002	0.002		-0.001	0.002	
Biscuits	0.006	0.006		0.003	0.006		0.002	0.004		-0.018	0.011	*	-0.002	0.002		-0.001	0.002	
Take home savouries	-0.008	0.008		0.004	0.008		0.008	0.005	*	0.001	0.014		-0.005	0.002	*	-0.002	0.002	
Cakes, pastries, and sugar morning goods	-0.021	0.007	*	0.000	0.006		-0.004	0.004		0.024	0.011	*	0.001	0.002		-0.003	0.002	
Total puddings and desserts	0.000	0.003		0.004	0.003		-0.002	0.002		-0.009	0.006		-0.001	0.001		0.000	0.001	
Take home sugary drinks	0.002	0.007		0.000	0.007		-0.001	0.004		0.001	0.012		0.004	0.002	*	0.004	0.002	*
Edible ices and ice creams	0.001	0.005		0.000	0.004		-0.002	0.003		0.009	0.008		0.001	0.001		-0.001	0.001	
Dairy products	-0.005	0.011		-0.017	0.010		-0.017	0.007	*	-0.018	0.018		0.001	0.003		0.006	0.003	*
Meat and fish	-0.015	0.018		0.012	0.016		0.017	0.010	*	0.056	0.028	*	0.001	0.005		0.005	0.005	
Fats and eggs	-0.008	0.006		-0.004	0.005		-0.001	0.003		0.005	0.009		0.001	0.002		-0.001	0.001	
Fruit	0.005	0.017		-0.014	0.011		0.013	0.007	*	0.022	0.019		0.002	0.003		-0.003	0.003	
Vegetables	-0.014	0.011		0.001	0.014		0.000	0.006		0.007	0.017		-0.006	0.003	*	0.000	0.003	
Grains	0.013	0.007	*	0.000	0.006		0.000	0.006		-0.007	0.011		-0.002	0.002		0.004	0.002	*
Prepared ready to eat foods	0.022	0.019		0.007	0.017		-0.007	0.011		-0.075	0.040	*	0.006	0.005		0.001	0.005	
Sugar and preserves	0.002	0.003		-0.006	0.003	*	-0.002	0.002		0.006	0.005		-0.001	0.001		0.001	0.001	
Condiments and sauces	-0.003	0.003		0.000	0.003		0.004	0.002	*	0.001	0.005		0.001	0.001		-0.003	0.001	*
Low calorie, soft drinks and juices	0.010	0.006		0.015	0.005	*	-0.010	0.004	*	0.011	0.009		0.001	0.002		-0.003	0.002	
Alcoholic beverages	-0.007	0.004		0.001	0.004		-0.003	0.002		0.007	0.005		-0.001	0.001		-0.001	0.001	
Numeraire category	-0.001	-0.147		-0.009	-0.133		-0.002	-0.085		-0.011	-0.235		0.000	-0.039		-0.002	-0.040	
**Total expenditure**	-0.009	0.002	*	-0.004	0.002	*	-0.010	0.001	*	-0.005	0.003	*	-0.001	0.000		-0.003	0.001	*
**Promotions**																		
Take home confectionery	-0.001	0.001		-0.001	0.001		0.000	0.000		-0.004	0.001	*	0.000	0.000		0.000	0.000	
Biscuits	0.001	0.001		-0.001	0.001		0.000	0.000		0.000	0.001		0.000	0.000		0.000	0.000	
Take home savouries	0.001	0.001		0.002	0.001	*	0.000	0.000		-0.002	0.001	*	0.000	0.000		0.000	0.000	*
Cakes, pastries, and sugar morning goods	-0.001	0.001		0.000	0.001		-0.001	0.000		-0.002	0.001	*	0.000	0.000	*	0.000	0.000	*
Total puddings and desserts	-0.003	0.001	*	-0.003	0.001	*	0.000	0.000		0.002	0.001		0.000	0.000	*	0.001	0.000	*
Take home sugary drinks	-0.001	0.000	*	-0.001	0.000	*	0.000	0.000		0.001	0.000	*	0.000	0.000		0.000	0.000	
Edible ices and ice creams	0.000	0.001		-0.001	0.001		-0.001	0.000	*	-0.001	0.001		0.000	0.000		0.000	0.000	
Dairy products	0.000	0.000		0.000	0.000		0.001	0.000	*	-0.001	0.001	*	0.000	0.000		0.000	0.000	*
Meat and fish	-0.002	0.001	*	-0.002	0.001	*	0.000	0.000		-0.001	0.001		0.000	0.000		0.000	0.000	
Fats and eggs	-0.002	0.001	*	-0.001	0.001		-0.001	0.000	*	-0.002	0.001	*	0.000	0.000		0.000	0.000	*
Fruit	0.010	0.001	*	0.001	0.001		0.000	0.000		-0.001	0.001		-0.001	0.000	*	0.000	0.000	
Vegetables	0.003	0.001	*	0.009	0.001	*	-0.001	0.001		-0.003	0.001	*	0.000	0.000		0.000	0.000	
Grains	-0.001	0.001		0.001	0.000		0.004	0.000	*	0.001	0.001		0.001	0.000	*	0.000	0.000	*
Prepared ready to eat foods	-0.003	0.001	*	-0.003	0.001	*	-0.001	0.000	*	0.016	0.001	*	0.000	0.000	*	0.000	0.000	
Sugar and preserves	-0.002	0.001		-0.002	0.001	*	0.000	0.001		0.006	0.001	*	0.002	0.000	*	-0.001	0.000	*
Condiments and sauces	0.000	0.001		0.001	0.001		0.001	0.000	*	0.000	0.001		0.000	0.000		0.002	0.000	*
Low calorie, soft drinks and juices	-0.001	0.000	*	-0.001	0.000	*	0.000	0.000	*	0.000	0.000		0.000	0.000		0.000	0.000	
Alcoholic beverages	-0.001	0.001		-0.002	0.001	*	-0.001	0.001	*	-0.002	0.001		-0.001	0.000	*	0.000	0.000	
Numeraire category	0.000	0.001		-0.002	0.001	*	0.000	0.001		-0.003	0.001	*	0.000	0.000		0.000	0.000	

Variables	Low calorie soft drinks and juices			Alcoholic beverages			Numeraire category		
	Coef.	St. Err.	Sig.	Coef.	St. Err.	Sig.	Coef.	St. Err.	Sig.
**Intercept**	0.082	0.013	*	0.071	0.012	*	0.108	0.090	
**Prices**									
Take home confectionery	0.001	0.003		-0.006	0.002	*	0.002	0.019	
Biscuits	0.007	0.003	*	-0.003	0.002	*	-0.001	0.023	
Take home savouries	-0.008	0.004	*	-0.003	0.002		-0.001	0.030	
Cakes, pastries, and sugar morning goods	0.000	0.003		0.003	0.002		-0.001	0.023	
Total puddings and desserts	0.006	0.002	*	0.000	0.001		0.000	0.013	
Take home sugary drinks	-0.003	0.004		-0.001	0.002		-0.001	0.025	
Edible ices and ice creams	-0.005	0.002	*	-0.001	0.001		0.000	0.018	
Dairy products	0.010	0.006	*	-0.010	0.004	*	-0.003	0.039	
Meat and fish	-0.031	0.009	*	0.005	0.006		-0.005	0.063	
Fats and eggs	0.003	0.003		0.000	0.001		-0.001	0.020	
Fruit	0.010	0.006		-0.007	0.004		0.002	0.040	
Vegetables	0.015	0.005	*	0.001	0.004		-0.001	0.036	
Grains	-0.010	0.004	*	-0.003	0.002		-0.002	0.023	
Prepared ready to eat foods	0.011	0.009		0.007	0.005		-0.007	0.066	
Sugar and preserves	0.001	0.002		-0.001	0.001		-0.001	0.010	
Condiments and sauces	-0.003	0.002		-0.001	0.001		0.000	0.011	
Low calorie, soft drinks and juices	-0.004	0.005		-0.004	0.003		-0.001	0.019	
Alcoholic beverages	-0.004	0.003		0.015	0.007	*	0.003	0.014	
Numeraire category	0.003	-0.072		0.008	-0.050		0.018	0.486	
**Total expenditure**	-0.005	0.001	*	0.007	0.003	*	0.019	0.007	*
**Promotions**									
Take home confectionery	0.000	0.001		-0.003	0.001	*	0.001	0.003	
Biscuits	0.000	0.001		-0.003	0.001	*	0.002	0.003	
Take home savouries	0.000	0.000		0.000	0.001		-0.001	0.002	
Cakes, pastries, and sugar morning goods	0.001	0.000		0.001	0.001		-0.001	0.002	
Total puddings and desserts	0.001	0.001	*	-0.003	0.001	*	0.000	0.003	
Take home sugary drinks	-0.001	0.000	*	-0.001	0.001		0.000	0.001	
Edible ices and ice creams	0.000	0.000		0.001	0.001		-0.001	0.002	
Dairy products	0.000	0.000		-0.002	0.001	*	0.001	0.001	
Meat and fish	0.000	0.001		0.003	0.001	*	-0.001	0.003	
Fats and eggs	-0.001	0.000		0.003	0.001	*	-0.001	0.002	
Fruit	-0.001	0.000	*	-0.004	0.001	*	-0.003	0.002	
Vegetables	-0.001	0.001	*	-0.003	0.002	*	-0.002	0.003	
Grains	0.000	0.000		-0.002	0.001	*	-0.001	0.002	
Prepared ready to eat foods	-0.001	0.000	*	-0.002	0.001	*	0.000	0.003	
Sugar and preserves	0.001	0.001	*	-0.003	0.002		-0.002	0.004	
Condiments and sauces	-0.001	0.000	*	-0.001	0.001		-0.003	0.002	
Low calorie, soft drinks and juices	0.004	0.000	*	0.001	0.001		0.000	0.001	
Alcoholic beverages	-0.001	0.001	*	0.022	0.002	*	-0.003	0.003	
Numeraire category	0.000	0.001		-0.002	0.002		0.017	0.003	*

Note: '*' indicates statistically significant at 5 per cent.

Table S8 - Scotland - Young family group - All categories - Demand model results

Equations

Variables	Take home confectionery			Biscuits			Take home savouries			Cakes, pastries and sugar morning goods		
	Coef.	St. Err.	Sig.	Coef.	St. Err.	Sig.	Coef.	St. Err.	Sig.	Coef.	St. Err.	Sig.
**Intercept**	0.057	0.012	*	0.004	0.014		0.090	0.016	*	0.047	0.013	*
**Prices**												
Take home confectionery	0.003	0.004		-0.009	0.003	*	-0.002	0.003		-0.002	0.003	
Biscuits	-0.009	0.003	*	-0.003	0.006		-0.009	0.006	*	-0.005	0.004	
Take home savouries	-0.002	0.003		-0.009	0.006	*	-0.004	0.009		0.012	0.004	*
Cakes, pastries, and sugar morning go	-0.002	0.003		-0.005	0.004		0.012	0.004	*	0.000	0.005	
Total puddings and desserts	-0.001	0.001		0.001	0.003		0.002	0.003		0.001	0.002	
Take home sugary drinks	0.007	0.003	*	0.004	0.004		0.006	0.004		0.004	0.003	
Edible ices and ice creams	0.001	0.002		0.006	0.003	*	-0.012	0.004	*	-0.002	0.003	
Dairy products	-0.004	0.005		-0.003	0.006		0.006	0.007		0.003	0.005	
Meat and fish	-0.013	0.008	*	0.028	0.009	*	-0.040	0.009	*	-0.003	0.008	
Fats and eggs	0.000	0.002		0.003	0.004		0.004	0.004		0.003	0.003	
Fruit	0.006	0.005		-0.002	0.006		-0.016	0.006	*	0.000	0.005	
Vegetables	0.008	0.005	*	0.001	0.005		0.002	0.006		0.010	0.005	*
Grains	0.002	0.003		0.012	0.004	*	-0.001	0.004		0.003	0.003	
Prepared ready to eat foods	0.006	0.007		-0.016	0.010		0.056	0.011	*	-0.014	0.009	
Sugar and preserves	0.001	0.001		0.001	0.002		0.001	0.002		-0.003	0.002	*
Condiments and sauces	-0.001	0.001		-0.001	0.002		-0.005	0.002	*	0.000	0.002	
Low calorie, soft drinks and juices	-0.004	0.003		-0.001	0.003		0.003	0.003		-0.006	0.003	*
Alcoholic beverages	0.004	0.003		0.000	0.002		0.000	0.002		0.000	0.002	
Numeraire category	-0.002	-0.063		-0.005	-0.079		-0.001	-0.089		-0.002	-0.070	
**Total expenditure**	0.007	0.002	*	-0.003	0.001	*	-0.004	0.001	*	-0.002	0.001	*
**Promotions**												
Take home confectionery	0.009	0.001	*	0.001	0.000	*	0.001	0.000	*	0.001	0.000	*
Biscuits	0.000	0.000		0.004	0.000	*	0.001	0.000	*	0.000	0.000	
Take home savouries	0.000	0.000		0.000	0.000		0.003	0.000	*	0.000	0.000	
Cakes, pastries, and sugar morning go	0.000	0.000		0.000	0.000		0.000	0.000	*	0.002	0.000	*
Total puddings and desserts	0.000	0.000		0.000	0.000		0.000	0.000		0.000	0.000	
Take home sugary drinks	0.000	0.000	*	0.000	0.000	*	0.000	0.000		0.000	0.000	
Edible ices and ice creams	0.002	0.000	*	0.000	0.000		0.000	0.000		0.001	0.000	*
Dairy products	0.000	0.000		0.000	0.000		0.000	0.000		0.000	0.000	
Meat and fish	-0.001	0.001	*	-0.001	0.000	*	-0.001	0.000	*	0.000	0.000	
Fats and eggs	0.000	0.000		0.000	0.000		-0.001	0.000	*	0.000	0.000	
Fruit	0.000	0.000		0.000	0.000		0.000	0.000		0.000	0.000	
Vegetables	-0.001	0.001		0.000	0.000		-0.001	0.000	*	0.000	0.000	
Grains	0.000	0.000		0.000	0.000		0.001	0.000	*	0.000	0.000	
Prepared ready to eat foods	-0.001	0.001		0.000	0.000		0.001	0.000		0.000	0.000	
Sugar and preserves	0.002	0.001	*	0.001	0.000		-0.001	0.001	*	0.000	0.000	
Condiments and sauces	0.000	0.001		0.000	0.000		0.000	0.000		-0.001	0.000	*
Low calorie, soft drinks and juices	-0.001	0.000	*	0.000	0.000	*	0.000	0.000		0.000	0.000	*
Alcoholic beverages	0.000	0.001		-0.001	0.000	*	0.000	0.000		-0.001	0.000	*
Numeraire category	-0.004	0.001	*	-0.001	0.001	*	-0.002	0.001	*	-0.001	0.001	
Log-Likelihood	53950.300											
Obs.	1168.000											

Variables	Total puddings and desserts			Take home sugary drinks			Edible ices and ice cream			Dairy products			Meat and fish		
	Coef.	St. Err.	Sig.	Coef.	St. Err.	Sig.	Coef.	St. Err.	Sig.	Coef.	St. Err.	Sig.	Coef.	St. Err.	Sig.
**Intercept**	0.014	0.008	*	-0.003	0.015		0.015	0.009		0.112	0.023	*	0.061	0.037	*
**Prices**															
Take home confectionery	-0.001	0.001		0.007	0.003	*	0.001	0.002		-0.004	0.005		-0.013	0.008	*
Biscuits	0.001	0.003		0.004	0.004		0.006	0.003	*	-0.003	0.006		0.028	0.009	*
Take home savouries	0.002	0.003		0.006	0.004		-0.012	0.004	*	0.006	0.007		-0.040	0.009	*
Cakes, pastries, and sugar morning go	0.001	0.002		0.004	0.003		-0.002	0.003		0.003	0.005		-0.003	0.008	
Total puddings and desserts	-0.003	0.002		0.002	0.002		-0.002	0.002		-0.004	0.003		0.002	0.005	
Take home sugary drinks	0.002	0.002		-0.014	0.005	*	0.002	0.002		0.004	0.006		-0.007	0.009	
Edible ices and ice creams	-0.002	0.002		0.002	0.002		0.008	0.004	*	0.000	0.004		-0.013	0.006	*
Dairy products	-0.004	0.003		0.004	0.006		0.000	0.004		0.003	0.013		-0.003	0.015	
Meat and fish	0.002	0.005		-0.007	0.009		-0.013	0.006	*	-0.003	0.015		0.073	0.030	*
Fats and eggs	0.002	0.002		-0.006	0.003	*	-0.002	0.003		0.002	0.005		-0.006	0.007	
Fruit	0.001	0.003		-0.016	0.006	*	0.007	0.004	*	0.005	0.009		0.018	0.014	
Vegetables	-0.002	0.003		-0.009	0.005	*	-0.002	0.004		-0.016	0.009	*	-0.010	0.013	
Grains	0.002	0.002		0.006	0.004		0.000	0.002		0.005	0.006		-0.015	0.009	*
Prepared ready to eat foods	0.000	0.006		0.009	0.010		0.006	0.007		-0.007	0.015		-0.002	0.022	
Sugar and preserves	0.000	0.001		0.000	0.002		-0.001	0.001		0.003	0.003		-0.006	0.004	
Condiments and sauces	0.000	0.001		0.000	0.002		0.001	0.001		0.003	0.003		0.001	0.004	
Low calorie, soft drinks and juices	-0.001	0.001		0.005	0.003		0.004	0.002	*	-0.001	0.005		0.003	0.008	
Alcoholic beverages	0.000	0.001		0.003	0.002		0.000	0.001		-0.003	0.004		0.005	0.006	
Numeraire category	0.000	-0.043		0.001	-0.076		-0.002	-0.053		0.009	-0.123		-0.012	-0.186	
**Total expenditure**	0.000	0.001		0.002	0.001	*	-0.001	0.001		0.002	0.002		-0.009	0.004	*
**Promotions**															
Take home confectionery	0.000	0.000	*	0.001	0.000	*	0.000	0.000		-0.001	0.001		-0.002	0.001	
Biscuits	0.000	0.000		-0.001	0.000	*	0.000	0.000		-0.001	0.001		-0.001	0.001	
Take home savouries	0.000	0.000	*	0.000	0.000		0.000	0.000		-0.001	0.000	*	0.001	0.001	
Cakes, pastries, and sugar morning go	0.000	0.000		0.000	0.000		0.000	0.000		0.000	0.001		-0.001	0.001	
Total puddings and desserts	0.002	0.000	*	0.000	0.000		0.000	0.000		0.001	0.000		0.000	0.001	
Take home sugary drinks	0.000	0.000		0.002	0.000	*	0.000	0.000		-0.001	0.000	*	-0.001	0.000	*
Edible ices and ice creams	0.000	0.000		0.001	0.000	*	0.003	0.000	*	-0.002	0.001	*	-0.002	0.001	
Dairy products	0.000	0.000		0.000	0.000	*	0.000	0.000		0.003	0.000	*	-0.002	0.001	*
Meat and fish	0.000	0.000		-0.001	0.000	*	0.000	0.000	*	0.000	0.001		0.008	0.001	*
Fats and eggs	0.000	0.000		0.000	0.000		0.000	0.000		-0.001	0.000		0.001	0.001	
Fruit	0.000	0.000		0.000	0.000		0.000	0.000		0.001	0.001	*	-0.001	0.001	
Vegetables	0.000	0.000		-0.001	0.000	*	0.000	0.000		0.001	0.001	*	0.000	0.001	
Grains	0.000	0.000	*	0.000	0.000		0.000	0.000		0.001	0.001		0.000	0.001	
Prepared ready to eat foods	0.000	0.000		0.002	0.001	*	0.000	0.000		-0.003	0.001	*	-0.003	0.001	*
Sugar and preserves	0.000	0.000		0.000	0.001		0.000	0.000		-0.001	0.001		-0.003	0.002	*
Condiments and sauces	0.000	0.000		0.000	0.000		0.000	0.000		0.000	0.001		-0.003	0.001	*
Low calorie, soft drinks and juices	0.000	0.000	*	-0.001	0.000	*	0.000	0.000		0.000	0.000		0.000	0.000	
Alcoholic beverages	0.000	0.000	*	-0.001	0.000	*	0.000	0.000		-0.004	0.001	*	-0.001	0.001	
Numeraire category	0.000	0.000		-0.002	0.001	*	-0.002	0.000	*	-0.002	0.001	*	-0.005	0.002	*

Variables	Fats and eggs			Fruit			Vegetables			Grains			Prepared ready to eat foods		
	Coef.	St. Err.	Sig.	Coef.	St. Err.	Sig.	Coef.	St. Err.	Sig.	Coef.	St. Err.	Sig.	Coef.	St. Err.	Sig.
**Intercept**	0.026	0.012	*	0.066	0.022	*	0.065	0.021	*	0.068	0.014	*	0.074	0.035	*
**Prices**															
Take home confectionery	0.000	0.002		0.006	0.005		0.008	0.005	*	0.002	0.003		0.006	0.007	
Biscuits	0.003	0.004		-0.002	0.006		0.001	0.005		0.012	0.004	*	-0.016	0.010	
Take home savouries	0.004	0.004		-0.016	0.006	*	0.002	0.006		-0.001	0.004		0.056	0.011	*
Cakes, pastries, and sugar morning go	0.003	0.003		0.000	0.005		0.010	0.005	*	0.003	0.003		-0.014	0.009	
Total puddings and desserts	0.002	0.002		0.001	0.003		-0.002	0.003		0.002	0.002		0.000	0.006	
Take home sugary drinks	-0.006	0.003	*	-0.016	0.006	*	-0.009	0.005	*	0.006	0.004		0.009	0.010	
Edible ices and ice creams	-0.002	0.003		0.007	0.004	*	-0.002	0.004		0.000	0.002		0.006	0.007	
Dairy products	0.002	0.005		0.005	0.009		-0.016	0.009	*	0.005	0.006		-0.007	0.015	
Meat and fish	-0.006	0.007		0.018	0.014		-0.010	0.013		-0.015	0.009	*	-0.002	0.022	
Fats and eggs	0.003	0.004		-0.007	0.005		-0.001	0.005		-0.005	0.003		0.006	0.008	
Fruit	-0.007	0.005		-0.001	0.013		0.001	0.009		-0.003	0.006		-0.005	0.015	
Vegetables	-0.001	0.005		0.001	0.009		0.005	0.011		0.000	0.005		0.006	0.014	
Grains	-0.005	0.003		-0.003	0.006		0.000	0.005		0.003	0.005		0.008	0.009	
Prepared ready to eat foods	0.006	0.008		-0.005	0.015		0.006	0.014		0.008	0.009		-0.033	0.033	
Sugar and preserves	-0.002	0.001		0.000	0.003		0.005	0.002	*	0.000	0.002		0.002	0.004	
Condiments and sauces	0.001	0.001		0.003	0.003		0.007	0.002	*	0.000	0.002		-0.011	0.004	*
Low calorie, soft drinks and juices	0.007	0.003	*	0.012	0.005	*	0.004	0.005		-0.013	0.003	*	-0.009	0.008	
Alcoholic beverages	-0.001	0.002		-0.008	0.004	*	-0.011	0.003	*	-0.003	0.002		0.011	0.005	*
Numeraire category	0.000	-0.066		0.008	-0.120		0.004	-0.110		0.000	-0.075		-0.012	-0.197	
**Total expenditure**	-0.004	0.001	*	0.000	0.002		-0.006	0.002	*	-0.008	0.001	*	-0.017	0.003	*
**Promotions**															
Take home confectionery	0.000	0.000		-0.003	0.001	*	-0.002	0.001	*	-0.001	0.000	*	0.001	0.001	
Biscuits	-0.001	0.000	*	0.000	0.001		-0.001	0.001		-0.001	0.000	*	-0.001	0.001	
Take home savouries	0.000	0.000	*	-0.001	0.001	*	-0.001	0.000	*	0.000	0.000		0.000	0.001	
Cakes, pastries, and sugar morning go	0.000	0.000	*	0.001	0.001	*	0.000	0.000		-0.001	0.000	*	0.002	0.001	*
Total puddings and desserts	0.000	0.000		0.001	0.000	*	-0.001	0.000		0.000	0.000		0.000	0.001	
Take home sugary drinks	0.000	0.000	*	-0.001	0.000	*	-0.001	0.000	*	0.000	0.000		0.001	0.000	*
Edible ices and ice creams	0.000	0.000		-0.001	0.001		-0.001	0.001	*	-0.001	0.000	*	0.002	0.001	*
Dairy products	0.000	0.000		0.000	0.000		0.000	0.000		0.000	0.000	*	-0.001	0.001	
Meat and fish	0.000	0.000		-0.001	0.001		0.001	0.001		0.000	0.000		-0.002	0.001	*
Fats and eggs	0.002	0.000	*	0.001	0.000	*	0.001	0.000	*	0.000	0.000		-0.001	0.001	*
Fruit	0.000	0.000		0.006	0.001	*	0.000	0.000		0.000	0.000		0.000	0.001	
Vegetables	0.000	0.000		0.002	0.001	*	0.005	0.001	*	0.001	0.000		-0.003	0.001	*
Grains	0.000	0.000		-0.001	0.001	*	-0.001	0.001	*	0.002	0.000	*	0.001	0.001	
Prepared ready to eat foods	-0.001	0.000	*	-0.003	0.001	*	-0.004	0.001	*	0.000	0.001		0.012	0.001	*
Sugar and preserves	0.000	0.000		-0.001	0.001		-0.002	0.001	*	0.000	0.001		0.004	0.001	*
Condiments and sauces	0.000	0.000		-0.001	0.001		0.000	0.001		0.001	0.000	*	0.002	0.001	*
Low calorie, soft drinks and juices	0.000	0.000	*	0.000	0.000		0.000	0.000	*	0.000	0.000	*	0.000	0.000	
Alcoholic beverages	0.000	0.000		-0.004	0.001	*	-0.001	0.001	*	-0.002	0.000	*	-0.003	0.001	*
Numeraire category	0.001	0.001		-0.003	0.001	*	0.003	0.001	*	0.001	0.001		0.002	0.002	

Variables	Sugar and preserves			Condiments and sauces			Low calorie soft drinks and juices			Alcoholic beverages			Numeraire category		
	Coef.	St. Err.	Sig.	Coef.	St. Err.	Sig.	Coef.	St. Err.	Sig.	Coef.	St. Err.	Sig.	Coef.	St. Err.	Sig.
**Intercept**	0.019	0.006	*	0.031	0.007	*	0.037	0.012	*	0.068	0.013	*	0.149	0.077	*
**Prices**															
Take home confectionery	0.001	0.001		-0.001	0.001		-0.004	0.003		0.004	0.003		-0.002	0.017	
Biscuits	0.001	0.002		-0.001	0.002		-0.001	0.003		0.000	0.002		-0.005	0.021	
Take home savouries	0.001	0.002		-0.005	0.002	*	0.003	0.003		0.000	0.002		-0.001	0.024	
Cakes, pastries, and sugar morning go	-0.003	0.002	*	0.000	0.002		-0.006	0.003	*	0.000	0.002		-0.002	0.019	
Total puddings and desserts	0.000	0.001		0.000	0.001		-0.001	0.001		0.000	0.001		0.000	0.011	
Take home sugary drinks	0.000	0.002		0.000	0.002		0.005	0.003		0.003	0.002		0.001	0.020	
Edible ices and ice creams	-0.001	0.001		0.001	0.001		0.004	0.002	*	0.000	0.001		-0.002	0.014	
Dairy products	0.003	0.003		0.003	0.003		-0.001	0.005		-0.003	0.004		0.009	0.033	
Meat and fish	-0.006	0.004		0.001	0.004		0.003	0.008		0.005	0.006		-0.012	0.052	
Fats and eggs	-0.002	0.001		0.001	0.001		0.007	0.003	*	-0.001	0.002		0.000	0.018	
Fruit	0.000	0.003		0.003	0.003		0.012	0.005	*	-0.008	0.004	*	0.008	0.032	
Vegetables	0.005	0.002	*	0.007	0.002	*	0.004	0.005		-0.011	0.003	*	0.004	0.029	
Grains	0.000	0.002		0.000	0.002		-0.013	0.003	*	-0.003	0.002		0.000	0.020	
Prepared ready to eat foods	0.002	0.004		-0.011	0.004	*	-0.009	0.008		0.011	0.005	*	-0.012	0.055	
Sugar and preserves	-0.002	0.001	*	0.000	0.001		0.000	0.001		0.001	0.001		-0.002	0.009	
Condiments and sauces	0.000	0.001		0.000	0.001		-0.001	0.001		0.002	0.001	*	0.002	0.009	
Low calorie, soft drinks and juices	0.000	0.001		-0.001	0.001		-0.002	0.004		0.001	0.003		0.000	0.017	
Alcoholic beverages	0.001	0.001		0.002	0.001	*	0.001	0.003		-0.011	0.007		0.009	0.014	
Numeraire category	-0.002	-0.033		0.002	-0.033		0.000	-0.064		0.009	-0.050		0.005	0.409	
**Total expenditure**	-0.002	0.001	*	-0.004	0.001	*	0.001	0.002		0.007	0.004	*	0.040	0.008	*
**Promotions**															
Take home confectionery	0.000	0.000		-0.001	0.000	*	-0.001	0.000		-0.003	0.001	*	0.000	0.003	
Biscuits	0.000	0.000		0.000	0.000	*	0.000	0.000		-0.001	0.001		0.002	0.002	
Take home savouries	0.000	0.000		0.000	0.000		0.000	0.000		-0.001	0.001		0.000	0.002	
Cakes, pastries, and sugar morning go	0.000	0.000		0.000	0.000		0.000	0.000		-0.001	0.001		-0.002	0.002	
Total puddings and desserts	0.000	0.000		0.000	0.000		0.000	0.000		0.000	0.001		-0.002	0.002	
Take home sugary drinks	0.000	0.000		0.000	0.000	*	0.000	0.000		0.000	0.000		0.001	0.001	
Edible ices and ice creams	0.000	0.000		0.000	0.000		0.000	0.000		0.000	0.001		-0.001	0.002	
Dairy products	0.000	0.000	*	0.000	0.000	*	0.000	0.000		-0.001	0.001	*	0.000	0.001	
Meat and fish	0.000	0.000		0.000	0.000		0.000	0.000		0.001	0.001		-0.003	0.002	
Fats and eggs	0.000	0.000		0.000	0.000		0.000	0.000		-0.001	0.001		-0.002	0.002	
Fruit	0.000	0.000		0.000	0.000		0.000	0.000		-0.003	0.001	*	-0.001	0.002	
Vegetables	0.000	0.000		0.000	0.000		0.000	0.001		-0.001	0.001		-0.002	0.003	
Grains	0.000	0.000		0.000	0.000	*	0.000	0.000		0.000	0.001		-0.002	0.002	
Prepared ready to eat foods	-0.001	0.000	*	0.000	0.000		0.000	0.001		0.002	0.002		0.001	0.003	
Sugar and preserves	0.003	0.000	*	0.000	0.000		0.001	0.001		-0.001	0.002		-0.001	0.004	
Condiments and sauces	0.000	0.000		0.003	0.000	*	0.000	0.001		-0.002	0.002		0.001	0.003	
Low calorie, soft drinks and juices	0.000	0.000	*	0.000	0.000		0.002	0.000	*	0.001	0.000	*	-0.001	0.001	
Alcoholic beverages	-0.001	0.000	*	-0.001	0.000	*	0.000	0.000		0.019	0.001	*	0.000	0.003	
Numeraire category	0.000	0.000		0.001	0.000		-0.002	0.001	*	0.001	0.002		0.017	0.005	*

Note: '*' indicates statistically significant at 5 per cent.

Table S9 - Scotland - Middle family group - All categories - Demand model results

Equations

Variables	Take home confectionery			Biscuits			Take home savouries			Cakes, pastries and sugar morning goods			Total puddings and desserts		
	Coef.	St. Err.	Sig.	Coef.	St. Err.	Sig.	Coef.	St. Err.	Sig.	Coef.	St. Err.	Sig.	Coef.	St. Err.	Sig.
**Intercept**	0.081	0.014	*	0.046	0.017	*	0.036	0.018	*	0.020	0.017		0.011	0.010	
**Prices**															
Take home confectionery	-0.012	0.005	*	0.005	0.003		-0.010	0.003	*	-0.006	0.003		-0.002	0.002	
Biscuits	0.005	0.003		-0.004	0.008		0.006	0.006		-0.002	0.005		-0.005	0.003	
Take home savouries	-0.010	0.003	*	0.006	0.006		-0.007	0.010		-0.006	0.005		0.000	0.004	
Cakes, pastries, and sugar morning goods	-0.006	0.003		-0.002	0.005		-0.006	0.005		-0.002	0.006		-0.001	0.003	
Total puddings and desserts	-0.002	0.002		-0.005	0.003		0.000	0.004		-0.001	0.003		-0.004	0.003	
Take home sugary drinks	0.007	0.004		0.009	0.005	*	0.013	0.005	*	-0.001	0.005		0.000	0.003	
Edible ices and ice creams	0.000	0.002		-0.003	0.004		0.007	0.005		0.009	0.003	*	0.001	0.003	
Dairy products	-0.003	0.005		0.009	0.007		0.016	0.007	*	-0.008	0.007		0.006	0.004	
Meat and fish	-0.004	0.010		-0.003	0.011		0.000	0.012		0.005	0.011		-0.002	0.006	
Fats and eggs	-0.002	0.003		-0.002	0.004		0.000	0.005		0.003	0.004		0.000	0.003	
Fruit	0.005	0.006		-0.015	0.007	*	-0.011	0.007		-0.008	0.007		-0.003	0.004	
Vegetables	0.001	0.005		-0.004	0.006		-0.002	0.006		-0.003	0.006		-0.003	0.004	
Grains	0.005	0.004		0.008	0.005	*	-0.001	0.005		-0.001	0.005		0.004	0.003	
Prepared ready to eat foods	0.006	0.009		-0.001	0.012		-0.004	0.013		0.013	0.012		0.009	0.007	
Sugar and preserves	-0.004	0.002	*	0.001	0.002		0.000	0.002		-0.002	0.002		0.001	0.001	
Condiments and sauces	-0.001	0.002		-0.001	0.002		0.002	0.002		0.001	0.002		0.001	0.001	
Low calorie, soft drinks and juices	0.002	0.004		0.008	0.003	*	-0.002	0.003		0.007	0.003	*	-0.001	0.002	
Alcoholic beverages	0.000	0.003		-0.001	0.002		-0.002	0.002		0.001	0.002		-0.001	0.001	
Numeraire category	0.012	-0.076		-0.006	-0.096		0.002	-0.103		-0.001	-0.090		0.000	-0.056	
**Total expenditure**	0.016	0.002	*	-0.002	0.001		-0.003	0.001	*	-0.004	0.001	*	-0.001	0.001	*
**Promotions**															
Take home confectionery	0.008	0.001	*	0.001	0.000		0.000	0.000		0.000	0.000		0.000	0.000	
Biscuits	0.001	0.001		0.003	0.000	*	0.000	0.000		0.000	0.001		0.000	0.000	
Take home savouries	-0.001	0.001	*	0.000	0.000		0.002	0.000	*	0.000	0.000		0.000	0.000	*
Cakes, pastries, and sugar morning goods	0.000	0.000		0.000	0.000		0.000	0.000		0.002	0.000	*	0.000	0.000	
Total puddings and desserts	0.000	0.001		0.001	0.000	*	0.000	0.000		0.001	0.000		0.002	0.000	*
Take home sugary drinks	0.000	0.000		0.000	0.000		0.000	0.000		0.000	0.000		0.000	0.000	
Edible ices and ice creams	0.000	0.000		0.000	0.000		0.000	0.000		0.000	0.000		0.000	0.000	*
Dairy products	-0.001	0.000	*	0.000	0.000		0.000	0.000		0.001	0.000	*	0.000	0.000	*
Meat and fish	0.000	0.001		0.000	0.000		0.000	0.000		0.000	0.000		0.000	0.000	
Fats and eggs	-0.001	0.000	*	0.000	0.000		-0.001	0.000	*	0.000	0.000		0.000	0.000	
Fruit	0.000	0.000		0.000	0.000		0.000	0.000		0.000	0.000		0.000	0.000	
Vegetables	-0.003	0.001	*	-0.001	0.000	*	-0.001	0.000		-0.001	0.000	*	0.000	0.000	*
Grains	-0.001	0.000		0.000	0.000		0.000	0.000	*	0.000	0.000		0.000	0.000	
Prepared ready to eat foods	0.001	0.001		0.000	0.000		0.001	0.000	*	0.000	0.001		0.000	0.000	
Sugar and preserves	0.001	0.001		0.001	0.001	*	0.001	0.001	*	0.000	0.001		0.000	0.000	
Condiments and sauces	0.000	0.001		0.001	0.001		0.000	0.001		0.000	0.001		0.000	0.000	
Low calorie, soft drinks and juices	0.000	0.000	*	0.000	0.000		0.000	0.000		0.000	0.000	*	0.000	0.000	
Alcoholic beverages	-0.002	0.001	*	-0.001	0.000	*	0.000	0.000		-0.001	0.000	*	0.000	0.000	*
Numeraire category	-0.004	0.001	*	-0.002	0.001	*	-0.002	0.001	*	-0.001	0.001	*	0.000	0.000	
Log-Likelihood	38143.100														
Obs.	812.000														

Variables	Take home sugary drinks			Edible ices and ice cream			Dairy products			Meat and fish			Fats and eggs		
	Coef.	St. Err.	Sig.	Coef.	St. Err.	Sig.	Coef.	St. Err.	Sig.	Coef.	St. Err.	Sig.	Coef.	St. Err.	Sig.
**Intercept**	0.001	0.020		0.027	0.012	*	0.056	0.025	*	0.084	0.046	*	0.009	0.013	
**Prices**															
Take home confectionery	0.007	0.004		0.000	0.002		-0.003	0.005		-0.004	0.010		-0.002	0.003	
Biscuits	0.009	0.005	*	-0.003	0.004		0.009	0.007		-0.003	0.011		-0.002	0.004	
Take home savouries	0.013	0.005	*	0.007	0.005		0.016	0.007	*	0.000	0.012		0.000	0.005	
Cakes, pastries, and sugar morning goods	-0.001	0.005		0.009	0.003	*	-0.008	0.007		0.005	0.011		0.003	0.004	
Total puddings and desserts	0.000	0.003		0.001	0.003		0.006	0.004		-0.002	0.006		0.000	0.003	
Take home sugary drinks	-0.006	0.008		0.001	0.003		0.009	0.007		-0.020	0.013		-0.008	0.004	*
Edible ices and ice creams	0.001	0.003		-0.006	0.005		-0.005	0.005		-0.021	0.008	*	0.000	0.003	
Dairy products	0.009	0.007		-0.005	0.005		-0.005	0.014		-0.029	0.017	*	0.001	0.005	
Meat and fish	-0.020	0.013		-0.021	0.008	*	-0.029	0.017	*	-0.016	0.040		0.010	0.008	
Fats and eggs	-0.008	0.004	*	0.000	0.003		0.001	0.005		0.010	0.008		0.003	0.004	
Fruit	-0.002	0.008		0.008	0.005		-0.022	0.010	*	0.058	0.017	*	-0.004	0.005	
Vegetables	0.005	0.007		0.001	0.004		-0.004	0.009		-0.001	0.015		0.004	0.005	
Grains	-0.003	0.005		-0.004	0.003		0.009	0.007		-0.005	0.012		-0.002	0.004	
Prepared ready to eat foods	0.008	0.013		0.010	0.009		0.035	0.018	*	0.037	0.029		0.011	0.010	
Sugar and preserves	0.006	0.002	*	0.001	0.001		0.002	0.003		0.007	0.005		0.000	0.002	
Condiments and sauces	0.001	0.002		0.000	0.001		0.004	0.003		-0.003	0.005		-0.001	0.001	
Low calorie, soft drinks and juices	-0.014	0.004	*	-0.001	0.002		-0.003	0.005		0.002	0.010		-0.005	0.003	*
Alcoholic beverages	-0.006	0.003	*	0.001	0.001		-0.004	0.004		-0.001	0.007		-0.003	0.002	
Numeraire category	0.000	-0.099		0.001	-0.068		-0.007	-0.136		-0.014	-0.235		-0.005	-0.073	
**Total expenditure**	0.004	0.002	*	0.000	0.001		-0.012	0.002	*	-0.013	0.004	*	-0.005	0.001	*
**Promotions**															
Take home confectionery	-0.001	0.001		0.000	0.000		-0.001	0.001		-0.002	0.001		0.000	0.000	
Biscuits	0.001	0.001	*	0.001	0.000	*	0.000	0.001		-0.002	0.002		-0.001	0.000	
Take home savouries	0.000	0.000		0.000	0.000		0.001	0.001		-0.004	0.001	*	0.000	0.000	
Cakes, pastries, and sugar morning goods	0.000	0.000		0.000	0.000		0.001	0.000	*	0.000	0.001		0.000	0.000	*
Total puddings and desserts	-0.001	0.000	*	0.000	0.000		-0.001	0.001		0.001	0.001		0.000	0.000	
Take home sugary drinks	0.003	0.000	*	0.000	0.000		-0.001	0.000	*	0.001	0.001	*	0.000	0.000	
Edible ices and ice creams	0.000	0.000		0.002	0.000	*	-0.001	0.000	*	-0.001	0.001		0.000	0.000	
Dairy products	0.000	0.000		0.000	0.000		0.001	0.000	*	-0.001	0.001		0.000	0.000	
Meat and fish	0.000	0.001		0.001	0.000	*	0.000	0.001		0.005	0.001	*	0.000	0.000	
Fats and eggs	0.000	0.000		0.000	0.000		0.000	0.000		0.001	0.001		0.002	0.000	*
Fruit	-0.001	0.000	*	0.000	0.000		0.000	0.001		0.003	0.001	*	0.000	0.000	*
Vegetables	-0.001	0.001		0.000	0.000		0.002	0.001	*	0.000	0.002		0.001	0.000	*
Grains	-0.001	0.000		0.000	0.000		0.001	0.000	*	0.000	0.001		0.000	0.000	
Prepared ready to eat foods	0.001	0.001	*	0.000	0.000		-0.002	0.001	*	-0.001	0.002		-0.001	0.000	*
Sugar and preserves	0.000	0.001		0.000	0.000		0.000	0.001		0.005	0.002	*	0.000	0.000	
Condiments and sauces	0.000	0.001		0.000	0.000		-0.003	0.001	*	0.001	0.002		-0.001	0.000	*
Low calorie, soft drinks and juices	0.000	0.000		0.000	0.000		-0.001	0.000	*	-0.001	0.000	*	0.000	0.000	*
Alcoholic beverages	-0.001	0.001	*	0.000	0.000	*	-0.002	0.001	*	-0.001	0.001		-0.001	0.000	*
Numeraire category	-0.002	0.001	*	-0.001	0.000		-0.002	0.001		0.002	0.002		0.001	0.001	

Variables	Fruit			Vegetables			Grains			Prepared ready to eat foods			Sugar and preserves		
	Coef.	St. Err.	Sig.	Coef.	St. Err.	Sig.	Coef.	St. Err.	Sig.	Coef.	St. Err.	Sig.	Coef.	St. Err.	Sig.
**Intercept**	0.049	0.025	*	0.068	0.023	*	0.062	0.017	*	0.145	0.043	*	0.016	0.008	*
**Prices**															
Take home confectionery	0.005	0.006		0.001	0.005		0.005	0.004		0.006	0.009		-0.004	0.002	*
Biscuits	-0.015	0.007	*	-0.004	0.006		0.008	0.005	*	-0.001	0.012		0.001	0.002	
Take home savouries	-0.011	0.007		-0.002	0.006		-0.001	0.005		-0.004	0.013		0.000	0.002	
Cakes, pastries, and sugar morning goods	-0.008	0.007		-0.003	0.006		-0.001	0.005		0.013	0.012		-0.002	0.002	
Total puddings and desserts	-0.003	0.004		-0.003	0.004		0.004	0.003		0.009	0.007		0.001	0.001	
Take home sugary drinks	-0.002	0.008		0.005	0.007		-0.003	0.005		0.008	0.013		0.006	0.002	*
Edible ices and ice creams	0.008	0.005		0.001	0.004		-0.004	0.003		0.010	0.009		0.001	0.001	
Dairy products	-0.022	0.010	*	-0.004	0.009		0.009	0.007		0.035	0.018	*	0.002	0.003	
Meat and fish	0.058	0.017	*	-0.001	0.015		-0.005	0.012		0.037	0.029		0.007	0.005	
Fats and eggs	-0.004	0.005		0.004	0.005		-0.002	0.004		0.011	0.010		0.000	0.002	
Fruit	-0.005	0.015		0.014	0.009		0.009	0.007		-0.009	0.019		0.000	0.003	
Vegetables	0.014	0.009		-0.003	0.011		-0.004	0.006		0.014	0.016		-0.007	0.003	*
Grains	0.009	0.007		-0.004	0.006		0.000	0.007		-0.007	0.012		-0.003	0.002	
Prepared ready to eat foods	-0.009	0.019		0.014	0.016		-0.007	0.012		-0.128	0.043	*	-0.001	0.005	
Sugar and preserves	0.000	0.003		-0.007	0.003	*	-0.003	0.002		-0.001	0.005		-0.003	0.001	*
Condiments and sauces	-0.005	0.003	*	0.004	0.003		-0.003	0.002		0.008	0.005		0.000	0.001	
Low calorie, soft drinks and juices	-0.006	0.006		0.000	0.005		0.001	0.004		-0.004	0.009		0.004	0.002	*
Alcoholic beverages	-0.001	0.004		-0.005	0.004		0.001	0.003		0.002	0.006		-0.001	0.001	
Numeraire category	-0.003	-0.141		-0.008	-0.124		-0.003	-0.095		0.003	-0.248		-0.001	-0.041	
**Total expenditure**	0.003	0.002		-0.007	0.002	*	-0.012	0.002	*	-0.013	0.003	*	-0.003	0.001	*
**Promotions**															
Take home confectionery	0.000	0.001		-0.001	0.001		0.000	0.001		-0.001	0.001		0.000	0.000	*
Biscuits	0.000	0.001		-0.001	0.001		0.000	0.001		-0.002	0.001		0.000	0.000	
Take home savouries	0.000	0.001		0.000	0.001		0.000	0.000		0.000	0.001		0.001	0.000	*
Cakes, pastries, and sugar morning goods	0.000	0.001		0.001	0.000	*	0.000	0.000		-0.001	0.001		0.000	0.000	*
Total puddings and desserts	0.001	0.001		-0.001	0.001		0.000	0.000		0.000	0.001		0.000	0.000	*
Take home sugary drinks	-0.001	0.000	*	-0.001	0.000	*	0.000	0.000		0.001	0.001		0.000	0.000	
Edible ices and ice creams	0.001	0.001		-0.001	0.000		-0.001	0.000	*	-0.003	0.001	*	0.000	0.000	
Dairy products	0.000	0.000		0.000	0.000		0.001	0.000	*	-0.001	0.000		0.000	0.000	
Meat and fish	-0.001	0.001		0.001	0.001	*	0.001	0.000	*	-0.002	0.001	*	0.000	0.000	
Fats and eggs	0.001	0.000	*	0.000	0.000		0.000	0.000		0.001	0.001		0.000	0.000	
Fruit	0.008	0.001	*	0.001	0.001	*	-0.001	0.000	*	-0.001	0.001		-0.001	0.000	*
Vegetables	-0.001	0.001		0.007	0.001	*	0.002	0.001	*	-0.002	0.001		0.000	0.000	
Grains	0.001	0.001		-0.001	0.000	*	0.002	0.000	*	0.000	0.001		0.000	0.000	
Prepared ready to eat foods	-0.004	0.001	*	-0.001	0.001		-0.001	0.001		0.015	0.002	*	0.000	0.000	
Sugar and preserves	-0.003	0.001	*	-0.002	0.001	*	0.000	0.001		0.002	0.002		0.002	0.000	*
Condiments and sauces	-0.003	0.001	*	-0.002	0.001	*	0.001	0.001		0.002	0.002		-0.001	0.000	*
Low calorie, soft drinks and juices	-0.001	0.000	*	-0.001	0.000	*	0.000	0.000		0.000	0.000		0.000	0.000	
Alcoholic beverages	-0.002	0.001	*	-0.001	0.001	*	-0.001	0.000	*	-0.002	0.001	*	-0.001	0.000	*
Numeraire category	-0.002	0.001		0.000	0.001		-0.001	0.001		0.003	0.002		0.000	0.000	

Variables	Condiments and sauces			Low calorie soft drinks and juices			Alcoholic beverages			Numeraire category		
	Coef.	St. Err.	Sig.	Coef.	St. Err.	Sig.	Coef.	St. Err.	Sig.	Coef.	St. Err.	Sig.
**Intercept**	0.018	0.007	*	0.032	0.014	*	0.049	0.014	*	0.190	-0.343	
**Prices**												
Take home confectionery	-0.001	0.002		0.002	0.004		0.000	0.003		0.012	-0.076	
Biscuits	-0.001	0.002		0.008	0.003	*	-0.001	0.002		-0.006	-0.096	
Take home savouries	0.002	0.002		-0.002	0.003		-0.002	0.002		0.002	-0.103	
Cakes, pastries, and sugar morning goods	0.001	0.002		0.007	0.003	*	0.001	0.002		-0.001	-0.090	
Total puddings and desserts	0.001	0.001		-0.001	0.002		-0.001	0.001		0.000	-0.056	
Take home sugary drinks	0.001	0.002		-0.014	0.004	*	-0.006	0.003	*	0.000	-0.099	
Edible ices and ice creams	0.000	0.001		-0.001	0.002		0.001	0.001		0.001	-0.068	
Dairy products	0.004	0.003		-0.003	0.005		-0.004	0.004		-0.007	-0.136	
Meat and fish	-0.003	0.005		0.002	0.010		-0.001	0.007		-0.014	-0.235	
Fats and eggs	-0.001	0.001		-0.005	0.003	*	-0.003	0.002		-0.005	-0.073	
Fruit	-0.005	0.003	*	-0.006	0.006		-0.001	0.004		-0.003	-0.141	
Vegetables	0.004	0.003		0.000	0.005		-0.005	0.004		-0.008	-0.124	
Grains	-0.003	0.002		0.001	0.004		0.001	0.003		-0.003	-0.095	
Prepared ready to eat foods	0.008	0.005		-0.004	0.009		0.002	0.006		0.003	-0.248	
Sugar and preserves	0.000	0.001		0.004	0.002	*	-0.001	0.001		-0.001	-0.041	
Condiments and sauces	-0.001	0.001		-0.002	0.002		0.000	0.001		-0.003	-0.039	
Low calorie, soft drinks and juices	-0.002	0.002		0.007	0.005		0.001	0.003		0.006	-0.075	
Alcoholic beverages	0.000	0.001		0.001	0.003		0.014	0.008		0.005	-0.057	
Numeraire category	-0.003	-0.039		0.006	-0.075		0.005	-0.057		0.021	1.852	
**Total expenditure**	-0.005	0.001	*	0.004	0.002	*	-0.004	0.005		0.056	-0.032	*
**Promotions**												
Take home confectionery	0.000	0.000		-0.001	0.001		-0.001	0.002		-0.001	-0.012	
Biscuits	0.000	0.000		-0.001	0.001		0.003	0.002		-0.002	-0.014	
Take home savouries	0.000	0.000		0.001	0.000	*	0.001	0.001		0.000	-0.009	
Cakes, pastries, and sugar morning goods	0.000	0.000		0.000	0.000		-0.001	0.001		-0.002	-0.008	
Total puddings and desserts	0.000	0.000		0.000	0.001		0.000	0.001		-0.002	-0.009	
Take home sugary drinks	0.000	0.000		0.000	0.000		-0.001	0.001		-0.001	-0.006	
Edible ices and ice creams	-0.001	0.000	*	0.001	0.000		0.004	0.001	*	0.000	-0.008	
Dairy products	0.000	0.000	*	0.000	0.000		0.000	0.001		0.000	-0.005	
Meat and fish	0.000	0.000		-0.001	0.001	*	-0.003	0.001	*	0.000	-0.010	
Fats and eggs	0.000	0.000		-0.001	0.000	*	0.000	0.001		-0.001	-0.006	
Fruit	0.000	0.000		-0.001	0.000	*	-0.004	0.001	*	-0.002	-0.008	
Vegetables	0.000	0.000		0.000	0.001		-0.001	0.002		-0.002	-0.013	
Grains	0.000	0.000		-0.001	0.000	*	-0.001	0.001		0.001	-0.007	
Prepared ready to eat foods	0.000	0.000	*	-0.002	0.001	*	-0.005	0.002	*	0.000	-0.014	
Sugar and preserves	0.000	0.000		0.000	0.001		-0.003	0.002		-0.002	-0.016	
Condiments and sauces	0.003	0.000	*	0.001	0.001		0.000	0.003		0.001	-0.017	
Low calorie, soft drinks and juices	0.000	0.000		0.002	0.000	*	0.001	0.001	*	0.001	-0.004	
Alcoholic beverages	-0.001	0.000	*	-0.001	0.001		0.016	0.001	*	0.000	-0.010	
Numeraire category	0.000	0.000		0.002	0.001	*	0.000	0.003		0.010	-0.019	

Note: '*' indicates statistically significant at 5 per cent.

Table S10 - Scotland - Older family group - All categories - Demand model results

Equations (columns 1 of 4)

Variables	Take home confectionery			Biscuits			Take home savouries			Cakes, pastries and sugar morning goods		
	Coef.	St. Err.	Sig.	Coef.	St. Err.	Sig.	Coef.	St. Err.	Sig.	Coef.	St. Err.	Sig.
**Intercept**	0.030	0.015	*	-0.026	0.016		-0.012	0.017		0.006	0.016	
**Prices**												
Take home confectionery	-0.001	0.005		0.000	0.003		-0.003	0.003		-0.003	0.003	
Biscuits	0.000	0.003		0.008	0.008		0.009	0.007		-0.013	0.005	*
Take home savouries	-0.003	0.003		0.009	0.007		0.010	0.010		0.007	0.005	
Cakes, pastries, and sugar morning goods	-0.003	0.003		-0.013	0.005	*	0.007	0.005		-0.011	0.006	*
Total puddings and desserts	0.000	0.002		0.003	0.004		0.004	0.004		-0.001	0.003	
Take home sugary drinks	-0.004	0.004		0.003	0.004		-0.004	0.004		0.008	0.005	*
Edible ices and ice creams	-0.004	0.002	*	0.000	0.004		-0.001	0.005		0.001	0.003	
Dairy products	0.012	0.006	*	-0.015	0.007	*	-0.004	0.007		-0.018	0.007	*
Meat and fish	0.025	0.010	*	0.036	0.011	*	0.016	0.011		0.037	0.011	*
Fats and eggs	0.006	0.003	*	0.002	0.004		-0.009	0.004	*	-0.007	0.004	*
Fruit	-0.006	0.005		0.001	0.005		-0.011	0.005	*	-0.008	0.005	
Vegetables	-0.008	0.005	*	-0.018	0.006	*	0.006	0.006		0.000	0.006	
Grains	0.002	0.004		-0.001	0.004		-0.004	0.004		0.002	0.004	
Prepared ready to eat foods	-0.012	0.009		-0.012	0.011		-0.022	0.012	*	0.000	0.011	
Sugar and preserves	0.000	0.002		0.000	0.002		0.003	0.002		0.001	0.002	
Condiments and sauces	-0.003	0.002		0.000	0.002		0.000	0.002		-0.001	0.002	
Low calorie, soft drinks and juices	-0.002	0.004		0.000	0.003		0.001	0.003		0.010	0.003	*
Alcoholic beverages	0.001	0.003		-0.002	0.002		0.000	0.002		-0.003	0.002	
Numeraire category	-0.001	-0.073		0.001	-0.091		0.001	-0.097		-0.001	-0.086	
**Total expenditure**	0.008	0.002	*	-0.001	0.001		-0.002	0.001	*	0.001	0.001	
**Promotions**												
Take home confectionery	0.005	0.000	*	-0.001	0.000	*	0.000	0.000		0.000	0.000	
Biscuits	-0.001	0.000	*	0.003	0.000	*	0.000	0.000		0.001	0.000	*
Take home savouries	-0.001	0.000	*	0.000	0.000		0.002	0.000	*	0.000	0.000	
Cakes, pastries, and sugar morning goods	-0.001	0.000		0.000	0.000		0.000	0.000		0.001	0.000	*
Total puddings and desserts	0.000	0.000		0.001	0.000	*	0.000	0.000		0.001	0.000	*
Take home sugary drinks	0.000	0.000		0.000	0.000		0.000	0.000	*	0.000	0.000	*
Edible ices and ice creams	0.001	0.000	*	0.000	0.000		0.000	0.000	*	0.000	0.000	
Dairy products	0.000	0.000		0.000	0.000	*	0.000	0.000		0.000	0.000	*
Meat and fish	-0.001	0.001		0.000	0.000		0.000	0.000		0.000	0.000	
Fats and eggs	0.000	0.000		0.001	0.000	*	0.000	0.000		0.000	0.000	
Fruit	0.000	0.001		-0.001	0.000	*	-0.001	0.000	*	0.000	0.000	
Vegetables	-0.001	0.001	*	0.000	0.000		0.000	0.000		-0.002	0.001	*
Grains	-0.001	0.000	*	0.000	0.000		0.000	0.000		0.000	0.000	
Prepared ready to eat foods	0.002	0.001	*	0.000	0.000		0.001	0.000	*	0.000	0.000	
Sugar and preserves	0.002	0.001	*	0.000	0.000		0.000	0.000		0.000	0.000	
Condiments and sauces	-0.001	0.001		-0.001	0.000	*	0.001	0.000		0.001	0.001	*
Low calorie, soft drinks and juices	0.000	0.000		0.000	0.000		0.000	0.000		0.000	0.000	*
Alcoholic beverages	-0.001	0.001	*	-0.001	0.000	*	0.000	0.000		-0.001	0.000	*
Numeraire category	-0.002	0.001	*	-0.001	0.000	*	-0.001	0.000	*	-0.001	0.001	*
Log-Likelihood	40855.200											
Obs.	887.000											

Equations (columns 2 of 4)

Variables	Total puddings and desserts			Take home sugary drinks			Edible ices and ice cream			Dairy products			Meat and fish		
	Coef.	St. Err.	Sig.	Coef.	St. Err.	Sig.	Coef.	St. Err.	Sig.	Coef.	St. Err.	Sig.	Coef.	St. Err.	Sig.
**Intercept**	0.016	0.010		0.037	0.021	*	0.017	0.011		0.121	0.026	*	0.220	0.047	*
**Prices**															
Take home confectionery	0.000	0.002		-0.004	0.004		-0.004	0.002	*	0.012	0.006	*	0.025	0.010	*
Biscuits	0.003	0.004		0.003	0.004		0.000	0.004		-0.015	0.007	*	0.036	0.011	*
Take home savouries	0.004	0.004		-0.004	0.004		-0.001	0.005		-0.004	0.007		0.016	0.011	
Cakes, pastries, and sugar morning goods	-0.001	0.003		0.008	0.005	*	0.001	0.003		-0.018	0.007	*	0.037	0.011	*
Total puddings and desserts	-0.003	0.003		0.004	0.003		-0.003	0.003		-0.007	0.004	*	-0.011	0.007	
Take home sugary drinks	0.004	0.003		-0.009	0.008		0.000	0.003		0.017	0.007	*	-0.013	0.013	
Edible ices and ice creams	-0.003	0.003		0.000	0.003		0.000	0.004		-0.001	0.005		-0.015	0.007	*
Dairy products	-0.007	0.004	*	0.017	0.007	*	-0.001	0.005		0.018	0.015		-0.008	0.018	
Meat and fish	-0.011	0.007		-0.013	0.013		-0.015	0.007	*	-0.008	0.018		-0.067	0.042	
Fats and eggs	0.000	0.003		0.004	0.003		0.002	0.003		-0.003	0.005		0.014	0.009	
Fruit	-0.001	0.003		0.010	0.007		0.004	0.003		0.003	0.009		0.010	0.015	
Vegetables	-0.010	0.004	*	0.005	0.006		0.004	0.004		-0.010	0.009		0.000	0.015	
Grains	0.005	0.003	*	-0.001	0.005		-0.004	0.003		0.006	0.007		0.013	0.012	
Prepared ready to eat foods	0.020	0.007	*	-0.030	0.012	*	0.010	0.008		0.019	0.017		-0.019	0.029	
Sugar and preserves	0.000	0.001		0.001	0.002		0.000	0.001		-0.005	0.003		0.003	0.006	
Condiments and sauces	-0.001	0.001		0.002	0.002		0.001	0.001		0.000	0.003		-0.004	0.005	
Low calorie, soft drinks and juices	0.000	0.002		0.010	0.004	*	0.007	0.002	*	-0.002	0.005		-0.016	0.010	*
Alcoholic beverages	0.000	0.001		-0.002	0.003		0.000	0.001		0.000	0.003		0.005	0.006	
Numeraire category	0.001	-0.057		0.000	-0.096		0.000	-0.062		-0.003	-0.137		-0.006	-0.238	
**Total expenditure**	-0.001	0.001		0.005	0.002	*	-0.002	0.001	*	-0.008	0.002	*	-0.011	0.004	*
**Promotions**															
Take home confectionery	0.000	0.000		0.000	0.001		0.000	0.000		0.000	0.001		0.000	0.001	
Biscuits	0.000	0.000		0.000	0.000		0.000	0.000		0.000	0.001		-0.002	0.001	*
Take home savouries	0.000	0.000		-0.001	0.000		0.000	0.000	*	0.001	0.000		0.000	0.001	
Cakes, pastries, and sugar morning goods	0.001	0.000	*	0.001	0.000		0.000	0.000		0.002	0.001	*	-0.002	0.001	
Total puddings and desserts	0.003	0.000	*	0.000	0.000		0.001	0.000	*	0.002	0.000	*	-0.002	0.001	*
Take home sugary drinks	0.000	0.000		0.003	0.000	*	0.000	0.000	*	-0.001	0.000	*	0.000	0.000	
Edible ices and ice creams	0.000	0.000		0.000	0.000		0.003	0.000	*	0.000	0.000		-0.002	0.001	*
Dairy products	0.000	0.000	*	0.000	0.000	*	0.000	0.000		0.002	0.000	*	-0.002	0.000	*
Meat and fish	0.000	0.000		0.000	0.001		0.000	0.000		-0.001	0.001		0.008	0.001	*
Fats and eggs	0.000	0.000	*	-0.001	0.000		0.000	0.000		0.000	0.000		0.002	0.001	*
Fruit	0.000	0.000		-0.001	0.001		0.000	0.000		0.002	0.001	*	-0.002	0.001	*
Vegetables	-0.001	0.000	*	0.000	0.001		0.000	0.000		-0.001	0.001	*	0.004	0.002	*
Grains	0.000	0.000	*	0.000	0.000		0.000	0.000		0.001	0.001		-0.001	0.001	
Prepared ready to eat foods	0.000	0.000	*	0.000	0.001		0.000	0.000		0.000	0.001		-0.003	0.002	*
Sugar and preserves	0.000	0.000		0.000	0.001		0.000	0.000		-0.001	0.001	*	-0.002	0.002	
Condiments and sauces	0.000	0.000		0.000	0.001		0.000	0.000		-0.001	0.001		-0.001	0.002	
Low calorie, soft drinks and juices	0.000	0.000		-0.001	0.000	*	0.000	0.000		-0.001	0.000	*	0.000	0.000	
Alcoholic beverages	0.000	0.000	*	0.000	0.001		-0.001	0.000	*	-0.002	0.001	*	-0.004	0.001	*
Numeraire category	0.000	0.000		-0.003	0.001	*	-0.001	0.000	*	-0.002	0.001	*	0.001	0.002	
Log-Likelihood															
Obs.															

Equations (columns 3 of 4)

Variables	Fats and eggs			Fruit			Vegetables			Grains			Prepared ready to eat foods		
	Coef.	St. Err.	Sig.	Coef.	St. Err.	Sig.	Coef.	St. Err.	Sig.	Coef.	St. Err.	Sig.	Coef.	St. Err.	Sig.
**Intercept**	0.019	0.013		0.058	0.022	*	0.062	0.023	*	0.055	0.016	*	0.032	0.041	
**Prices**															
Take home confectionery	0.006	0.003	*	-0.006	0.005		-0.008	0.005	*	0.002	0.004		-0.012	0.009	
Biscuits	0.002	0.004		0.001	0.005		-0.018	0.006	*	-0.001	0.004		-0.012	0.011	
Take home savouries	-0.009	0.004	*	-0.011	0.005	*	0.006	0.006		-0.004	0.004		-0.022	0.012	*
Cakes, pastries, and sugar morning goods	-0.007	0.004	*	-0.008	0.005		0.000	0.006		0.002	0.004		0.000	0.011	
Total puddings and desserts	0.000	0.003		-0.001	0.003		-0.010	0.004	*	0.005	0.003	*	0.020	0.007	*
Take home sugary drinks	0.004	0.003		0.010	0.007		0.005	0.006		-0.001	0.005		-0.030	0.012	*
Edible ices and ice creams	0.002	0.003		0.004	0.003		0.004	0.004		-0.004	0.003		0.010	0.008	
Dairy products	-0.003	0.005		0.003	0.009		-0.010	0.009		0.006	0.007		0.019	0.017	
Meat and fish	0.014	0.009		0.010	0.015		0.000	0.015		0.013	0.012		-0.019	0.029	
Fats and eggs	0.003	0.004		0.006	0.004		-0.001	0.005		-0.007	0.003	*	-0.010	0.009	
Fruit	0.006	0.004		0.004	0.011		0.010	0.008		-0.003	0.006		-0.009	0.014	
Vegetables	-0.001	0.005		0.010	0.008		-0.007	0.011		0.007	0.006		0.012	0.015	
Grains	-0.007	0.003	*	-0.003	0.006		0.007	0.006		-0.026	0.006	*	0.016	0.011	
Prepared ready to eat foods	-0.010	0.009		-0.009	0.014		0.012	0.015		0.016	0.011		0.031	0.039	
Sugar and preserves	0.000	0.002		0.002	0.003		0.000	0.003		-0.007	0.002	*	0.006	0.005	
Condiments and sauces	0.000	0.002		0.000	0.003		0.003	0.003		0.002	0.002		0.006	0.005	
Low calorie, soft drinks and juices	0.001	0.002		-0.004	0.005		0.005	0.005		0.003	0.004		0.004	0.008	
Alcoholic beverages	0.000	0.001		-0.005	0.004		0.001	0.003		-0.004	0.002		-0.005	0.005	
Numeraire category	-0.003	-0.070		-0.004	-0.116		0.001	-0.117		0.001	-0.088		-0.006	-0.228	
**Total expenditure**	-0.005	0.001	*	-0.005	0.002	*	-0.004	0.002	*	-0.009	0.001	*	-0.001	0.003	
**Promotions**															
Take home confectionery	0.000	0.000		-0.001	0.001		0.000	0.001		-0.001	0.000	*	0.000	0.001	
Biscuits	0.000	0.000	*	0.001	0.001		0.000	0.000		0.000	0.000		0.000	0.001	
Take home savouries	0.000	0.000	*	0.000	0.000		0.000	0.000		0.000	0.000		-0.001	0.001	
Cakes, pastries, and sugar morning goods	0.001	0.000	*	-0.001	0.001		0.000	0.000		0.000	0.000		-0.001	0.001	
Total puddings and desserts	0.000	0.000	*	0.000	0.000		-0.001	0.000	*	0.001	0.000	*	0.000	0.001	
Take home sugary drinks	0.000	0.000	*	-0.001	0.000	*	-0.001	0.000	*	0.000	0.000	*	0.001	0.000	*
Edible ices and ice creams	0.000	0.000		0.002	0.000	*	0.000	0.000		0.000	0.000		0.001	0.001	*
Dairy products	0.000	0.000		0.000	0.000		0.000	0.000		0.000	0.000	*	0.000	0.000	
Meat and fish	0.000	0.000		-0.002	0.001	*	-0.001	0.001		-0.001	0.000		-0.001	0.001	
Fats and eggs	0.001	0.000	*	0.000	0.000		0.000	0.000		0.001	0.000	*	0.000	0.001	
Fruit	-0.001	0.000	*	0.007	0.001	*	0.001	0.001		-0.001	0.000	*	-0.002	0.001	*
Vegetables	0.001	0.000	*	0.000	0.001		0.007	0.001	*	0.000	0.001		-0.003	0.001	*
Grains	0.000	0.000		0.001	0.001		-0.001	0.000		0.003	0.000	*	-0.001	0.001	
Prepared ready to eat foods	0.000	0.000		-0.003	0.001	*	-0.003	0.001	*	0.001	0.001		0.008	0.001	*
Sugar and preserves	0.000	0.000		-0.003	0.001	*	0.000	0.001		-0.001	0.001		0.002	0.001	*
Condiments and sauces	0.000	0.000		0.000	0.001		0.000	0.001		0.001	0.001		0.005	0.001	*
Low calorie, soft drinks and juices	0.000	0.000		-0.001	0.000	*	0.000	0.000	*	0.000	0.000	*	0.000	0.000	
Alcoholic beverages	0.000	0.000	*	-0.001	0.001		-0.001	0.001		-0.002	0.000	*	-0.003	0.001	*
Numeraire category	0.001	0.000		-0.002	0.001	*	0.000	0.001		0.000	0.001		-0.002	0.001	
Log-Likelihood															
Obs.															

Equations (columns 4 of 4)

Variables	Sugar and preserves			Condiments and sauces			Low calorie soft drinks and juices			Alcoholic beverages			Numeraire category		
	Coef.	St. Err.	Sig.	Coef.	St. Err.	Sig.	Coef.	St. Err.	Sig.	Coef.	St. Err.	Sig.	Coef.	St. Err.	Sig.
**Intercept**	0.009	0.008		0.032	0.008	*	0.084	0.014	*	0.089	0.014	*	0.153	0.091	*
**Prices**															
Take home confectionery	0.000	0.002		-0.003	0.002		-0.002	0.004		0.001	0.003		-0.001	0.020	
Biscuits	0.000	0.002		0.000	0.002		0.000	0.003		-0.002	0.002		0.001	0.024	
Take home savouries	0.003	0.002		0.000	0.002		0.001	0.003		0.000	0.002		0.001	0.026	
Cakes, pastries, and sugar morning goods	0.001	0.002		-0.001	0.002		0.010	0.003	*	-0.003	0.002		-0.001	0.023	
Total puddings and desserts	0.000	0.001		-0.001	0.001		0.000	0.002		0.000	0.001		0.001	0.015	
Take home sugary drinks	0.001	0.002		0.002	0.002		0.010	0.004	*	-0.002	0.003		0.000	0.026	
Edible ices and ice creams	0.000	0.001		0.001	0.001		0.007	0.002	*	0.000	0.001		0.000	0.017	
Dairy products	-0.005	0.003		0.000	0.003		-0.002	0.005		0.000	0.003		-0.003	0.037	
Meat and fish	0.003	0.006		-0.004	0.005		-0.016	0.010	*	0.005	0.006		-0.006	0.068	
Fats and eggs	0.000	0.002		0.000	0.002		0.001	0.002		0.000	0.001		-0.003	0.019	
Fruit	0.002	0.003		0.000	0.003		-0.004	0.005		-0.005	0.004		-0.004	0.031	
Vegetables	0.000	0.003		0.003	0.003		0.005	0.005		0.001	0.003		0.001	0.031	
Grains	-0.007	0.002	*	0.002	0.002		0.003	0.004		-0.004	0.002		0.001	0.024	
Prepared ready to eat foods	0.006	0.005		0.006	0.005		0.004	0.008		-0.005	0.005		-0.006	0.064	
Sugar and preserves	-0.001	0.001		0.000	0.001		0.000	0.002		-0.001	0.001		-0.002	0.011	
Condiments and sauces	0.000	0.001		-0.003	0.001	*	-0.001	0.002		-0.001	0.001		0.000	0.011	
Low calorie, soft drinks and juices	0.000	0.002		-0.001	0.002		-0.019	0.005	*	0.000	0.003		0.004	0.019	
Alcoholic beverages	-0.001	0.001		-0.001	0.001		0.000	0.003		0.009	0.008		0.007	0.014	
Numeraire category	-0.002	-0.041		0.000	-0.039		0.004	-0.071		0.007	-0.051		0.011	0.474	
**Total expenditure**	-0.002	0.001	*	-0.001	0.001	*	0.002	0.002		-0.008	0.005		0.044	0.009	*
**Promotions**															
Take home confectionery	0.000	0.000		0.000	0.000	*	0.000	0.000		-0.002	0.001		0.000	0.003	
Biscuits	0.000	0.000	*	0.000	0.000		0.000	0.000		0.001	0.001		-0.001	0.002	
Take home savouries	0.000	0.000		0.000	0.000		0.000	0.000		-0.001	0.001		-0.002	0.002	
Cakes, pastries, and sugar morning goods	0.000	0.000		0.000	0.000		0.000	0.000		-0.001	0.001		-0.001	0.002	
Total puddings and desserts	0.000	0.000		0.000	0.000	*	0.001	0.000	*	-0.004	0.001	*	0.001	0.002	
Take home sugary drinks	0.000	0.000		0.000	0.000		0.000	0.000	*	0.000	0.001		0.000	0.001	
Edible ices and ice creams	0.000	0.000		0.000	0.000		-0.001	0.000	*	-0.003	0.001	*	-0.001	0.002	
Dairy products	0.000	0.000	*	0.000	0.000		0.000	0.000		-0.001	0.001		0.000	0.001	
Meat and fish	-0.001	0.000	*	0.000	0.000		0.000	0.001		0.000	0.002		-0.002	0.003	
Fats and eggs	0.000	0.000		0.000	0.000		-0.001	0.000	*	-0.001	0.001		-0.001	0.002	
Fruit	-0.001	0.000	*	0.000	0.000		0.000	0.000		-0.003	0.001	*	0.000	0.003	
Vegetables	0.000	0.000		0.000	0.000		-0.001	0.001	*	0.003	0.002		-0.002	0.004	
Grains	0.000	0.000		0.000	0.000		0.000	0.000		0.000	0.001		0.000	0.002	
Prepared ready to eat foods	0.000	0.000		-0.001	0.000	*	0.000	0.001		-0.003	0.002	*	0.000	0.003	
Sugar and preserves	0.003	0.000	*	0.000	0.000		0.000	0.001		-0.003	0.002		0.003	0.003	
Condiments and sauces	0.000	0.000		0.002	0.000	*	0.000	0.001		-0.002	0.002		-0.003	0.004	
Low calorie, soft drinks and juices	0.000	0.000		0.000	0.000		0.003	0.000	*	0.001	0.001		0.000	0.001	
Alcoholic beverages	-0.001	0.000	*	0.000	0.000		-0.002	0.000	*	0.020	0.001	*	-0.001	0.003	
Numeraire category	0.000	0.000		0.001	0.000	*	-0.002	0.001	*	-0.005	0.002	*	0.019	0.004	*
Log-Likelihood															
Obs.															

Note: '*' indicates statistically significant at 5 per cent.

Table S11 - Scotland - 45+ no children group - All categories - Demand model results

Equations (columns 1–5 of 19)

Variables	Take home confectionery			Biscuits			Take home savouries			Cakes, pastries and sugar morning goods			Total puddings and desserts		
	Coef.	St. Err.	Sig.	Coef.	St. Err.	Sig.	Coef.	St. Err.	Sig.	Coef.	St. Err.	Sig.	Coef.	St. Err.	Sig.
**Intercept**	0.039	0.005	*	0.008	0.007		0.025	0.006	*	0.024	0.007	*	0.009	0.004	*
**Prices**															
Take home confectionery	-0.011	0.002	*	-0.004	0.001	*	-0.007	0.001	*	0.003	0.001	*	-0.001	0.001	*
Biscuits	-0.004	0.001	*	-0.002	0.004		0.007	0.003	*	0.004	0.002	*	-0.006	0.002	*
Take home savouries	-0.007	0.001	*	0.007	0.003	*	-0.004	0.003		-0.001	0.002		0.004	0.001	*
Cakes, pastries, and sugar morning goo	0.003	0.001	*	0.004	0.002	*	-0.001	0.002		-0.002	0.003		-0.001	0.001	
Total puddings and desserts	-0.001	0.001	*	-0.006	0.002	*	0.004	0.001	*	-0.001	0.001		-0.005	0.001	*
Take home sugary drinks	-0.006	0.001	*	-0.007	0.002	*	0.003	0.002	*	0.000	0.002		-0.002	0.001	*
Edible ices and ice creams	0.000	0.001		0.002	0.002		-0.003	0.002	*	0.002	0.001		0.001	0.001	
Dairy products	0.007	0.002	*	-0.001	0.003		0.006	0.003	*	0.003	0.003		-0.003	0.002	
Meat and fish	-0.006	0.004		0.000	0.005		-0.008	0.004	*	0.005	0.005		0.003	0.003	
Fats and eggs	0.001	0.001		-0.002	0.002		0.005	0.002	*	0.001	0.002		0.001	0.001	
Fruit	0.018	0.002	*	0.005	0.003	*	-0.004	0.002	*	0.002	0.003		-0.002	0.002	
Vegetables	0.009	0.002	*	-0.003	0.003		0.005	0.002	*	0.005	0.003	*	0.002	0.002	
Grains	-0.003	0.001	*	0.005	0.002	*	0.003	0.002	*	0.003	0.002		-0.003	0.001	*
Prepared ready to eat foods	-0.003	0.004		-0.001	0.005		-0.003	0.005		-0.019	0.005	*	0.011	0.003	*
Sugar and preserves	0.001	0.001		0.004	0.001	*	0.000	0.001		0.001	0.001		0.000	0.001	
Condiments and sauces	0.000	0.001		0.000	0.001		-0.001	0.001		0.001	0.001		0.000	0.000	
Low calorie, soft drinks and juices	-0.001	0.001		0.002	0.001		0.000	0.001		-0.006	0.001	*	0.001	0.001	
Alcoholic beverages	0.003	0.001	*	-0.001	0.001		0.000	0.001		0.000	0.001		0.000	0.000	
Numeraire category	0.000	-0.029		-0.001	-0.041		-0.002	-0.036		-0.002	-0.037		0.000	-0.025	
**Total expenditure**	0.004	0.001	*	-0.003	0.000	*	-0.003	0.000	*	0.000	0.000		0.000	0.000	
**Promotions**															
Take home confectionery	0.005	0.000	*	0.000	0.000	*	0.000	0.000	*	0.001	0.000	*	0.000	0.000	*
Biscuits	0.000	0.000		0.004	0.000	*	0.000	0.000	*	0.001	0.000	*	0.000	0.000	
Take home savouries	0.000	0.000		0.000	0.000		0.002	0.000	*	0.000	0.000		0.000	0.000	
Cakes, pastries, and sugar morning goo	0.000	0.000		0.000	0.000	*	0.000	0.000		0.001	0.000	*	0.000	0.000	
Total puddings and desserts	0.001	0.000	*	0.000	0.000	*	0.000	0.000		0.000	0.000	*	0.003	0.000	*
Take home sugary drinks	0.000	0.000		0.000	0.000		0.000	0.000	*	0.000	0.000		0.000	0.000	*
Edible ices and ice creams	0.001	0.000	*	0.000	0.000		0.000	0.000	*	0.000	0.000		0.000	0.000	*
Dairy products	0.000	0.000		0.000	0.000		0.000	0.000		0.000	0.000		0.000	0.000	*
Meat and fish	-0.001	0.000	*	0.000	0.000	*	0.000	0.000	*	0.000	0.000		0.000	0.000	*
Fats and eggs	0.000	0.000	*	0.000	0.000		0.000	0.000	*	0.000	0.000		0.000	0.000	*
Fruit	0.000	0.000	*	0.000	0.000		0.000	0.000	*	-0.001	0.000	*	0.000	0.000	
Vegetables	-0.001	0.000	*	-0.001	0.000	*	0.000	0.000	*	0.000	0.000	*	0.000	0.000	*
Grains	0.000	0.000		0.000	0.000		0.000	0.000	*	0.000	0.000		0.000	0.000	*
Prepared ready to eat foods	-0.001	0.000	*	0.000	0.000		0.000	0.000		0.000	0.000		0.000	0.000	
Sugar and preserves	0.000	0.000	*	0.000	0.000		0.000	0.000	*	0.001	0.000	*	0.000	0.000	*
Condiments and sauces	0.000	0.000		-0.001	0.000	*	0.001	0.000	*	-0.001	0.000	*	0.000	0.000	*
Low calorie, soft drinks and juices	0.000	0.000		0.000	0.000	*	0.000	0.000	*	0.000	0.000	*	0.000	0.000	*
Alcoholic beverages	-0.002	0.000	*	-0.001	0.000	*	-0.001	0.000	*	-0.001	0.000	*	0.000	0.000	*
Numeraire category	-0.002	0.000	*	-0.001	0.000	*	0.000	0.000	*	-0.001	0.000	*	0.000	0.000	*
Log-Likelihood	248801.000														
Obs.	5699.000														

Equations (columns 6–10 of 19)

Variables	Take home sugary drinks			Edible ices and ice cream			Dairy products			Meat and fish			Fats and eggs		
	Coef.	St. Err.	Sig.	Coef.	St. Err.	Sig.	Coef.	St. Err.	Sig.	Coef.	St. Err.	Sig.	Coef.	St. Err.	Sig.
**Intercept**	0.016	0.006	*	0.008	0.005	*	0.069	0.010	*	0.146	0.017	*	0.032	0.006	*
**Prices**															
Take home confectionery	-0.006	0.001	*	0.000	0.001		0.007	0.002	*	-0.006	0.004		0.001	0.001	
Biscuits	-0.007	0.002	*	0.002	0.002		-0.001	0.003		0.000	0.005		-0.002	0.002	
Take home savouries	0.003	0.002	*	-0.003	0.002	*	0.006	0.003	*	-0.008	0.004	*	0.005	0.002	*
Cakes, pastries, and sugar morning goo	0.000	0.002		0.002	0.001		0.003	0.003		0.005	0.005		0.001	0.002	
Total puddings and desserts	-0.002	0.001	*	0.001	0.001		-0.003	0.002		0.003	0.003		0.001	0.001	
Take home sugary drinks	-0.006	0.002	*	-0.003	0.001	*	0.002	0.003		0.001	0.004		0.001	0.001	
Edible ices and ice creams	-0.003	0.001	*	0.000	0.002		-0.002	0.002		0.005	0.003		0.004	0.001	*
Dairy products	0.002	0.003		-0.002	0.002		-0.036	0.006	*	0.019	0.007	*	0.004	0.002	
Meat and fish	0.001	0.004		0.005	0.003		0.019	0.007	*	-0.031	0.016	*	-0.007	0.004	*
Fats and eggs	0.001	0.001		0.004	0.001	*	0.004	0.002		-0.007	0.004	*	0.005	0.002	*
Fruit	0.007	0.003	*	0.002	0.002		0.001	0.004		0.002	0.007		-0.011	0.002	*
Vegetables	0.006	0.002	*	0.001	0.002		-0.006	0.004		-0.019	0.006	*	-0.008	0.002	*
Grains	-0.002	0.002		-0.003	0.001	*	-0.002	0.003		0.016	0.004	*	-0.001	0.002	
Prepared ready to eat foods	0.004	0.004		-0.007	0.004	*	0.007	0.007		0.032	0.012	*	0.005	0.004	
Sugar and preserves	0.000	0.001		-0.001	0.001		-0.002	0.001		0.003	0.002		-0.001	0.001	
Condiments and sauces	-0.001	0.001		0.000	0.001		-0.001	0.001		0.000	0.002		0.001	0.001	*
Low calorie, soft drinks and juices	0.002	0.001		0.003	0.001	*	0.009	0.002	*	-0.010	0.004	*	0.002	0.001	
Alcoholic beverages	0.000	0.001		0.000	0.001		-0.001	0.002		-0.001	0.003		0.000	0.001	
Numeraire category	0.001	-0.033		-0.001	-0.028		-0.004	-0.058		-0.002	-0.094		-0.001	-0.032	
**Total expenditure**	0.001	0.000	*	0.000	0.000		-0.006	0.001	*	-0.017	0.001	*	-0.002	0.000	*
**Promotions**															
Take home confectionery	0.000	0.000		0.000	0.000		0.000	0.000		-0.001	0.000		0.000	0.000	
Biscuits	0.000	0.000	*	0.000	0.000	*	0.000	0.000		0.000	0.000		0.000	0.000	*
Take home savouries	0.000	0.000		0.000	0.000		0.000	0.000	*	0.001	0.000	*	0.000	0.000	
Cakes, pastries, and sugar morning goo	0.000	0.000		0.000	0.000		0.001	0.000	*	0.000	0.000		0.000	0.000	*
Total puddings and desserts	0.000	0.000	*	0.000	0.000	*	0.000	0.000		-0.001	0.000	*	0.000	0.000	*
Take home sugary drinks	0.002	0.000	*	0.000	0.000		-0.001	0.000	*	0.000	0.000	*	0.000	0.000	*
Edible ices and ice creams	0.000	0.000		0.003	0.000	*	-0.001	0.000	*	-0.001	0.000		0.000	0.000	*
Dairy products	0.000	0.000		0.000	0.000	*	0.002	0.000	*	0.000	0.000	*	0.000	0.000	
Meat and fish	0.000	0.000		0.000	0.000	*	-0.001	0.000	*	0.007	0.000	*	0.000	0.000	
Fats and eggs	0.000	0.000	*	0.000	0.000	*	0.000	0.000		0.002	0.000	*	0.002	0.000	*
Fruit	0.000	0.000	*	0.000	0.000		0.000	0.000		-0.001	0.000	*	0.000	0.000	*
Vegetables	0.000	0.000	*	0.000	0.000		0.001	0.000	*	0.001	0.001		0.000	0.000	
Grains	0.000	0.000	*	0.000	0.000		0.001	0.000	*	0.000	0.000		0.000	0.000	
Prepared ready to eat foods	0.000	0.000		0.000	0.000		0.000	0.000		-0.004	0.001	*	-0.001	0.000	*
Sugar and preserves	0.000	0.000		0.000	0.000		-0.001	0.000	*	0.000	0.001		0.000	0.000	
Condiments and sauces	0.000	0.000		0.000	0.000		0.000	0.001		0.003	0.001	*	0.000	0.000	*
Low calorie, soft drinks and juices	0.000	0.000		0.000	0.000		0.000	0.000	*	0.000	0.000	*	0.000	0.000	*
Alcoholic beverages	-0.001	0.000	*	0.000	0.000	*	-0.002	0.000	*	-0.001	0.000	*	0.000	0.000	*
Numeraire category	-0.001	0.000	*	-0.001	0.000	*	-0.001	0.000	*	0.000	0.001		0.000	0.000	*

Equations (columns 11–15 of 19)

Variables	Fruit			Vegetables			Grains			Prepared ready to eat foods			Sugar and preserves		
	Coef.	St. Err.	Sig.	Coef.	St. Err.	Sig.	Coef.	St. Err.	Sig.	Coef.	St. Err.	Sig.	Coef.	St. Err.	Sig.
**Intercept**	0.071	0.010	*	0.088	0.009	*	0.029	0.006	*	0.071	0.016	*	0.014	0.003	*
**Prices**															
Take home confectionery	0.018	0.002	*	0.009	0.002	*	-0.003	0.001	*	-0.003	0.004		0.001	0.001	
Biscuits	0.005	0.003	*	-0.003	0.003		0.005	0.002	*	-0.001	0.005		0.004	0.001	*
Take home savouries	-0.004	0.002	*	0.005	0.002	*	0.003	0.002	*	-0.003	0.005		0.000	0.001	
Cakes, pastries, and sugar morning goo	0.002	0.003		0.005	0.003	*	0.003	0.002		-0.019	0.005	*	0.001	0.001	
Total puddings and desserts	-0.002	0.002		0.002	0.002		-0.003	0.001	*	0.011	0.003	*	0.000	0.001	
Take home sugary drinks	0.007	0.003	*	0.006	0.002	*	-0.002	0.002		0.004	0.004		0.000	0.001	
Edible ices and ice creams	0.002	0.002		0.001	0.002		-0.003	0.001	*	-0.007	0.004	*	-0.001	0.001	
Dairy products	0.001	0.004		-0.006	0.004		-0.002	0.003		0.007	0.007		-0.002	0.001	
Meat and fish	0.002	0.007		-0.019	0.006	*	0.016	0.004	*	0.032	0.012	*	0.003	0.002	
Fats and eggs	-0.011	0.002	*	-0.008	0.002	*	-0.001	0.002		0.005	0.004		-0.001	0.001	
Fruit	-0.013	0.006	*	-0.017	0.004	*	-0.006	0.003	*	0.011	0.007		0.000	0.001	
Vegetables	-0.017	0.004	*	-0.003	0.005		-0.002	0.002		0.028	0.007	*	-0.004	0.001	*
Grains	-0.006	0.003	*	-0.002	0.002		0.002	0.003		-0.002	0.005		-0.001	0.001	
Prepared ready to eat foods	0.011	0.007		0.028	0.007	*	-0.002	0.005		-0.055	0.017	*	0.005	0.002	*
Sugar and preserves	0.000	0.001		-0.004	0.001	*	-0.001	0.001		0.005	0.002	*	-0.003	0.001	*
Condiments and sauces	-0.001	0.001		0.002	0.001		0.001	0.001		-0.001	0.002		0.000	0.000	
Low calorie, soft drinks and juices	0.002	0.002		0.003	0.002	*	0.000	0.001		-0.005	0.004		0.001	0.001	
Alcoholic beverages	0.002	0.002		-0.002	0.001		-0.002	0.001	*	0.001	0.002		-0.001	0.000	
Numeraire category	0.002	-0.058		0.001	-0.051		-0.003	-0.037		-0.005	-0.098		-0.001	-0.017	
**Total expenditure**	0.004	0.001	*	-0.002	0.001	*	-0.008	0.000	*	-0.011	0.001	*	-0.001	0.000	*
**Promotions**															
Take home confectionery	0.000	0.000		-0.001	0.000	*	0.001	0.000	*	0.001	0.000	*	0.000	0.000	*
Biscuits	-0.001	0.000	*	-0.001	0.000	*	0.000	0.000	*	0.001	0.000	*	0.000	0.000	*
Take home savouries	-0.001	0.000	*	-0.001	0.000	*	0.000	0.000	*	0.000	0.000		0.000	0.000	
Cakes, pastries, and sugar morning goo	0.000	0.000		0.000	0.000		0.000	0.000		0.000	0.000		0.000	0.000	
Total puddings and desserts	0.000	0.000		-0.001	0.000	*	0.000	0.000		0.001	0.000	*	0.000	0.000	
Take home sugary drinks	-0.001	0.000	*	-0.001	0.000	*	0.000	0.000	*	0.000	0.000		0.000	0.000	
Edible ices and ice creams	0.000	0.000		0.000	0.000	*	0.000	0.000		0.000	0.000		0.000	0.000	
Dairy products	0.000	0.000		0.000	0.000		0.000	0.000	*	-0.001	0.000	*	0.000	0.000	
Meat and fish	0.000	0.000		0.000	0.000		0.000	0.000	*	0.000	0.000		0.000	0.000	*
Fats and eggs	-0.001	0.000	*	0.000	0.000		0.000	0.000	*	-0.001	0.000	*	0.000	0.000	
Fruit	0.006	0.000	*	0.001	0.000	*	0.000	0.000		0.000	0.000		0.000	0.000	*
Vegetables	0.002	0.000	*	0.004	0.000	*	0.000	0.000		-0.001	0.000	*	0.000	0.000	*
Grains	0.000	0.000		0.000	0.000	*	0.002	0.000	*	0.000	0.000		0.000	0.000	
Prepared ready to eat foods	-0.002	0.000	*	-0.001	0.000	*	0.001	0.000	*	0.010	0.000	*	0.000	0.000	
Sugar and preserves	-0.001	0.000	*	-0.002	0.000	*	0.000	0.000		0.000	0.001		0.003	0.000	*
Condiments and sauces	0.002	0.001	*	0.002	0.000	*	0.000	0.000		0.001	0.001		0.000	0.000	*
Low calorie, soft drinks and juices	0.000	0.000	*	0.000	0.000		0.000	0.000	*	0.000	0.000		0.000	0.000	*
Alcoholic beverages	-0.002	0.000	*	-0.001	0.000	*	-0.002	0.000	*	-0.003	0.000	*	-0.001	0.000	*
Numeraire category	-0.003	0.000	*	-0.001	0.000	*	-0.001	0.000	*	0.000	0.001		0.000	0.000	*

Equations (columns 16–19 of 19)

Variables	Condiments and sauces			Low calorie soft drinks and juices			Alcoholic beverages			Numeraire category		
	Coef.	St. Err.	Sig.	Coef.	St. Err.	Sig.	Coef.	St. Err.	Sig.	Coef.	St. Err.	Sig.
**Intercept**	0.022	0.003	*	0.041	0.005	*	0.147	0.007	*	0.143	0.035	*
**Prices**												
Take home confectionery	0.000	0.001		-0.001	0.001		0.003	0.001	*	0.000	0.008	
Biscuits	0.000	0.001		0.002	0.001		-0.001	0.001		-0.001	0.011	
Take home savouries	-0.001	0.001		0.000	0.001		0.000	0.001		-0.002	0.010	
Cakes, pastries, and sugar morning goo	0.001	0.001		-0.006	0.001	*	0.000	0.001		-0.002	0.010	
Total puddings and desserts	0.000	0.000		0.001	0.001		0.000	0.000		0.000	0.007	
Take home sugary drinks	-0.001	0.001		0.002	0.001		0.000	0.001		0.001	0.009	
Edible ices and ice creams	0.000	0.001		0.003	0.001	*	0.000	0.001		-0.001	0.007	
Dairy products	-0.001	0.001		0.009	0.002	*	-0.001	0.002		-0.004	0.016	
Meat and fish	0.000	0.002		-0.010	0.004	*	-0.001	0.003		-0.002	0.026	
Fats and eggs	0.001	0.001	*	0.002	0.001		0.000	0.001		-0.001	0.009	
Fruit	-0.001	0.001		0.002	0.002		0.002	0.002		0.002	0.016	
Vegetables	0.002	0.001		0.003	0.002	*	-0.002	0.001		0.001	0.014	
Grains	0.001	0.001		0.000	0.001		-0.002	0.001	*	-0.003	0.010	
Prepared ready to eat foods	-0.001	0.002		-0.005	0.004		0.001	0.002		-0.005	0.028	
Sugar and preserves	0.000	0.000		0.001	0.001		-0.001	0.000		-0.001	0.004	
Condiments and sauces	0.000	0.000		0.001	0.001		-0.001	0.000		-0.001	0.004	
Low calorie, soft drinks and juices	0.001	0.001		0.001	0.002		-0.002	0.001		-0.002	0.008	
Alcoholic beverages	-0.001	0.000		-0.002	0.001		-0.006	0.005		0.010	0.007	
Numeraire category	-0.001	-0.015		-0.002	-0.030		0.010	-0.024		0.010	0.200	
**Total expenditure**	-0.002	0.000	*	-0.003	0.001	*	-0.001	0.002		0.051	0.004	*
**Promotions**												
Take home confectionery	0.000	0.000		0.000	0.000		-0.005	0.001	*	-0.001	0.001	
Biscuits	0.000	0.000		0.000	0.000		-0.003	0.001	*	0.001	0.001	
Take home savouries	0.000	0.000		0.000	0.000		0.000	0.001		-0.001	0.001	
Cakes, pastries, and sugar morning goo	0.000	0.000	*	0.000	0.000		-0.001	0.001	*	0.000	0.001	
Total puddings and desserts	0.000	0.000	*	0.001	0.000	*	-0.003	0.001	*	-0.001	0.001	
Take home sugary drinks	0.000	0.000	*	-0.001	0.000	*	0.001	0.000	*	-0.001	0.001	
Edible ices and ice creams	0.000	0.000	*	0.000	0.000	*	-0.002	0.001	*	-0.001	0.001	
Dairy products	0.000	0.000		0.000	0.000		-0.002	0.000	*	0.000	0.001	
Meat and fish	0.000	0.000		-0.001	0.000	*	-0.002	0.001	*	-0.001	0.001	
Fats and eggs	0.000	0.000		0.000	0.000	*	0.000	0.001		-0.001	0.001	
Fruit	0.000	0.000		0.000	0.000	*	-0.001	0.001	*	-0.002	0.001	*
Vegetables	0.000	0.000		0.000	0.000	*	-0.001	0.001		-0.002	0.001	
Grains	0.000	0.000		0.000	0.000		-0.001	0.001		0.000	0.001	
Prepared ready to eat foods	0.000	0.000	*	0.001	0.000	*	-0.003	0.001	*	0.000	0.002	
Sugar and preserves	-0.001	0.000	*	0.001	0.000	*	-0.002	0.001	*	0.001	0.002	
Condiments and sauces	0.004	0.000	*	0.000	0.000		-0.005	0.002	*	-0.004	0.002	
Low calorie, soft drinks and juices	0.000	0.000		0.002	0.000	*	0.000	0.000		-0.001	0.000	*
Alcoholic beverages	0.000	0.000	*	-0.001	0.000	*	0.022	0.001	*	-0.001	0.001	
Numeraire category	0.000	0.000	*	0.000	0.000		0.000	0.001		0.012	0.002	*

Note: '*' indicates statistically significant at 5 per cent.

Table S12 - Scotland – Price equations

Variables	Take home confectionery			Biscuits			Take home savouries			Cakes, pastries and sugar morning goods			Total puddings and desserts			Take home sugary drinks			Edible ices and ice cream			Dairy products			Meat and fish		
	Coef.	St. Err.	Sig.	Coef.	St. Err.	Sig.	Coef.	St. Err.	Sig.	Coef.	St. Err.	Sig.	Coef.	St. Err.	Sig.	Coef.	St. Err.	Sig.	Coef.	St. Err.	Sig.	Coef.	St. Err.	Sig.	Coef.	St. Err.	Sig.
Quantity	0.4776	0.0189	*	0.1533	0.0111	*	0.0878	0.0076	*	0.2593	0.0135	*	0.2440	0.0120	*	0.2012	0.0095	*	0.1883	0.0107	*	0.3071	0.0226	*	0.1753	0.0137	*
Age	0.0036	0.0008	*	-0.0049	0.0003	*	0.0012	0.0003	*	-0.0072	0.0005	*	-0.0047	0.0005	*	0.0011	0.0005	*	-0.0087	0.0005	*	-0.0022	0.0004	*	0.0003	0.0003	
Number of kids	0.0397	0.0134	*	0.0115	0.0047	*	-0.0242	0.0037	*	0.0989	0.0067	*	-0.0022	0.0080		0.0028	0.0080		-0.0100	0.0067		0.0566	0.0076	*	-0.0201	0.0046	*
Number of adults	0.0217	0.0049	*	0.0085	0.0018	*	0.0035	0.0014	*	0.0220	0.0025	*	0.0148	0.0029	*	0.0069	0.0029	*	0.0104	0.0025	*	0.0237	0.0029	*	0.0062	0.0017	*

Variables	Fats and eggs			Fruit			Vegetables			Grains			Prepared ready to eat foods			Sugar and preserves			Condiments and sauces			Low calorie soft drinks and juices			Alcoholic beverages		
	Coef.	St. Err.	Sig.	Coef.	St. Err.	Sig.	Coef.	St. Err.	Sig.	Coef.	St. Err.	Sig.	Coef.	St. Err.	Sig.	Coef.	St. Err.	Sig.	Coef.	St. Err.	Sig.	Coef.	St. Err.	Sig.	Coef.	St. Err.	Sig.
Quantity	0.2738	0.0153	*	0.2225	0.0119	*	0.3484	0.0192	*	0.4762	0.0324	*	0.1663	0.0143	*	0.8857	0.0286	*	1.1759	0.0480	*	0.4803	0.0204	*	0.2378	0.0084	*
Age	0.0025	0.0006	*	-0.0032	0.0004	*	-0.0041	0.0004	*	-0.0026	0.0004	*	0.0007	0.0003	*	-0.0217	0.0010	*	0.0033	0.0010	*	0.0146	0.0010	*	0.0030	0.0006	*
Number of kids	0.0227	0.0092	*	0.0237	0.0065	*	0.0281	0.0072	*	0.0369	0.0072	*	-0.0128	0.0045	*	0.2382	0.0149	*	0.0984	0.0169	*	-0.0064	0.0150		0.0302	0.0104	*
Number of adults	0.0150	0.0034	*	0.0169	0.0025	*	0.0137	0.0025	*	0.0286	0.0031	*	0.0076	0.0017	*	0.0504	0.0054	*	0.0523	0.0061	*	0.0243	0.0055	*	-0.0004	0.0036	*

Variables	Numeraire category		
	Coef.	St. Err.	Sig.
Quantity	1.7279	0.0542	*
Age	-0.0074	0.0014	*
Number of kids	0.3019	0.0229	*
Number of adults	0.1229	0.0085	*

Note: '*' indicates statistically significant at 5 per cent.

Table S13 - Take home confectionery - Analysis of promotions by shares and growth rate

	Years					
	2013	2014	2015	2016	2017	2018
**Shares (%)**	100.0	100.0	100.0	100.0	100.0	100.0
Full price	51.6	52.2	52.3	54.1	58.1	59.0
Temporary price reduction	36.9	37.1	38.0	36.2	33.2	32.9
Multibuy	0.9	1.0	0.4	0.6	0.3	0.3
Y for £X	9.8	9.1	8.8	8.6	8.1	7.7
Other promotions	0.7	0.5	0.5	0.5	0.3	0.2
**Growth rate (%)**	**2013/12**	**2014/13**	**2015/14**	**2016/15**	**2017/16**	**2018/17**
Purchases	..	1.9	8.8	-1.7	16.7	2.9
Full price	..	3.2	9.0	1.6	25.3	4.5
Temporary price reduction	..	2.4	11.6	-6.4	7.0	1.8
Multibuy	..	8.0	-56.9	38.3	-41.9	-3.8
Y for £X	..	-5.3	4.4	-3.5	9.8	-2.4
Other promotions	..	-20.9	6.7	-0.5	-31.1	-40.8
**Contribution to growth (%)**	..	1.9	8.8	-1.7	16.7	2.9
Full price	..	1.7	4.7	0.9	14.7	2.7
Temporary price reduction	..	0.9	4.4	-2.3	2.3	0.6
Multibuy	..	0.1	-0.2	0.2	-0.1	0.0
Y for £X	..	-0.5	0.4	-0.3	0.8	-0.2
Other promotions	..	-0.1	0.0	0.0	-0.1	-0.1

Source: Own elaboration based on Kantar Worldpanel data.

Table S14 - Biscuits - Analysis of promotions by shares and growth rate

	Years					
	2013	2014	2015	2016	2017	2018
**Shares (%)**	100.0	100.0	100.0	100.0	100.0	100.0
Full price	56.5	54.2	51.7	55.2	59.7	60.5
Temporary price reduction	35.7	35.2	36.3	37.1	35.3	34.0
Multibuy	1.4	0.5	0.4	0.1	0.2	0.9
Y for £X	5.4	9.2	10.8	6.7	4.0	4.2
Other promotions	1.0	1.0	0.8	0.9	0.8	0.5
**Growth rate (%)**	**2013/12**	**2014/13**	**2015/14**	**2016/15**	**2017/16**	**2018/17**
Purchases	..	-0.9	6.2	-6.2	15.3	-0.7
Full price	..	-5.0	1.3	0.1	24.8	0.5
Temporary price reduction	..	-2.5	9.6	-4.1	9.8	-4.4
Multibuy	..	-65.8	-6.5	-73.4	93.9	327.1
Y for £X	..	68.5	24.5	-41.3	-31.6	5.1
Other promotions	..	-1.8	-12.1	-0.6	4.4	-43.2
**Contribution to growth (%)**	..	-0.9	6.2	-6.2	15.3	-0.7
Full price	..	-2.7	0.7	0.1	14.8	0.3
Temporary price reduction	..	-0.9	3.5	-1.5	3.4	-1.5
Multibuy	..	-0.3	0.0	-0.1	0.2	2.8
Y for £X	..	6.3	2.6	-2.8	-1.3	0.2
Other promotions	..	0.0	-0.1	0.0	0.0	-0.2

Source: Own elaboration based on Kantar Worldpanel data.

Table S15 - Take home savouries - Analysis of promotions by shares and growth rate

	Years					
	2013	2014	2015	2016	2017	2018
**Shares (%)**	100.0	100.0	100.0	100.0	100.0	100.0
Full price	48.8	47.4	48.9	48.3	52.8	54.2
Temporary price reduction	29.1	31.0	35.5	38.6	35.9	35.2
Multibuy	3.7	3.6	0.9	0.4	0.2	0.0
Y for £X	17.4	17.1	14.3	11.9	10.3	9.9
Other promotions	1.0	0.9	0.4	0.8	0.9	0.6
**Growth rate (%)**	**2013/12**	**2014/13**	**2015/14**	**2016/15**	**2017/16**	**2018/17**
Purchases	..	1.7	9.6	-7.2	12.8	7.7
Full price	..	-1.1	13.0	-8.3	23.2	10.6
Temporary price reduction	..	8.4	25.4	1.0	4.8	5.7
Multibuy	..	-1.1	-73.0	-62.5	-44.9	-72.3
Y for £X	..	-0.2	-8.4	-22.8	-2.6	3.7
Other promotions	..	-12.9	-44.0	65.2	30.4	-27.1
**Contribution to growth (%)**	..	1.7	9.6	-7.2	12.8	7.7
Full price	..	-0.5	6.4	-4.0	12.3	5.7
Temporary price reduction	..	2.6	9.0	0.4	1.7	2.0
Multibuy	..	0.0	-0.6	-0.2	-0.1	0.0
Y for £X	..	0.0	-1.2	-2.7	-0.3	0.4
Other promotions	..	-0.1	-0.2	0.5	0.3	-0.2

Source: Own elaboration based on Kantar Worldpanel data.

Table S16 - Ambient cakes and pastries - Analysis of promotions by shares and growth rate

	Years					
	2013	2014	2015	2016	2017	2018
**Shares (%)**	100.0	100.0	100.0	100.0	100.0	100.0
Full price	71.6	71.6	71.0	73.2	75.2	77.1
Temporary price reduction	15.5	15.4	18.5	19.0	18.0	16.4
Multibuy	0.4	0.6	0.2	0.3	0.3	0.3
Y for £X	12.0	11.9	10.1	7.3	6.4	6.0
Other promotions	0.5	0.5	0.2	0.2	0.1	0.1
**Growth rate (%)**	**2013/12**	**2014/13**	**2015/14**	**2016/15**	**2017/16**	**2018/17**
Purchases	..	-1.1	11.8	-4.6	12.7	2.0
Full price	..	-1.1	10.8	-1.6	15.7	4.6
Temporary price reduction	..	-1.5	33.9	-1.7	6.4	-6.6
Multibuy	..	25.8	-51.1	-2.2	41.8	4.6
Y for £X	..	-1.6	-5.0	-30.7	-1.5	-4.0
Other promotions	..	-4.3	-47.2	-21.3	-18.0	-11.6
**Contribution to growth (%)**	..	-1.1	11.8	-4.6	12.7	2.0
Full price	..	-0.8	7.7	-1.2	11.8	3.5
Temporary price reduction	..	-0.2	6.3	-0.3	1.2	-1.1
Multibuy	..	0.1	-0.1	0.0	0.1	0.0
Y for £X	..	-0.2	-0.5	-2.3	-0.1	-0.2
Other promotions	..	0.0	-0.1	0.0	0.0	0.0

Source: Own elaboration based on Kantar Worldpanel data.

Table S17 - Total puddings and desserts - Analysis of promotions by shares and growth rate

	Years					
	2013	2014	2015	2016	2017	2018
**Shares (%)**	100.0	100.0	100.0	100.0	100.0	100.0
Full price	57.5	56.4	56.4	57.7	61.0	62.8
Temporary price reduction	21.2	22.7	24.3	25.3	22.4	24.6
Multibuy	2.3	1.1	0.6	0.9	1.8	1.4
Y for £X	15.2	15.8	14.2	11.0	9.3	8.5
Other promotions	3.7	4.0	4.5	5.1	5.5	2.6
**Growth rate (%)**	**2013/12**	**2014/13**	**2015/14**	**2016/15**	**2017/16**	**2018/17**
Purchases	..	2.8	11.2	-5.3	11.7	-3.2
Full price	..	0.7	11.2	-3.1	18.1	-0.4
Temporary price reduction	..	9.6	19.3	-1.5	-1.2	6.5
Multibuy	..	-49.2	-44.2	43.6	135.7	-24.5
Y for £X	..	7.0	0.1	-27.0	-5.8	-11.0
Other promotions	..	10.1	24.1	8.5	19.0	-53.2
**Contribution to growth (%)**	..	2.8	11.2	-5.3	11.7	-3.2
Full price	..	0.4	6.3	-1.8	11.0	-0.3
Temporary price reduction	..	2.2	4.7	-0.4	-0.3	1.6
Multibuy	..	-0.6	-0.3	0.4	2.5	-0.4
Y for £X	..	1.1	0.0	-3.0	-0.5	-0.9
Other promotions	..	0.4	1.1	0.4	1.0	-1.4

Source: Own elaboration based on Kantar Worldpanel data.

Table S18 - Regular soft drinks - Analysis of promotions by shares and growth rate

	Years					
	2013	2014	2015	2016	2017	2018
**Shares (%)**	100.0	100.0	100.0	100.0	100.0	100.0
Full price	46.8	46.5	45.6	47.6	53.3	57.6
Temporary price reduction	26.0	25.6	27.2	30.2	31.4	30.0
Multibuy	4.4	2.7	1.9	0.2	0.1	0.0
Y for £X	21.4	23.6	24.1	20.6	13.8	11.0
Other promotions	1.3	1.5	1.1	1.4	1.4	1.3
**Growth rate (%)**	**2013/12**	**2014/13**	**2015/14**	**2016/15**	**2017/16**	**2018/17**
Purchases	..	-0.4	5.6	-2.0	13.4	8.7
Full price	..	-1.0	3.5	2.3	27.0	17.6
Temporary price reduction	..	-1.9	12.3	8.6	18.1	3.9
Multibuy	..	-37.9	-26.1	-87.8	-70.3	-55.3
Y for £X	..	9.9	7.7	-16.3	-23.9	-13.5
Other promotions	..	13.4	-21.1	21.1	13.6	2.5
**Contribution to growth (%)**	..	-0.4	5.6	-2.0	13.4	8.7
Full price	..	-0.5	1.6	1.1	14.4	10.1
Temporary price reduction	..	-0.5	3.4	2.6	5.7	1.2
Multibuy	..	-1.0	-0.5	-0.2	0.0	0.0
Y for £X	..	2.3	1.9	-3.4	-3.3	-1.5
Other promotions	..	0.2	-0.2	0.3	0.2	0.0

Source: Own elaboration based on Kantar Worldpanel data.

Table S19 - Edible ices and ice cream - Analysis of promotions by shares and growth rate

	Years					
	2013	2014	2015	2016	2017	2018
**Shares (%)**	100.0	100.0	100.0	100.0	100.0	100.0
Full price	52.3	54.7	52.3	53.6	53.5	52.6
Temporary price reduction	33.2	29.9	33.0	36.0	35.4	33.8
Multibuy	0.1	0.4	0.1	0.1	0.0	0.1
Y for £X	13.6	14.2	12.7	8.4	9.5	12.3
Other promotions	0.8	0.8	1.9	1.9	1.5	1.2
**Growth rate (%)**	**2013/12**	**2014/13**	**2015/14**	**2016/15**	**2017/16**	**2018/17**
Purchases	..	5.4	7.4	-3.3	14.2	8.0
Full price	..	10.3	2.7	-1.1	14.2	6.2
Temporary price reduction	..	-5.1	18.4	5.5	12.5	3.1
Multibuy	..	703.8	-83.2	98.1	-74.1	89.7
Y for £X	..	9.4	-3.4	-35.9	29.2	38.5
Other promotions	..	8.0	155.6	-3.8	-11.9	-9.8
**Contribution to growth (%)**	..	5.4	7.4	-3.3	14.2	8.0
Full price	..	5.6	1.4	-0.6	7.6	3.3
Temporary price reduction	..	-1.5	6.1	2.0	4.4	1.0
Multibuy	..	2.9	-0.1	0.1	0.0	0.0
Y for £X	..	1.3	-0.4	-3.0	2.8	4.7
Other promotions	..	0.1	2.9	-0.1	-0.2	-0.1

Source: Own elaboration based on Kantar Worldpanel data.
